# Parkin activates innate immunity and promotes antitumor immune responses

**DOI:** 10.1172/JCI180983

**Published:** 2024-08-30

**Authors:** Michela Perego, Minjeong Yeon, Ekta Agarwal, Andrew T. Milcarek, Irene Bertolini, Chiara Camisaschi, Jagadish C. Ghosh, Hsin-Yao Tang, Nathalie Grandvaux, Marcus Ruscetti, Andrew V. Kossenkov, Sarah Preston-Alp, Italo Tempera, Noam Auslander, Dario C. Altieri

**Affiliations:** 1 Immunology, Microenvironment and Metastasis Program, The Wistar Institute, Philadelphia, Pennsylvania, USA.; 2 Flow Cytometry Core, IRCCS Humanitas Research Hospital, Rozzano, Milan, Italy.; 3 Center for Systems and Computational Biology and; 4 Proteomics and Metabolomics Shared Resource, The Wistar Institute, Philadelphia, Pennsylvania, USA.; 5 CRCHUM — Centre de Recherche du Centre Hospitalier de l’Université de Montréal, Montréal, Québec, Canada.; 6 Department of Biochemistry and Molecular Medicine, Faculty of Medicine, Université de Montréal, Montréal, Québec, Canada.; 7 Department of Molecular, Cell and Cancer Biology, University of Massachusetts Chan Medical School, Worcester, Massachusetts, USA.; 8 Bioinformatics Shared Resource,; 9 Gene Expression and Regulation Program,; 10 Molecular and Cellular Oncogenesis Program, The Wistar Institute, Philadelphia, Pennsylvania, USA.

**Keywords:** Immunology, Oncology, Innate immunity, Mitochondria, Tumor suppressors

## Abstract

The activation of innate immunity and associated interferon (IFN) signaling have been implicated in cancer, but the regulators are elusive and links to tumor suppression remain undetermined. Here, we found that Parkin, an E3 ubiquitin ligase altered in Parkinson’s Disease, was epigenetically silenced in cancer and its reexpression by clinically approved demethylating therapy stimulated transcription of a potent IFN response in tumor cells. This pathway required Parkin E3 ubiquitin ligase activity, involved the subcellular trafficking and release of the alarmin High Mobility Group Box 1 (HMGB1) and was associated with inhibition of NF-κB gene expression. In turn, Parkin-expressing cells released an IFN secretome that upregulated effector and cytotoxic CD8^+^ T cell markers, lowered the expression of immune inhibitory receptors TIM3 and LAG3, and stimulated high content of the self renewal/stem cell factor, TCF1. PRKN-induced CD8^+^ T cells selectively accumulated in the microenvironment and inhibited transgenic and syngeneic tumor growth in vivo. Therefore, Parkin is an epigenetically regulated activator of innate immunity and dual mode tumor suppressor, inhibiting intrinsic tumor traits of metabolism and cell invasion, while simultaneously reinvigorating CD8 T cell functions in the microenvironment.

## Abstract 2

Parkin functions as a tumor suppressor by stimulating interferon signaling and reinvigorating effector and cytotoxic CD8 T cells in the microenvironment

## Graphical abstract

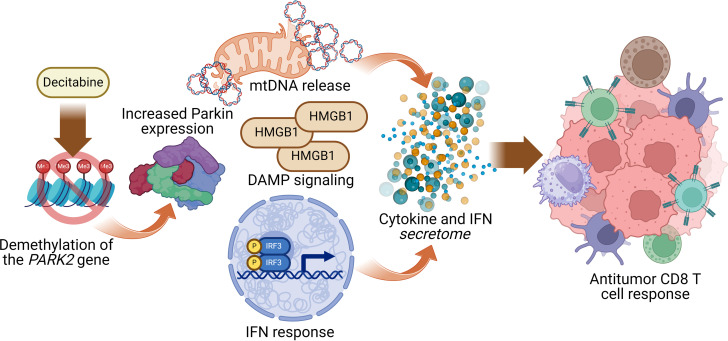

## Introduction

The transcriptional induction of interferons (IFN) and their downstream IFN-stimulated genes (ISG) is a formidable host defense mechanism against pathogenic infections, mostly viruses (1). Identified as innate immunity, this pathway involves the release of damage-associated molecular pattern (DAMP) and pathogen-associated molecular pattern (PAMP) (2) in response to cellular stress (3), mitochondrial damage (4, 5), and exposure to infectious pathogens (6). In turn, DAMP/PAMP signaling activates a multifaceted danger sensing machinery in cytosol, including the cGAS/STING complex (7, 8), as well as mitochondria (9), which leads to the assembly of an IRF3/STAT1 transcriptional complex driving the expression of multiple IFN molecules, ISG, and pleiotropic inflammatory cytokines (10).

In addition to protection against pathogens, there is evidence that innate immunity and IFN signaling have important roles in cancer, modulating a host of tumor responses, including sensitivity to immunotherapy (11, 12). This pathway is complex and highly context dependent (13). As an antitumor mechanism (11, 12), IFN signaling promotes intratumoral recruitment of effector CD8^+^ T cells (14), activation of MHC class I dendritic cells (15), and improved response to conventional (16), molecular (17), and immune therapy (18). In fact, IFN therapy is feasible, generally well tolerated, and accompanied by positive patient responses alone or in combination with immunotherapy (19, 20). On the other hand, sustained, i.e. chronic, IFN activation can be highly detrimental in cancer, especially contributing to CD8^+^ T cell exhaustion (21), a process marked by progressive loss of effector functions and insensitivity to therapeutic immune checkpoint inhibitors (ICI) (22). What controls the pro- or antitumorigenic responses to IFN signaling remains elusive, and endogenous regulators of this process potentially linked to tumor suppression have not been identified.

One candidate molecule at the interface between cancer and innate immunity is Parkin (PRKN), an E3 ubiquitin ligase implicated in mitochondrial quality control via mitophagy (23). Defects in this pathway leading to the accumulation of damaged and dysfunctional mitochondria have been linked to neuronal toxicity in patients with Parkinson’s Disease (PD), where the *PARK2* gene encoding PRKN can be mono- or biallelically altered (24, 25). In addition, PRKN has been suggested to inhibit multiple mechanisms of innate immunity, including mitochondrial antigen presentation (26), STING signaling (27), and activation of the NLRP3 inflammasome (28). In this scenario, loss of PRKN would contribute to neuroinflammation, another invariable hallmark of PD pathogenesis (29).

On the other hand, it is clear that PRKN has functions beyond the CNS. For instance, PRKN expression is undetectable in virtually all examined human cancers and tumor cell lines (30), suggesting a general role in tumor suppression (31). How this is orchestrated remains to be elucidated, but reintroduction of PRKN in different cancer types is sufficient to inhibit several intrinsic tumor traits, such as mitotic transitions (32), metabolic reprogramming through the pentose phosphate pathway (30), and phosphoglycerate dehydrogenase (33), as well as heightened cell motility and invasion cell motility and invasion (34). While these responses were independent of mitophagy, a role of PRKN innate immunity (26–28) in cancer is unknown and a potential link of this pathway to tumor suppression has not been considered. In this study, we explored a role of PRKN in antitumor immunity.

## Results

### PRKN activates IFN gene expression in cancer.

**Figure 1:**
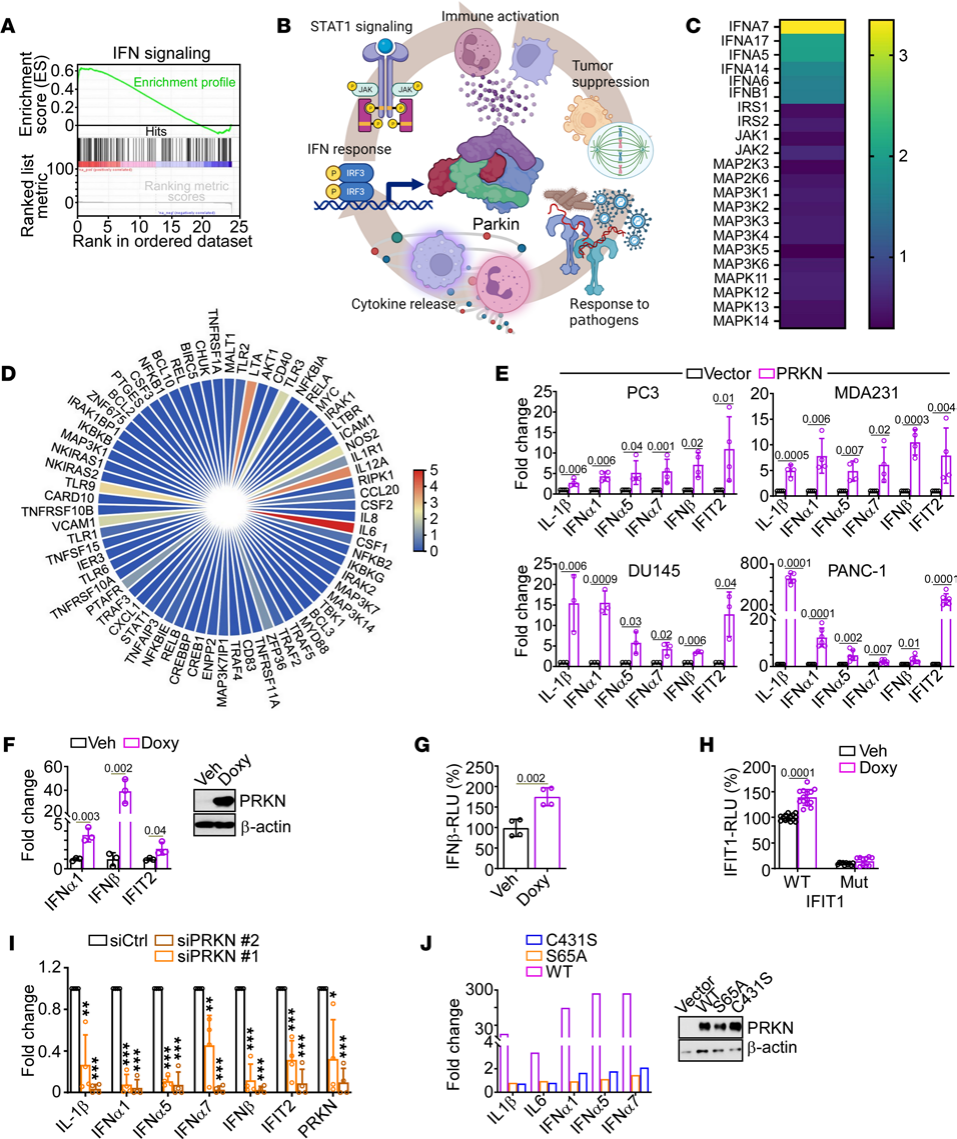
PRKN IFN response in cancer. (**A**) PC3 cells were transfected with vector or PRKN and analyzed for an IFN enrichment gene signature by RNA-Seq. (**B**) Schematic diagram of innate immunity pathways activated by PRKN in PC3 cells by RNA-Seq. Created with BioRender.com. (**C**) PC3 cells expressing PRKN (as in **A**) were analyzed in an IFN/MAPK array by RT-qPCR. Heatmap from a representative experiment. (**D**) The conditions are the same as in **A** and PRKN-expressing PC3 cells were analyzed in an NF-κB gene array by RT-qPCR. Heatmap from a representative experiment out of 2 independent determinations. (**E**) The indicated tumor cell types expressing vector or PRKN were analyzed for IFN gene expression by RT-qPCR. Mean ± SD (*n* = 3). (**F**) PC3 cells that conditionally express PRKN (TetON system) in response to Doxycycline (Doxy) were analyzed by Western blotting (inset) and RT-qPCR in the presence of vehicle (Veh) or Doxy. Mean ± SD (*n* = 3). (**G**) PRKN TetON PC3 cells were analyzed for IFN-β promoter luciferase activity in the presence of vehicle (Veh) or Doxy. RLU, relative luciferase activity. Mean ± SD (*n* = 4). (**H**) The conditions are the same as in **G** except that PRKN TetON PC3 cells were analyzed for WT or mutant (Mut) IFIT1 promoter luciferase activity in the presence of vehicle (Veh) or Doxy. Mean ± SD (*n* = 3). (**I**) Normal breast epithelial MCF10A cells expressing endogenous PRKN were transfected with control nontargeted siRNA (siCtrl) or 2 independent siRNA sequences to PRKN (siPRKN #1 and siPRKN #2) and analyzed for IFN gene expression by RT-qPCR. Mean ± SD (*n* = 3). (**J**) PC3 cells expressing WT PRKN (WT) or E3-ligase defective PRKN C431S or S65A mutants (inset) were analyzed for IFN gene expression by RT-qPCR. Data are from a representative experiment out of 4 independent determinations. Numbers represent *P* values by 2-tailed unpaired *t* test. *P = 0.01; **P = 0.002–0.009; ***P = <0.0001–0.0003.

We began this study by asking whether reexpression of PRKN in tumor cells affected gene expression. By RNA-Seq profiling, we found that transient expression of PRKN in PRKN-negative prostate cancer PC3 cells (30) induced a potent IFN response (Figure 1, A and B) with upregulation of multiple IFNs, ISG, and pleiotropic cytokines (Supplemental Figure 1A; supplemental material available online with this article; https://doi.org/10.1172/JCI180983DS1). Consistent with tumor suppression, effectors of tumorigenesis (Myc, Myb, NKX2-3, and TRIM24), metastasis (NFE2L2), oncogenic transformation (FOXM1), and cell cycle (E2F2 and E2F3) were also inhibited in the presence of PRKN (Supplemental Figure 1B). Further bioinformatics analysis of this dataset showed that PRKN upregulated DNA damage and cell death responses, whereas intrinsic tumor traits of glycolysis, eIF2-α protein translation, and cell cycle were downregulated (Supplemental Figure 1C) (30). The PRKN transcriptome was specific because other pathways associated with inflammation, such as MAPK signaling (Figure 1C) and NF-κB–dependent gene expression (Figure 1D) were unchanged or profoundly suppressed, respectively (Figure 1, C and D). Consistent with this, PRKN expression inhibited NF-κB promoter reporter activity in PC3 cells (Supplemental Figure 1D), whereas several effectors of innate immunity (TLR3, TLR9, LTA, and IL12) were upregulated in the presence of PRKN (Figure 1D).

Next, we asked whether PRKN IFN gene expression was a general property of disparate tumor types. First, and consistent with recent findings (30), endogenous PRKN mRNA levels were mostly undetectable in a large panel of human and murine tumor cell lines (Supplemental Figure 1E). Conversely, normal mammary epithelial MCF10A cells expressed endogenous PRKN (Supplemental Figure 1E), in agreement with previous observations (30). Under these conditions, reintroduction of PRKN by transient transfection upregulated PRKN mRNA (Supplemental Figure 1F) and protein (Supplemental Figure 1G) levels in all human tumor types tested. This was associated with transcriptional induction of an IFN gene signature that comprised multiple IFN molecules, ISG, and inflammatory cytokines (Figure 1E). A similar response was observed in murine prostate cancer cell types MPTEN1 and MP3098, where transient reintroduction of PRKN potently upregulated IFN gene expression (Supplemental Figure 1H). To independently validate these findings, we next conditionally reexpressed PRKN in PC3 cells using a doxycycline-regulated (Doxy-regulated) TetON system (Figure 1F, *inset*). Here, treatment with Doxy induced a robust IFN response in stably transduced PC3 cells, whereas vehicle had no effect (Figure 1F). Consistent with these data, PRKN expression increased transcription of IFN-β (Figure 1G) and IFIT1 (Figure 1H) promoter reporter activity in PC3 cells, whereas an IFIT1 promoter mutant carrying a double mutation in the ISRE sites was not modulated by PRKN (Figure 1H). Finally, as a complementary approach, we next silenced endogenous PRKN in normal breast epithelial MCF10A cells (Supplemental Figure 1E) (30). In these cells, PRKN silencing using 2 independent siRNA sequences abolished IFN gene expression compared with control cultures (Figure 1I).

In addition to modulation of type I IFNs, IFN-α and IFN-β, RNA-Seq profiling of PRKN-expressing PC3 cells showed a prominent upregulation of IFN-γ (IFNG, Supplemental Figure 1A). Consistent with this, reexpression of PRKN in prostate cancer DU145 or pancreatic adenocarcinoma PANC-1 cells increased IFN-γ mRNA levels (Supplemental Figure 1I), whereas PRKN siRNA silencing in MCF10A cells suppressed IFN-γ expression (Supplemental Figure 1J). Finally, we asked if PRKN E3 ubiquitin ligase activity was required for IFN gene expression. In these experiments, expression of PRKN mutants (Figure 1J, inset) that abolish the E3 ligase catalytic site (Cys431Ser, C431S) or PINK1 phosphorylation site (Ser65Ala, S65A) required for E3 ligase function (23) did not induce an IFN response in PC3 cells (Figure 1J).

### PRKN methylation silencing in cancer.

**Figure 2:**
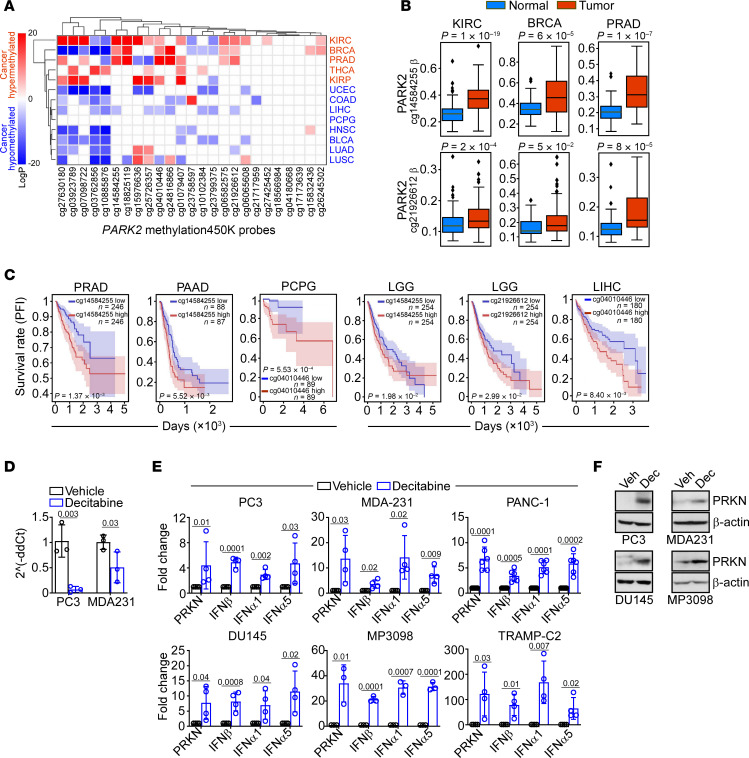
PRKN epigenetic silencing in human tumors. (**A**) Heatmap of *PARK2* gene methylation in cancer versus normal samples (TCGA). The individual probes are indicated. (**B**) Hypermethylation of *PARK2* promoter in cancer versus normal samples (TCGA). Boxes show the quartiles (0.25 and 0.75) of the data, center lines show the median, and whiskers show the rest of the distribution except for outliers (1-sided paired sample rank-sum test *P* values are reported). 2 methylation 450 K probes are used. A *P* value is indicated. KIRC, kidney clear cell carcinoma; BRCA, breast adenocarcinoma; PRAD, prostate adenocarcinoma. (**C**) Kaplan-Meier curves for *PARK2* hyper- or hypomethylation in patient cohorts (TCGA) of PRAD, pancreatic ductal adenocarcinoma (PAAD), liver hepatocellular carcinoma (LIHC), pheochromocytoma and paraganglioma (PCPG), or low-grade glioma (LGG, 2 independent *PARK2* methylation probes). A *P* value per patient cohort is indicated (2-tailed unpaired *t* test). (**D**) Methylation-specific PCR amplification of *PARK2* promoter region from PC3 or MDA231 cells approximately 200 bp upstream of the transcriptional start site in the presence or absence of the hypomethylating agent, decitabine. Mean ± SD (*n* = 3). (**E**) The indicated tumor cell lines were treated with vehicle or decitabine and analyzed for PRKN or IFN gene expression by RT-qPCR. Mean ± SD (*n* = 4). (**F**) The indicated human (PC3, DU145, MDA231) or murine (P3098) tumor cell lines were treated with vehicle (Veh) or decitabine (Dec) and analyzed by Western blotting. Numbers represent *P* values by 2-tailed unpaired *t* test.

In previous studies, PRKN loss in cancer was not accompanied by increased mutagenesis of the *PARK2* gene or copy number alterations (30). Instead, analysis of The Cancer Genome Atlas (TCGA) database demonstrated that the *PARK2* promoter was hypermethylated in select human tumors, including breast (BRCA) and prostate (PRAD) adenocarcinoma, as well as kidney cancer (KIRC), compared with normal tissues (Figure 2, A and B). Hypermethylation of the *PARK2* promoter correlated with shortened patient survival in prostate (PRAD) and pancreatic (PAAD) adenocarcinoma, low-grade glioma (LGG), liver hepatocellular carcinoma (LIHC) and paraganglioma/pheochromocytoma (PCPG) (Figure 2C). Consistent with epigenetic silencing, treatment of PC3 or MDA231 cells with a clinically approved pyrimidine nucleoside analog and DNA hypomethylating agent, decitabine, resulted in near complete demethylation of *PARK2* CpG promoter regions located at chr6:162728136 (reference genome hg38), approximately 200 bp upstream of the transcription start site (Figure 2D). Accordingly, decitabine treatment resulted in increased expression of endogenous PRKN mRNA (Figure 2E) and protein (Figure 2F) in multiple human and murine tumor cell types, whereas vehicle had no effect. As a result, decitabine-induced reexpression of endogenous PRKN potently upregulated IFN gene expression in all tumor types tested (Figure 2E).

### Mechanisms of PRKN activation of IFN gene expression.

Next, we studied the mechanism(s) of PRKN induction of IFN signaling in cancer. Consistent with models of innate immunity (7, 8), PRKN expression in PC3 cells activated the cytosolic danger-sensing cGAS pathway with increased production of the second messenger, 2′,3′ cGAMP (8) (Supplemental Figure 2A). This was accompanied by phosphorylation of the cGAMP downstream target, STING as well as IRF3 (Supplemental Figure 2B). In addition, PRKN-expressing PC3 cells exhibited increased phosphorylation of STAT1, a key transcriptional regulator of IFN signaling in a phosphoarray screen (Supplemental Figure 2, C and D). Conversely, PRKN inhibited the expression and phosphorylation of protumorigenic STAT3, as well as other STAT molecules, STAT2 and STAT5, compared with control (Supplemental Figure 2, C and D). Despite the upregulation of several proinflammatory cytokines, including IL1-β (IL1B, Supplemental Figure 1A), PRKN expression did not activate an NLRP3 inflammasome in PC3 cells, as formation of an ASC-AIM2 complex was unchanged by coimmunoprecipitation and Western blotting (Supplemental Figure 2E). Similarly, no proteolytic processing of caspase-1 was observed in the presence or absence of PRKN (Supplemental Figure 2E).

A potential requirement of the cGAS-STING pathway in PRKN IFN gene expression was next investigated. In these experiments, siRNA silencing of STING (Supplemental Figure 2F, left) did not affect PRKN levels (Supplemental Figure 2F, right), but abolished IFN gene expression in response to transient (Supplemental Figure 2G) or Doxy-induced conditional (Supplemental Figure 2H) PRKN expression. A negative regulator of STING gene expression during cellular senescence is the E3 ligase tripartite motif protein 30-α (TRIM30-α) (35). Differently from this model, however, reexpression of PRKN in tumor cell types (Supplemental Figure Supplemental Figure 2I, left) increased the levels of TRIM30-α mRNA (Supplemental Figure 2I, right). Finally, treatment of PC3 cells with a mitochondrial-directed superoxide scavenger, MitoTempo, abolished IFN gene expression induced by PRKN (Supplemental Figure 2J), in agreement with the ability of PRKN to cause oxidative stress (30) and the role of ROS in IFN signaling (36).

Based on these data, we next looked for upstream activators of cGAS/STING modulated by PRKN. We found that reexpression of WT PRKN resulted in a 3-to-4–fold increased amplification of mtDNA-encoded ND1 and ND4 genes in cytosolic extracts of PC3 cells (Supplemental Figure 3A, left). Conversely, amplification of nuclear-encoded β2M or TERT mRNA from cytosolic extracts was unchanged (Supplemental Figure 3A, right). The release of mtDNA in cytosol required PRKN E3 ubiquitin ligase activity because expression of a C431S PRKN mutant did not increase ND1 or ND4 gene amplification (Supplemental Figure 3A, left). Similarly, depletion of mtDNA by culture of PC3 cells with ethidium bromide abolished ND1 amplification (Supplemental Figure 3B) but did not significantly reduce PRKN transcriptional upregulation of ISGs, IFIT1, or IFIT2 (Supplemental Figure 3C). In control experiments, parental or mtDNA-depleted PC3 cells comparably responded to LPS stimulation with upregulation of IFNs (Supplemental Figure 3D).

**Figure 3:**
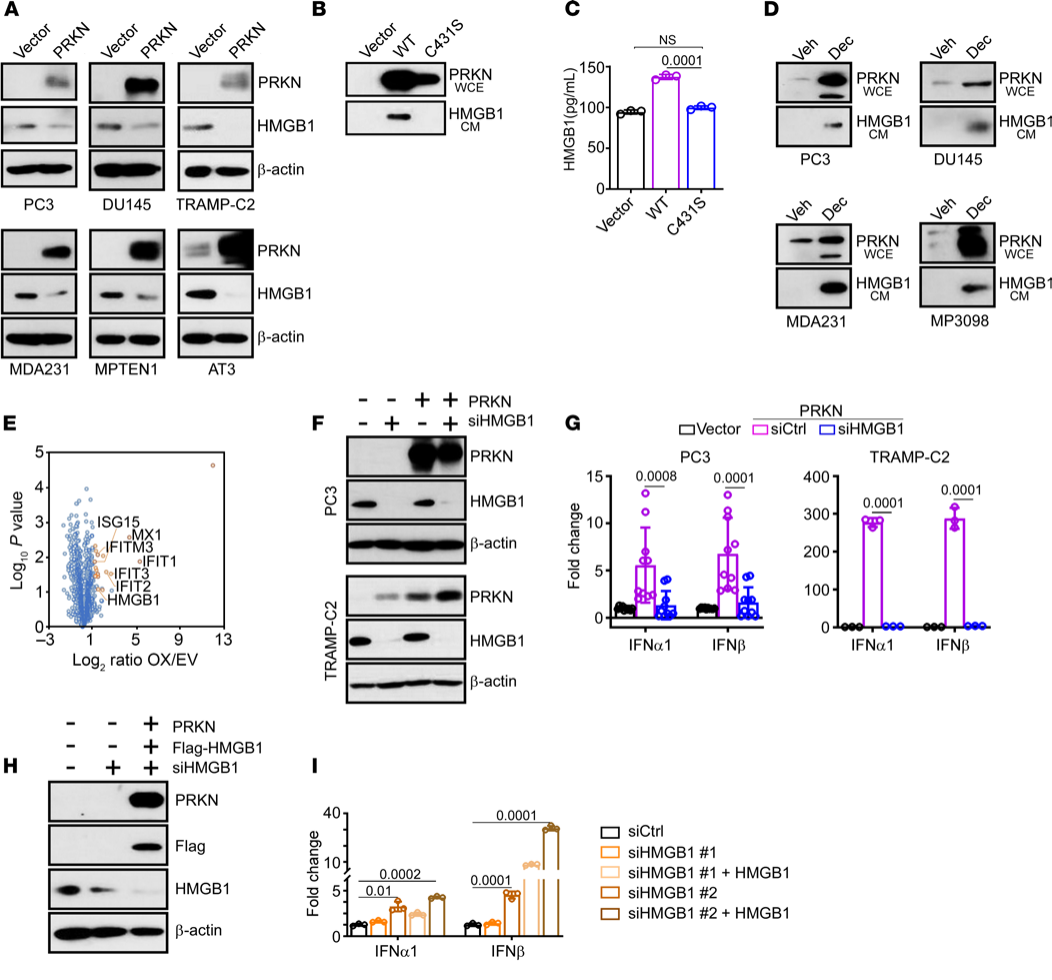
Mechanisms of PRKN regulation of IFN signaling. (**A**) The indicated human (PC3, DU145, MDA231) or murine (TRAMP-C2, MPTEN1, AT3) tumor cell lines expressing vector or PRKN were analyzed by Western blotting. (**B**) Aliquots of whole cell extracts (WCE) or conditioned medium (CM) harvested from PC3 cells expressing WT PRKN or C431S PRKN mutant were analyzed by Western blotting. (**C**) Aliquots of CM from PC3 cells expressing vector, WT PRKN, or PRKN C431S mutant were analyzed by ELISA. Mean ± SD (*n* = 3). (**D**) The indicated tumor cell lines were treated with vehicle (Veh) or decitabine (Dec) and aliquots of WCE or CM were analyzed by Western blotting. (**E**) Aliquots of CM from PRKN-expressing PC3 cells were analyzed by mass spectrometry in a volcano plot. Selected proteins in the PRKN secretome are indicated. OX, PRKN overexpression; EV, empty vector. (**F** and **G**) PRKN-expressing PC3 (top) or TRAMP-C2 (bottom) cells were transfected with control nontargeting siRNA (siCtrl) or HMGB1-directed siRNA (siHMGB1) and analyzed by Western blotting (**F**) or IFN gene expression by RT-qPCR (**G**). Mean ± SD (*n* = 3–5). (**H** and **I**) PC3 cells expressing vector or PRKN were transfected with 2 independent siRNA sequences targeting HMGB1 (siHMGB1 #1, siHMGB1 #2), reconstituted with Flag-HMGB1, and analyzed by Western blotting (**H**) or IFN gene expression by RT-qPCR (**I**). Mean ± SD (*n* = 3). Numbers represent *P* values by 2-tailed unpaired *t* test (**C** and **G**) or 2-way ANOVA (**I**).

Given that the release of mtDNA was insufficient to upregulate PRKN IFN gene expression, we next looked for other activators of cGAS-STING in these settings. We observed that PRKN expression depleted the cellular content of the alarmin and potent DAMP, High Mobility Group Box 1 (HMGB1) (37) in multiple tumor cell types, compared with control cultures (Figure 3A). This response was specific because the levels of extracellular ATP, another DAMP released during cellular damage were unchanged in control or PRKN-expressing PC3 cells (Vector, 1.08 × 10^5^ ± 1.04 × 10^4^; PRKN, 1.02 × 10^5^ ± 0.6 × 10^4^ Relative Luciferase Units, *n* = 4, ns). HMGB1 is a cytokine-like mediator of innate immunity and antiviral responses (38) and was identified in our recent ubiquitylome screen as a high confidence target of PRKN ubiquitination (30). Consistent with a role of PRKN E3 ligase activity in HMGB1 regulation, expression of WT PRKN, but not the PRKN C431S mutant, promoted the release of HMGB1 in the conditioned medium (CM) of PC3 cells, by Western blotting (Figure 3B) and ELISA (Figure 3C). Second, treatment with decitabine, which induces demethylation of the *PARK2* promoter and reexpression of endogenous PRKN in tumor cells (Figure 2, E and F), also induced HMGB1 release in the CM of multiple tumor types (Figure 3D).

To independently validate these findings, we next characterized the secretome released by control or PRKN-expressing cells, by mass spectrometry. We found that reexpression of PRKN in PC3 cells caused the release of multiple ISG, as well as HMGB1 in the CM, compared with vector transfectants (Figure 3E). Mechanistically, siRNA silencing of HMGB1 (Figure 3F) abolished PRKN IFN gene expression in human (PC3) and murine (TRAMP-C2) tumor cell types (Figure 3G). Conversely, reconstitution of HMGB1-silenced PC3 cells with Flag-HMGB1 (Figure 3H) was sufficient to restore the increase in IFN-α and IFN-β mRNA levels induced by PRKN (Figure 3I).

### PRKN paracrine activation of IFN signaling.

**Figure 4:**
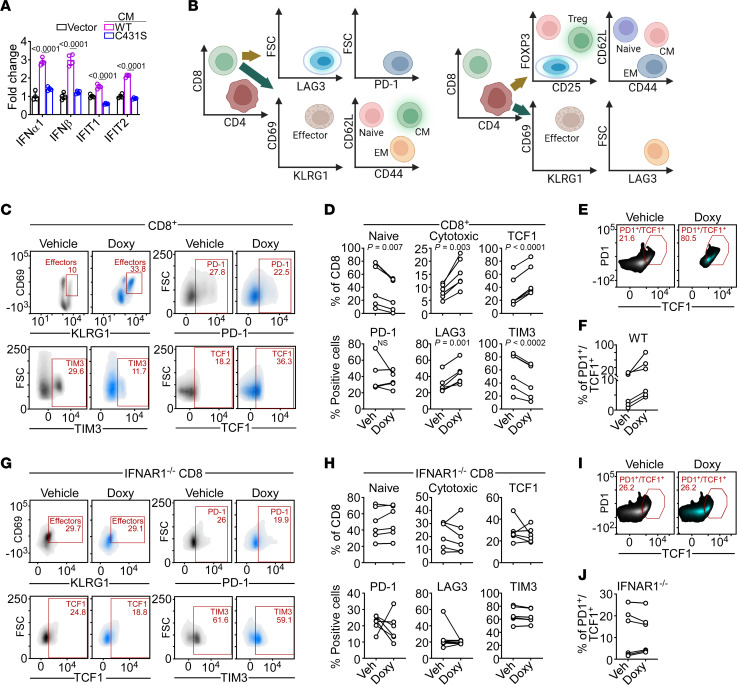
Paracrine CD8 T cell activation by PRKN IFN signaling. (**A**) Recipient PC3 cells were incubated with CM harvested from PC3 cells expressing vector, PRKN, or PRKN C431S mutant and analyzed for IFN gene expression by RT-qPCR. Mean ± SD (*n* = 4). Numbers represent *P* value by 2-way ANOVA. (**B**) Diagram of flow cytometry gating to characterize CD8^+^ (left) or CD4^+^ (right) T cell subsets from CD3^+^/CD19^–^ splenocytes of C57BL/6 mice. Created with BioRender.com. (**C**) CD8^+^ T cells isolated from C57BL/6 splenocytes by negative selection were incubated with CM harvested from PRKN TetON TRAMP-C2 cells in the presence of vehicle or Doxy and analyzed by flow cytometry. Representative plots are shown. The percentage of cells in each quadrant is indicated. (**D**) The conditions are the same as in **C** and PRKN CM modulation of CD8^+^ T cell markers was quantified by flow cytometry in 5 independent experiments. Numbers represent *P* values by 2-tailed unpaired *t* test. (**E** and **F**) The conditions are the same as in **C** and double positive PD-1^+^/TCF1^+^ CD8^+^ T cells were analyzed by flow cytometry in representative density plots (**E**) and results were quantified in 6 independent experiments (**F**). (**G**) CD8^+^ T cells isolated from IFNAR1^–/–^ splenocytes were incubated with CM harvested from PRKN TetON TRAMP-C2 cells as in **C** and analyzed by flow cytometry in representative density plots. The percentage of cells in each quadrant is indicated. (**H**) The conditions are the same as in **G** and modulation of the indicated CD8^+^ T cell markers was quantified in 6 independent experiments. (**I** and **J**) CD8^+^ T cells isolated from IFNAR1^–/–^ splenocytes were analyzed for double-positive PD-1^+^/TCF1^+^ subsets by flow cytometry in representative density plots (**I**) and results were quantified in 6 independent experiments (**J**). The percentage of cells in each quadrant is indicated. Symbols indicate an individual determination.

The data above show that PRKN-expressing cells release a bioactive secretome that contains multiple immune-inflammatory mediators, including HMGB1. Consistent with this model, coincubation of parental PC3 cells with CM harvested from PRKN-expressing cultures was sufficient to strongly increase the expression of IFNs and ISG (Figure 4A). Conversely, the secretome from PC3 cells expressing the PRKN C431S mutant had no effect on IFN gene expression in recipient cells (Figure 4A). Next, we asked whether a PRKN CM could also activate IFN signaling in recipient immune cells. For these experiments, we first optimized a gating strategy of CD3^+^/CD19^–^ splenocytes harvested from C57BL/6J mice to identify CD8 (Figure 4B, left) or CD4 (Figure 4B, right) T cell subsets, by flow cytometry. Second, we engineered syngeneic murine prostate cancer TRAMP-C2 cells to transiently or conditionally (TetON system) express PRKN mRNA and protein (Supplemental Figure 4A). Accordingly, Doxy treatment of stably transduced TRAMP-C2 cells resulted in prominent upregulation of IFN gene expression (Supplemental Figure 4, B and C). Similar results were obtained after decitabine treatment of parental TRAMP-C2 cells, which was associated with increased levels of endogenous PRKN and induction of IFN gene expression (Supplemental Figure 4D).

Under these conditions, coincubation of total splenocytes isolated from C57BL/6J mice with CM from Doxy-treated TRAMP-C2 cells (Supplemental Figure 4B and C) reduced the population of naive CD8^+^ T cells and increased the subset of double-positive KLRG1^+^/CD69^+^ effector cells, compared with control CM (Supplemental Figure 4E and F). This response was accompanied by a unique profile of CD8^+^ T cell activation, characterized by decreased expression of immune inhibitory receptors TIM3 and LAG3, whereas PD-1 levels were not significantly affected (Supplemental Figure 4E and F). Importantly, exposure of C57BL/6J splenocytes to PRKN CM increased the expression of TCF1, a key transcriptional mediator of CD8^+^ T cell stemness and self-renewal properties (Supplemental Figure 4E and F). Conversely, PRKN CM negligibly affected the fraction of central memory CD8^+^ T cells, whereas the effector memory subset was upregulated compared with vehicle-treated CM (Supplemental Figure 4E and F).

As control for these experiments, we exposed C57BL/6J splenocytes to plate-immobilized CD3 and CD28 antibodies to mimic TCR-dependent T cell activation. CD8^+^ T cell activation in these settings also resulted in increased expression of double-positive KLRG1^+^/CD69^+^ as well as TCF1^+^ CD8^+^ T cell subsets with nearly complete disappearance of the naive cell population (Supplemental Figure 4G). However, at variance with the response observed with PRKN CM, CD3/CD28 stimulation prominently upregulated the expression of immune inhibitory receptors LAG3 and PD-1 in C57BL/6J splenocytes, whereas TIM3 levels were mostly unchanged (Supplemental Figure 4G).

To test the specificity of PRKN CM immune modulation, we next purified CD8^+^ T cells from C57BL/6J splenocytes by negative selection. Similar to the results obtained with unfractionated splenocytes, isolated CD8^+^ T cells also responded to incubation with PRKN CM with upregulation of effector/cytotoxic KLRG1^+^/CD69^+^ markers, high TCF1 content, and reduced levels of TIM3 (Figure 4, C and D). PD-1 and LAG3 expression showed limited changes in these settings (Veh, 36.8% ± 7.6%; Doxy, 52.3% ± 6.8%). In addition, coculture with PRKN CM strongly increased the double-positive PD-1^+^/TCF1^+^ CD8^+^ T cell subset (Figure 4, E and F) characterized by effector and self-renewal properties, in vivo.

To identify the requirements of PRKN immune modulation, we next isolated CD8^+^ T cells from splenocytes of mice deficient in type I IFN receptor (IFNAR1^–/–^). Exposure of IFNAR1^–/–^ CD8^+^ T cells to PRKN CM did not affect the expression of KLRG1/CD69, TCF1, or immune inhibitory receptors TIM3 or LAG3 (Figure 4, G and H). Similarly, the subset of double-positive PD-1^+^/TCF1^+^ CD8^+^ T cells from IFNAR1^–/–^ splenocytes was unchanged in the presence of vehicle or PRKN CM (Figure 4, I and J).

Finally, we asked whether PRKN CM immune modulation was selective. Here, coculture of CD4^+^ T cells isolated from C57BL/6J splenocytes with PRKN CM reduced the naive cell population (Veh, 32.8% ± 10.2%; Doxy, 9.2% ± 2.6%), but did not significantly affect KLRG1/CD69 expression (Veh, 22.8% ± 9.2%; Doxy, 29.5% ± 11%) (Supplemental Figure 5, A and B). Different from what observed with CD8^+^ T cells, treatment of CD4^+^ T cells with PRKN CM increased the expression of LAG3 (Veh, 27.5% ± 14.5%; Doxy, 57% ± 20.1%) and PD-1 (Veh, 31.1% ± 12.2%; Doxy, 38.7% ± 14.2%) and lowered the levels of TCF1 (Veh, 12.6% ± 6.3%; Doxy, 6.2% ± 3.5%) (Supplemental Figure 5, A and B). The fraction of immunosuppressive Treg cells was not affected in these settings (Veh, 1.9% ± 1.2%; Doxy, 1.2% ± 0.9%) (Supplemental Figure 5, A and B). As control, activation of CD4^+^ T cells by plate-immobilized antibodies to CD3 plus CD28 lowered the naive cell population while increasing the fraction of effector memory cells, double-positive KLRG1^+^/CD69^+^ effector subset, and the expression of LAG3 and PD-1 compared with controls (Supplemental Figure 5C).

### Deletion of PRKN accelerates transgenic tumor growth.

We next looked at the impact of PRKN immune modulation on tumor growth in vivo. In a first series of experiments, we crossed Transgenic Adenocarcinoma of the Mouse Prostate (TRAMP) mice, which express the SV40 large T antigen oncogene in the prostate under the control of the probasin promoter (39) with PRKN-knockout (PRKN-KO) mice (27). In TRAMP mice, endogenous PRKN is expressed in the prostate at 10 weeks of age, reduced, albeit still detectable, at 26 weeks, and entirely lost by 40 weeks (Supplemental Figure 6A), consistent with the absence of PRKN in advanced tumors and tumor cell lines (30). As control, TRAMP-PRKN–KO mice had no detectable expression of PRKN at 26 weeks of age (Supplemental Figure 6A).

**Figure 5:**
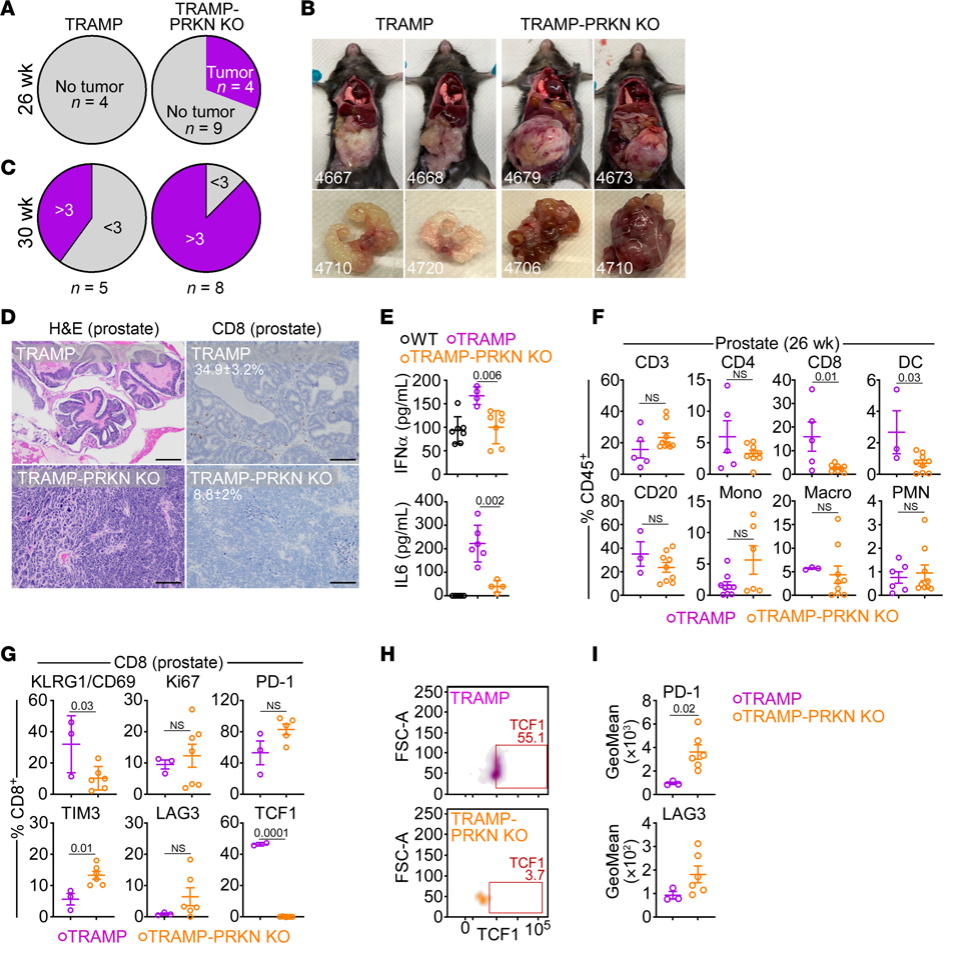
PRKN deletion accelerates prostate tumorigenesis. (**A**) Male TRAMP or TRAMP-PRKN–KO mice were analyzed for prostate tumor formation by IHC at 26 wks of age. (**B**) Representative macroscopic images of prostate tumors formed in TRAMP (29 wks) or TRAMP-PRKN KO (25 wks) mice. (**C**) Tumors harvested from TRAMP or TRAMP-PRKN–KO mice at 30 wks were analyzed for a disease severity (tumor size, hemorrhage, and seminal vesicle invasion; cutoff = 3). For panels **A** and **C**, the number of animals is indicated. (**D**) Prostate tissues from the indicated mouse groups at 26 wks were analyzed by H&E staining (left) or intratumoral accumulation of CD8^+^ T cells, by IHC (right). The percentage of cells is indicated. Representative images. Scale bar: 100 μm. (**E**) Plasma samples from C57BL/6 (WT), TRAMP, or TRAMP-PRKN–KO mice were analyzed for IFN-α (top) or IL6 (bottom) levels, by ELISA. Each point corresponds to an individual determination. (**F**) Prostate tissues from TRAMP or TRAMP-PRKN–KO mice were harvested at 26 wks and analyzed for the indicated immune cell subsets by flow cytometry. DC, dendritic cells; Mono, monocytes; Macro, macrophages. (**G**) The conditions are the same as in **F** and residual intratumoral CD8^+^ T cells were analyzed for expression of the indicated markers by flow cytometry. For all panels, mean ± SD. (**H**) The conditions are the same as in **G** and the percentage of TCF1^+^ CD8^+^ T cells was quantified in TRAMP or TRAMP-PRKN–KO mice (26 wks) by flow cytometry. Representative density plots are shown. (**I**) The conditions are the same as in **G** and geometrical mean fluorescence intensity for PD-1 (top) or LAG3 (bottom) expression in CD8^+^ T cells from TRAMP or TRAMP-PRKN–KO mice is indicated. Mean ± SD. Each point corresponds to an individual determination. Numbers represent *P* values by 2-tailed unpaired *t* test.

The double-transgenic TRAMP-PRKN–KO mice were born viable, fertile, and showed no overt developmental defects. However, loss of PRKN in these mice was associated with early onset prostate cancer formation at 26 weeks of age, when no tumors were detected in TRAMP mice of comparable age (Figure 5A). By 30 weeks of age, tumors grown in TRAMP-PRKN–KO mice were large, often occupying the entire abdominal cavity, intensely hemorrhagic (Figure 5B), and had frequent seminal vesicle invasion. This resulted in a higher disease severity score, which quantifies tumor size, hemorrhage, and local invasion (cutoff, > 3), compared with age-matched TRAMP mice (Figure 5C). Histologically, tumors formed in TRAMP-PRKN–KO mice were comprised of sheets of undifferentiated neuroendocrine-like cells that replaced a prostatic gland architecture still visible in TRAMP mice of comparable age (Figure 5D).

Next, we examined changes in the immune tumor microenvironment of TRAMP versus TRAMP-PRKN–KO mice. At 26 weeks of age, TRAMP-PRKN–KO mice exhibited reduced intratumoral accumulation of CD8^+^ T cells (Figure 5D) and lower plasma levels of IFN-α and IL6 compared with TRAMP mice (Figure 5E). Consistent with these data, prostate tumors harvested from TRAMP-PRKN–KO mice at 26 weeks showed severe depletion of CD8^+^ T cells as well as dendritic cells (DCs) compared with TRAMP mice, by flow cytometry (Figure 5F). In contrast, intratumoral B cells or various myeloid subsets were not significantly affected, and a reduction in CD4^+^ T cells did not reach statistical significance (Figure 5F).

We next collected residual intratumoral CD8^+^ T cells from TRAMP or TRAMP-PRKN–KO mice and characterized their immune profile by multiparametric flow cytometry. Intratumoral CD8^+^ T cells in TRAMP-PRKN–KO mice showed severe depletion of KLRG1/CD69, complete loss of TCF1 (Figure 5, G and H), and a trend toward increased expression of the immune inhibitory receptors PD-1, TIM3, and LAG 3 (Figure 5G). Quantification of fluorescence intensity confirmed the upregulation of PD-1 and LAG3 in intratumoral CD8^+^ T cells from TRAMP-PRKN–KO mice compared with TRAMP mice (Figure 5I). Conversely, intratumoral CD4^+^ T cells from TRAMP-PRKN–KO mice showed no significant changes in expression of Ki67 or immune inhibitory receptors with only a modest increase in the population of double positive KLRG1^+^/CD69^+^ effector cells and complete loss of TCF1 expression (Supplemental Figure 6B).

Finally, we asked whether the pathway of PRKN immune modulation was restricted to the tumor microenvironment. Here, flow cytofluorometric analysis of spleens (Supplemental Figure 6C) and pelvic lymph nodes (Supplemental Figure 6D) harvested from TRAMP or TRAMP-PRKN–KO mice at 26 weeks of age showed no significant differences in lymphoid or myeloid subsets (Supplemental Figure 6, C and D), except for a reduction in DCs and accumulation of PMN in spleens of TRAMP-PRKN–KO mice (Supplemental Figure 6, C and D). In addition, PRKN immune modulation was specific for tumor-bearing mice because PRKN-KO mice showed no significant changes in lymphoid or myeloid splenocytes at 26 weeks of age compared with WT C57BL/6J mice (Supplemental Figure 6E).

### PRKN regulation of antitumor immunity.

To independently complement the results obtained with genetically engineered mice and reinforce the generality of PRKN antitumor immunity, we next established a syngeneic mammary gland tumor model. For these experiments, murine breast adenocarcinoma AT3 cells were engineered to conditionally express PRKN (TetON system) in response to Doxy (Supplemental Figure 7A, inset). Treatment of these cells with Doxy potently induced an IFN gene signature (Supplemental Figure 7A), whereas decitabine treatment of parental AT3 cells increased PRKN and IFN gene expression compared with vehicle (Supplemental Figure 7B).

**Figure 6:**
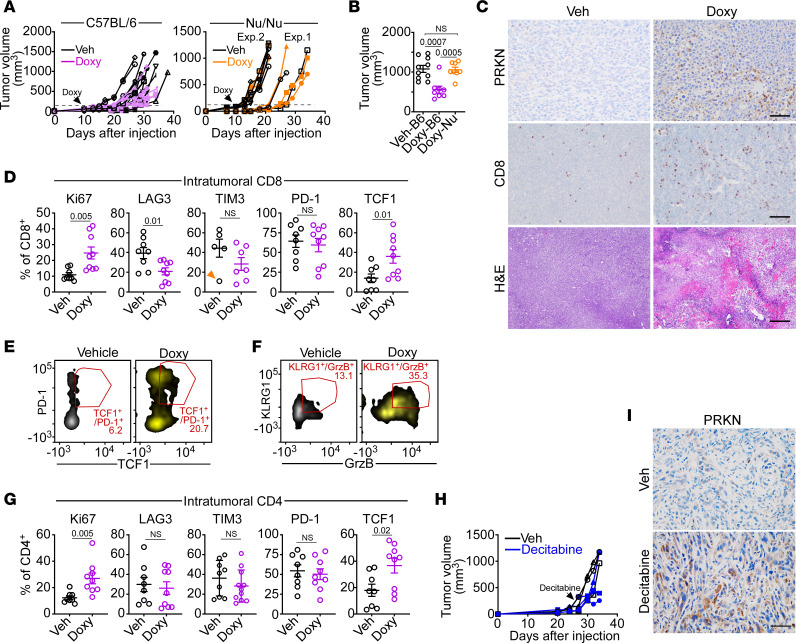
PRKN antitumor immunity. (**A**) C57BL/6 (left) or nude Nu/Nu (right) mice were engrafted with syngeneic PRKN TetON AT3 cells in the mammary fat pad, and tumor growth was quantified with a caliper. Doxy (500 ng/mL) or vehicle (Veh) was administered in the drinking water (arrow) when tumors reached a volume of approximately 120–150 mm^3^. Each line is an individual tumor. Two independent experiments (Exp) with Nu/Nu mice are shown. (**B**) AT3 tumors (as in **A**) were quantified with a caliper at the end of the experiment. Veh, vehicle; B6, C57BL/6; Nu (Nu/Nu) mice. Mean **±** SD. Veh-B6 (*n* = 10), Doxy-B6 (*n* = 8), Doxy-Nu (*n* = 8). (**C**) AT3 tumors grown in C57BL/6 mice in the presence of vehicle (Veh) or Doxy were analyzed by IHC or H&E staining. Scale bar: 50 μm. (**D**) CD8^+^ T cells harvested from PRKN TetON AT3 tumors in vehicle (Veh)- or Doxy-treated C57BL/6 mice were analyzed for the indicated markers by flow cytometry. Arrow indicates an outlier in TIM3 reactivity. (**E** and **F**). Intratumoral CD8^+^ T cells (as in **D**) were analyzed for double-positive PD1^+^/TCF1^+^ (**E**) or KLRG1^+^/GrzB^+^ (**F**) subsets by flow cytometry. Representative density plots are shown. The percentage of double-positive cells is indicated. For all panels, each point corresponds to an individual determination. (**G**) CD4^+^ T cells harvested from PRKN TetON AT3 tumors in vehicle (Veh)- or Doxy-treated C57BL/6 mice were analyzed for the indicated markers by flow cytometry. (**H**) C57BL/6 mice engrafted with syngeneic flank TRAMP-C2 tumors were administered vehicle (Veh) or decitabine (2.5 mg/kg daily) once tumors reached approximately 150 mm^3^ (arrow) and tumor growth was quantified with a caliper. (**I**) TRAMP-C2 tumors harvested from the indicated mouse group were analyzed by IHC. Representative images are shown. Scale bar: 50 μm. Numbers represent *P* values by 2-tailed unpaired *t* test.

Injection of TetON PRKN AT3 cells in the mammary fat pad of syngeneic immunocompetent C57BL/6J mice gave rise to exponentially growing orthotopic mammary gland tumors (Figure 6A, left and Figure 6B). Addition of Doxy to the drinking water of tumor-bearing mice induced high levels of intratumoral PRKN expression, determined by IHC (Figure 6C), accompanied by significant inhibition of tumor growth (Figure 6A, left and Figure 6B). Histologically, AT3 tumors in Doxy-treated animals showed increased accumulation of CD8^+^ T cells and collapse of tumor architecture with extensive tissue necrosis and hemorrhage (Figure 6C). Doxy, but not vehicle-treated animals, also show intense intratumoral staining for HMGB1 (Supplemental Figure 7C, left). To test whether this antitumor response required an intact immune system, we next engrafted AT3 mammary gland tumors in immunocompromised nude Nu/Nu mice. Here, conditional expression of PRKN by addition of Doxy to the drinking water had no effect on AT3 mammary tumor growth (Figure 6A, right and Figure 6B). Consistent with Doxy-induced PRKN expression, these tumors maintained high content of HMGB1, determined by IHC (Supplemental Figure 7C, right).

With respect to an immune microenvironment, AT3 tumors harvested from Doxy-treated C57BL/6J mice showed strong accumulation of CD8^+^ as well as CD4^+^ T cells by both analysis of CD45^+^-gated (Supplemental Figure 7D) and live cell (Supplemental Figure 7E) populations. These changes were specific because B cells (CD20^+^), NK, DCs, or Treg cells (Supplemental Figure 8A) or myeloid subsets (Supplemental Figure 8B) were unchanged. Mirroring the phenotype observed in PRKN-KO mice, intratumoral CD8^+^ T cells harvested from Doxy-induced mice expressed high levels of the proliferation marker Ki67 as well as TCF1, whereas LAG3 and TIM3 were reduced, and PD-1 remained at intermediate levels (Figure 6D). Conditional PRKN expression in these mice also upregulated double-positive TCF1^+^/PD-1^+^ (Figure 6E and Supplemental Figure 8C) and KLRG1^+^/GrzB^+^ (Figure 6F and Supplemental Figure 8D) subsets, implicated in T cell renewal and antitumor cytolytic activity, respectively. Intratumoral CD4^+^ T cells from the same mice showed increased expression of Ki67 and TCF1, but no significant changes in immune inhibitory receptors (Figure 6G). Similar to the results obtained with TRAMP-PRKN–KO mice, PRKN immune modulation in the AT3 model was restricted to the tumor microenvironment, as splenocytes harvested from control or Doxy-treated animals showed no significant differences in the expression of CD8^+^ (Supplemental Figure 8E) or CD4^+^(Supplemental Figure 8F) T cell markers.

Finally, we asked whether intratumoral reexpression of endogenous PRKN could be achieved by demethylating therapy in vivo. For these experiments, we engrafted prostate cancer TRAMP-C2 cells onto the flanks of C57BL/6J mice and treated the animals with decitabine, which restores PRKN expression and associated IFN gene expression (Supplemental Figure 4D). Here, systemic administration of decitabine inhibited TRAMP-C2 tumorigenesis (Figure 6H) and significantly reduced maximal tumor growth compared with vehicle-treated animals (vehicle, 0.89 ± 0.07 g; decitabine, 0.42 ± 0.11 g, mean ± SD, *P* = 0.01). In addition, animals treated with decitabine showed strong intratumoral expression of PRKN, whereas vehicle had no effect, determined by IHC (Figure 6I).

## Discussion

In this study, we have shown that reexpression of PRKN in genetically disparate tumor types transcriptionally activates a potent IFN response. Mechanistically, this pathway required PRKN E3 ligase activity, involved the subcellular trafficking and release of the alarmin DAMP HMGB1, and activated the cGAS/STING complex in the cytosol, while inhibiting NF-κB–dependent gene expression. As a result, PRKN-expressing cells released an immune-inflammatory secretome rich in IFNs, ISG, and pleiotropic cytokines that stimulated IFNAR1-dependent paracrine activation of effector and cytotoxic CD8^+^ T cells, promoting their intratumoral accumulation and the inhibition of transgenic and syngeneic tumor growth in vivo. Importantly, PRKN antitumor immunity was therapeutically actionable, and a clinically approved demethylating therapy with decitabine restored epigenetically silenced endogenous PRKN expression in cancer and enabled HMGB1-dependent IFN gene expression.

Despite extensive efforts, the role of PRKN in human disease continues to remain elusive. According to a prevailing model, disruption of a PRKN-PINK1 mitophagy axis allows the persistence of harmful mitochondria that poison dopaminergic neurons (40), contributing to the pathogenesis of PD (24, 25). An inhibitory role of PRKN on innate immunity proposed in earlier studies (26–28) may further compound this scenario and heighten neuroinflammation, which is another hallmark of PD pathogenesis (29). At variance with this model, we found that reexpression of PRKN in cancer does not cause mitophagy or other hallmarks of mitochondrial dysfunction (30) and potently activates, rather than inhibits, innate immunity through DAMP-regulated IFN signaling. Whether these findings can be extended beyond tumor responses and influence the pathogenesis of PD, where the *PARK2* gene is mono or biallelically altered (24, 25), is presently unknown. However, it should be noted that defective innate immunity and failure to achieve pathogen clearance are also important drivers of neuroinflammation and PD pathogenesis (41). Accordingly, PRKN and PINK1-KO mice show diminished antiviral responses (42), impaired T cell functions (43), and defective DC-mediated antigen presentation (44), reinforcing the mechanistic model of PRKN immune modulation presented here.

How PRKN E3 ubiquitin ligase activity participates in innate immunity and IFN signaling remains to be determined. There is abundant precedent for different classes of E3 ubiquitin ligases regulating IFN signaling (45) and enabling tumor suppression (46). Here, PRKN-induced release of mtDNA in the cytosol, a potent DAMP and major cGAS/STING activator (47), was insufficient to recapitulate an IFN response in cancer. Conversely, PRKN-induced E3 ligase-dependent release of HMGB1 (48) were required for IFN gene expression. HMGB1 was identified in our PRKN ubiquitylome screen as a high-confidence substrate of PRKN ubiquitination in cancer (30), and it is known that multiple posttranslational modifications are involved in subcellular trafficking of HMGB1 (48).

In this context, a possibility is that PRKN ubiquitination contributes to the shuttling of HMGB1 between its various subcellular compartments and eventual extracellular release. As a multifunctional, cytokine-like alarmin (48), HMGB1 has contextual effects in cancer, favoring tumorigenesis via sustained inflammation (37), or, as proposed here, contributing to anticancer responses (49) through immunogenic cell death and heightened IFN signaling (50). We have shown that conditional PRKN expression results in prominent release of HMGB1 in the tumor microenvironment in vivo, and this pathway, combined with a limited release of mtDNA without overt mitochondrial damage (30), may converge to enhance STING- and IRF3-dependent IFN gene expression for PRKN tumor suppression.

Importantly, PRKN stimulation of IFN signaling was not limited to overexpression approaches but could be reproduced by restoring endogenous PRKN levels through clinically approved demethylating therapy. Although undetectable in most malignancies (30), consistent with a general role in tumor suppression (31), the mechanism(s) of deregulated PRKN expression in cancer have not been clearly delineated. Our observation that the *PARK2* promoter is heavily methylated in certain tumors in vivo, correlating with worse patient outcome, points to epigenetic silencing as one of the mechanisms for PRKN loss in cancer, compounding other examples of genetic inactivation (51). Overall, these findings fit well with other data that deregulated methylation of the *PARK2* promoter correlates with PRKN loss in acute leukemia (52) and is associated with poor survival in patients with advanced breast cancer (53).

Although an IFN response in cancer is contextual (22) and may trigger pro-or antitumorigenic responses, PRKN IFN signaling delivered potent anticancer activity, inhibiting transgenic and syngeneic tumor growth in vivo. This pathway involved predominantly CD8^+^ T cells, and, to a lesser extent, CD4^+^ T cells, but no other lymphoid or myeloid populations, was restricted to the tumor microenvironment and required IFNAR1 recognition. Consistent with this, PRKN inhibition of tumor growth depended on a competent immune system and was abolished in immunocompromised Nu/Nu mice. At variance with other models of mitochondria-associated immune responses (54), PRKN profoundly suppressed NF-κB–dependent inflammation, a nearly universal protumorigenic mechanism (55), and downregulated the expression and phosphorylation of oncogenic STAT molecules, especially STAT3 (56). While the molecular basis of these responses remains to be elucidated, it is intriguing that PRKN potently activated STAT1, not only a key requirement of IFN gene expression (10), but also as a direct inhibitor of NF-κB (57) and effector of antitumor immunity (58).

A key feature of PRKN antitumor immunity was a unique profile of paracrine CD8^+^ T cell activation. Analysis of intratumoral CD8^+^ T cells in the presence of PRKN revealed heightened expression of effector and cytotoxic markers, lower levels of immune inhibitory receptors TIM3 (59) and LAG3 (60), intermediate expression of PD-1, and high content of the progenitor/stem cell factor, TCF1 (61). These are all molecules that control the balance between T cell exhaustion (62) and T cell self renewal (61), and their expression is a major determinant of how patients may respond to therapeutic ICI in the clinic (63, 64). More work is required to determine how PRKN may affect the continuum of T cell exhaustion (62), from exhausted effector T cells retaining antitumor activity to terminally exhausted T cells, which are devoid of effector functions (65). However, the effector/cytotoxic (KLRG1^+^/CD69^+^/GrzB^+^) and proliferative (Ki67^+^) CD8^+^ T cell phenotype induced by PRKN combined with LAG3^lo^, TIM3^lo^, PD-1^intermediate^ and TCF1^hi^ expression has been associated with reinvigorated T cell functions, more durable antitumor activity, and better responses to therapeutic ICI in patients (63, 64). Together with other findings that PRKN is required for efficient tumor antigen presentation and response to immunotherapy (44), these data identify PRKN as a therapeutically actionable, multifunctional mediator of antitumor immunity.

On the other hand, it is clear that other mechanisms also participate in PRKN tumor suppression. In fact, several independent studies have highlighted the ability of PRKN to inhibit multiple intrinsic tumor traits of metabolic reprogramming (30, 66, 67), mitotic transitions (32), and cell motility and invasion (34), all independent of mitophagy. The role of PRKN in reinvigorating an antitumor immune microenvironment via IFN signaling and effector/cytotoxic CD8^+^ T cell activation (this study) adds to these pathways and defines a unique pathway of dual mode tumor suppression. Dual mode tumor suppressors are rare, or at least have not been readily described. To the best of our knowledge, only p53 (68) and PTEN (69) have been reported to target intrinsic tumor traits while also activating cGAS/STING and IRF3, respectively, for antitumor immunity. However, different from these molecules, which are genetically lost in cancer, PRKN epigenetic silencing in tumors (52, 53) is reversible by clinically approved demethylating therapy (70), which restores endogenous PRKN levels in the tumor microenvironment and associated IFN signaling. On this basis, epigenetic therapy may be a suitable approach to reawaken PRKN dual tumor suppression in the clinic (30), reinvigorating effector T cell functions in the microenvironment (this study) and tumor antigen presentation (44), while concomitantly inhibiting tumor cell metabolism and invasion (30, 34, 66, 67).

### Conclusions.

The data presented here identify PRKN, a molecule known for its association with Parkinson’s Disease, as a mediator of antitumor immunity. This pathway exploits an ancient machinery of innate immunity that protects against pathogens to reinvigorate CD8^+^ T cell functions in the tumor microenvironment and is therapeutically actionable by a clinically approved demethylating agent. A role of PRKN in immune modulation and enhanced T cell effector functions carries important implications for the pathogenesis of cancer and other conditions, including the response to infectious pathogens and Parkinson’s Disease.

## Methods

### Sex as a biological variable.

This study involved the use of laboratory animals to recapitulate human tumor models. Male mice were used for preclinical models of transgenic and syngeneic prostate cancer. Female mice were used for syngeneic models of mammary gland adenocarcinoma.

Additional methods are available in the Supplemental Methods.

### Gene expression analysis.

PC3 cells transiently transfected with PRKN were analyzed by RNA-Seq and data were aligned using the bowtie2 (71) algorithm against hg19 human genome version. The RSEM v1.2.12 software (72) was used to estimate read counts and RPKM values using gene information from Ensemble transcriptome version GRCh37.p13. Raw counts were used to estimate the significance of differential expression between 2 experimental groups using DESeq2 (73). Overall gene expression changes were considered significant if they passed FDR < 5% threshold. Gene set enrichment analysis was done using QIAGEN’s Ingenuity Pathway Analysis software (IPA, QIAGEN) using the “canonical pathways” option. Pathways that passed significance of the FDR < 5% threshold and had significantly predicted activation state (|Z-score| > 2) were reported.

### Protein analysis.

The various cell types were lysed in RIPA buffer containing phosphatase inhibitors (Roche). Protein concentrations were determined with a Bradford assay (Biorad), and 40 μg of proteins were loaded on 10% NuPAGE polyacrylamide gels (Thermo Fisher Scientific). After separation by electrophoresis, proteins were transferred onto a PVDF membrane using a wet system with 1 × transfer buffer (Thermo Fisher Scientific). Membranes were blocked in 5% milk for 2 hours at 22°C and incubated with primary antibodies of various specificities (complete list of antibodies in Supplemental Methods) in PBS plus 1% BSA (Sigma-Aldrich) for 16 hours at 4°C. Membranes were washed in TBS and incubated with ECL anti-rabbit HRP-conjugated secondary reagent (NA934V, Amersham) (1:100) in 5% milk for additional 2 hours at 22°C. Protein bands were visualized using Clarity Western ECL Substrate (Biorad) using Amersham Hyperfilm ECL film and a Konica SRX-101A Developer. To characterize a PRKN-induced secretome, TetON PRKN TRAMP-C2 cells were treated with Doxy or vehicle (DMSO) for 48 hours and aliquots of serum-free CM were concentrated using Amicon Ultra-4 10 kDa centrifugal filter units (Sigma-Aldrich). Concentrated proteins were in-gel digested and analyzed using a 2.5 hour LC gradient on a Thermo Q Exactive HF mass spectrometer. In some experiments, PRKN TetON TRAMP-C2 cells were incubated with vehicle (DMSO) or Doxy for 4 days at 37°C, harvested, and seeded at 5 × 10^5^ cells/mL in medium containing 10% FBS without DMSO or Doxy for 16 hours at 37°C. On the day of the experiment, aliquots of CM were harvested from the various cell types, centrifuged to remove cell debris, and processed for further immune activation studies.

### RT-qPCR.

Total cell lysates were prepared in RNA lysis buffer from Quick-RNA Microprep kit (Zymo Research) and stored at –80°C. RNA was extracted using a Quick-RNA Microprep kit following the manufacturer’s instructions. cDNA was obtained with High-Capacity cDNA Reverse Transcription Kit in the presence of RNase inhibitor (Thermo Fisher Scientific) using a Bio-Rad thermocycler. qRT-PCR amplification reactions were performed using primers for the gene of interest and Applied Biosystems PowerUp SYBR Green Master Mix (Thermo Fisher Scientific). The primer sequences used for RT-qPCR amplification experiments in this study are shown in Supplemental Table 1.

### Flow cytometry.

Spleens were isolated from TRAMP, IFNAR1^–/–^, PRKN^–/–^, TRAMP-PRKN–KO, or WT C57BL/6J mice under the various tumor conditions tested. Samples were mechanically dissociated, filtered through a 70 μm filter (VWR International) and red blood cells (RBC) were lysed using Ammonium-ChloridePotassium (ACK) Lysing Buffer (Lonza Biosciences). Mammary fat pad tumors (AT3), superficial flank tumors (TRAMP-C2), or prostate (TRAMP mice) samples harvested from the various animal cohorts were mechanically dissociated into single cell suspensions using a Tumor Dissociation kit (Miltenyi Biotec), cells were filtered, and RBC were lysed in ACK buffer. For staining of surface markers, cells were incubated with aliquots of antibody mix (50 μL total/sample) in Brilliant Stain Buffer (Becton Dickinson) for 15 minutes at 4°C, washed in MACS buffer containing 1% FBS and 5 mM EDTA in PBS and stained with eBioscience Foxp3/Transcription Factor Staining Buffer Set (Thermo Fisher Scientific) for 45 minutes at 4°C with gentle agitation on an orbital shaker. Aqua or Zombie UV LIVE/DEAD Viability/Cytotoxicity Kit was added (1:300) to the surface antibody mix. For flow cytometry analysis, compensation was set with UltraComp eBeads Compensation Bead (for use with antibodies) and ArC Amine Reactive Compensation Bead Kit (for use with LIVE/DEAD; Fixable dead cell stain kits). Samples were acquired on a Becton Dickinson FACSymphony Cell Analyzer and data were analyzed with FlowJo 10.7 software (Tri-Star). The antibodies used for the characterization of PRKN modulation of immune cell subsets are shown in Supplemental Table 2. For identification of individual CD45^+^ immune cell subsets, the following combinations of markers were used: PMN, CD11b^+^/LY6G^hi^/LY6C^–/lo^; monocytes, CD11b^+^/LY6G^lo^/LY6C^hi^; macrophages (spleen), F4/80^+^/CD11b^–^; macrophages (tumor), F4/80^+^/CD11b^+/lo^; DC, F4/80^–^/CD11b^+^/CD11c^+^/MHC Class II^hi^; NK, CD19^–^/CD3^–^/Nk1.1^+^.

### T cell activation.

Single-cell suspensions of unfractionated splenocytes (3 × 10^5^) harvested from C57BL/6J mice were seeded onto 96-well U-bottom plates (200 μL) in medium containing 10% FBS. Cells were mixed with control medium or CM from PRKN TetON TRAMP-C2 cells. In some experiments, CD8^+^ or CD4^+^ T cell subsets were isolated from mouse splenocytes (C57BL/6J and IFNAR1^–/–^ mice) by negative selection using a CD8a^+^ or CD4^+^ mouse T cell isolation kit (Miltenyi), respectively, following the manufacturer’s specifications and plated at 2 × 10^5^ cells/well in a final volume of 200 μL. After 24 hours, cells were stained with the target antibody panel and analyzed by flow cytometry. As positive control for T cell activation in vitro, 96-well plates were coated with 1 μg/well of Ultra-LEAF purified anti-mouse CD3 antibody plus 0.5 μg/well of Ultra-LEAF purified anti-mouse CD28 antibody (both from Biolegend) for 16 hours at 4°C. After incubation, the antibodies were removed and wells were washed twice with PBS, pH 7.4, before addition of splenocytes or isolated CD8^+^ or CD4^+^ T cells. T cells seeded in uncoated wells were used as negative control.

### PARK2 gene methylation.

Pan-cancer DNA methylation data (Methylation450K) from TCGA dataset were downloaded through Xena browser (74). From this data, Illumina EPIC-8v2 probes of potential *PARK2* promoter sites were extracted to evaluate methylation in the *PARK2* promoter. To examine hyper and hypo methylated *PARK2* promoter probes, TCGA cancer types with matched tumor and adjacent healthy methylation data were utilized. For each cancer type, methylation β values were compared in pairs of tumor and adjacent healthy samples using paired sample rank-sum test (through Python scipy.stats.ranksums). The log-transformed *P* values (for *P* < 0.01) were visualized via clustermap (through Python seaborn.clustermap). A subset of hypermethylated *PARK2* promoter probes were visualized in matched tumor-healthy samples using boxplots (through Python seaborn.boxplot). The influence of *PARK2* promoter methylation over patient outcomes was evaluated by comparing disease supplemental table survival (DSS) (75) in patients with high versus low *PARK2* methylation in the cg14584255 probe. cg14584255 resides in *PARK2* enhancer region, nearest the genomic region that was found to be hypermethylated in DU145 and MDA231 cell types through CCLE RRBS dataset, obtained through the DepMap portal (76). Separating patients into high versus low cg14584255 methylation by the median β value, patient DSS was compared using a log-rank test (through Python lifelines.statistics.logrank_test).

### Animal studies.

In a first series of experiments, longitudinal cohorts of TRAMP mice or TRAMP-PRKN–KO double transgenic mice were harvested at 26 or 30 weeks (wks) of age. Prostate tissue, spleens, and pelvic lymph nodes from the various animal groups were collected and processed for IHC and flow cytometry. A disease severity score (cutoff = 3) based on tumor size (range: 0–5) and histological evidence of hemorrhage (range: 1–5) and seminal vesicle invasion was used to quantify prostate cancer presentation in TRAMP or TRAMP-PRKN–KO mice at 30 wks of age. Second, cohorts of WT C57BL/6J mice or, alternatively, immunocompromised Nu/Nu (nude) mice were injected (5 × 10^4^ cells/mouse) with growth factor–free Matrigel at 1:2 ratio in the mammary fat pad with PRKN TetON AT3 cells. When mammary gland tumors reached a volume of 120–150 mm^3^, groups of animals were administered Doxy (500 ng/mL) in the drinking water and tumor burden was quantified with a caliper at increasing time intervals. At the end of the experiment, orthotopic mammary gland tumors in the various animal groups were harvested, processed for IHC, and single-cell suspensions were analyzed by flow cytometry. For decitabine treatment, in vivo TRAMP-C2 cells were engrafted (5 × 10^6^ cells/mouse) in the flank of immunocompetent C57BL/6J mice in a 1:1 ratio with matrigel. When tumors reached an average volume of approximately 150 mm^3^, animals were administered decitabine (2.5 mg/Kg) daily for 2 wk by i.p. injection. Tumors were harvested at the end of the experiment and analyzed for differential expression of endogenous PRKN by IHC.

### Statistics.

Data are expressed as mean ± SD of results from a minimum of 3 independent experiments. Unpaired, 2-tailed Student’s *t* tests were used for 2-group comparative analyses. In some cases, correction for multiple testing by the Benjamini-Hochberg procedure was obtained. For multiple-group comparisons, 2-way ANOVA with option of multiple comparison was used. All statistical analyses were performed using a GraphPad software package (Prism 10) for Windows. A *P* value less than 0.05 was considered statistically significant.

### Study approval.

All experiments with laboratory animals were carried out in accordance with the recommendations in the Guide for the Care and Use of Laboratory Animals of the National Institutes of Health (NIH). Protocols were approved by the Institutional Animal Care and Use Committee (IACUC) of The Wistar Institute. Sample size was determined by power analysis. All animals were included in the analysis.

### Data availability.

The datasets reported in the current study are available from the corresponding authors upon reasonable request. Supporting data values are available with this manuscript.

## Author contributions

MP and DCA conceived the project. MP performed experiments of PRKN immune modulation and inhibition of syngeneic tumor growth; MP, EA, and ATM characterized PRKN regulation of IFN response, NF-κB activation, and TRAMP-PRKN–KO tumorigenesis; IB analyzed mitochondrial DNA release; MY characterized PRKN regulation of HMGB1 trafficking and IFN response; MR generated genetically engineered prostate cancer cell lines used in this study; JCG examined PRKN-induced cytokine release; CC contributed to the characterization of PRKN immune phenotyping; HYT analyzed a PRKN-induced *secretome* by mass spectrometry; NA identified differential patterns of *PARK2* methylation in cancer; IT and SPA carried out experiments of methylation-specific PCR, AVK conducted bioinformatics analysis; NG provided IFIT1 promoter reporter constructs; MP, EA, NA, and DCA analyzed data, and MP and DCA wrote the paper.

## Supplementary Material

Supplemental data

Unedited blot and gel images

Supporting data values

## Version 1 08/30/2024

In-Press Preview

## Version 2 11/15/2024

Electronic publication

## Version 3 01/03/2025

Updated fig 3A and supplemental gel file to fix errors
