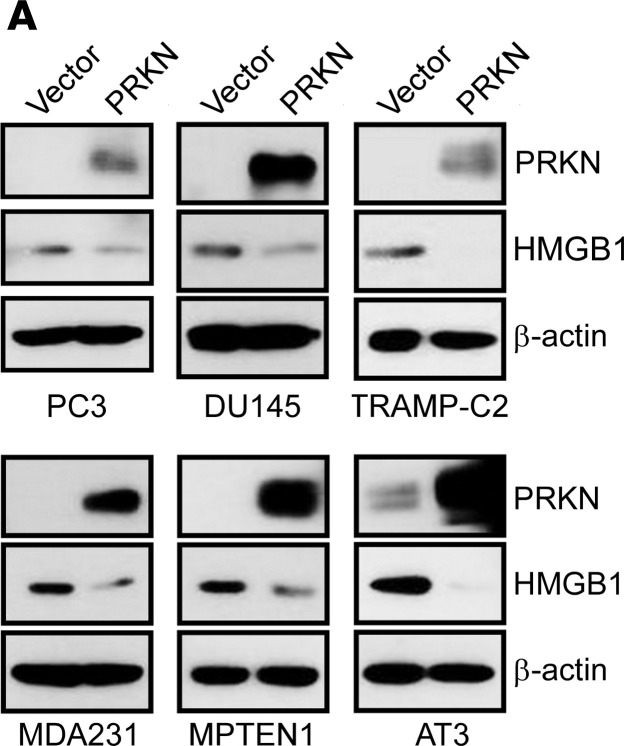# Parkin activates innate immunity and promotes antitumor immune responses

**DOI:** 10.1172/JCI190291

**Published:** 2025-01-16

**Authors:** Michela Perego, Minjeong Yeon, Ekta Agarwal, Andrew T. Milcarek, Irene Bertolini, Chiara Camisaschi, Jagadish C. Ghosh, Hsin-Yao Tang, Nathalie Grandvaux, Marcus Ruscetti, Andrew V. Kossenkov, Sarah Preston-Alp, Italo Tempera, Noam Auslander, Dario C. Altieri

Original citation: *J Clin Invest*. 2024;134(22):e180983. https://doi.org/10.1172/JCI180983

Citation for this corrigendum: *J Clin Invest*. 2025;135(2):e190291. https://doi.org/10.1172/JCI190291

Following the publication of this article, a reader noted that the Figure 1F and Figure 3A PC3 panel showed the same PRKN and β-actin blots. The authors have indicated that Figure 3A was incorrectly assembled and have provided the corrected panel below. In addition, the authors have provided an updated version of the unedited blot document to include the correct files. The HTML and PDF files have been updated to reflect this change.

The authors regret the error.

## Supplementary Material

Unedited blot and gel images

## Figures and Tables

**Figure 3A:**