# Supplementary material for: Parkin activates innate immunity and promotes antitumor immune responses
Source: J Clin Invest. 2024 Aug 30;134(22):e180983. doi: 10.1172/JCI180983 (PMC11563675; doi:10.1172/JCI180983)
Supplement: Supplemental data [file jci-134-180983-s007.pdf]

Rev. Ms. 180983-JCI-RG-3

SUPPLEMENTAL MATERIAL

PARKIN ACTIVATES INNATE IMMUNITY AND PROMOTES ANTI-TUMOR IMMUNE RESPONSES

Michela Perego, Minjeong Yeon, Ekta Agarwal, Andrew T. Milcarek, Irene Bertolini, Chiara Camisaschi, Jagadish C. Ghosh, Hsin-Yao Tang, Nathalie Grandvaux, Marcus Ruscetti, Andrew V. Kossenkov, Sara Preston-Alp, Italo Tempera, Noam Auslander and Dario C. Altieri

Table of Contents

Supplemental Methods

Supplemental Figs. S1 – S8

Supplemental references

Supplemental Table S1 – S2

SUPPLEMENTAL METHODS

Antibodies and reagents. The following antibodies to Parkin (PRKN) (#2132S), vinculin (#4650S and #13901), STING (#13647S), phosphorylated (p) STING (Ser366; #50907S), pIRF3 (Ser396; #29047S), IRF3 (#11904S), pSTAT1 (Tyr701; #8826S), STAT1 (#14994S), pSTAT3 (Tyr705; #9145S), STAT3 (#12640S), and HMGB1 (#6893S) were purchased from Cell Signaling. An antibody to dsDNA was purchased from Abcam (#ab273137). A rabbit polyclonal antibody to PRKN (#14060-1-AP) used for immunohistochemistry was from Proteintech. A rabbit anti-mouse CD8α (D4W2Z) antibody XP® (#98941) used for immunohistochemistry was from Cell Signaling. Secondary HRP horse anti-rabbit IgG polymer reagent (Vector Laboratories) was used for immunohistochemistry. An antibody to β-actin was from Sigma. Secondary antibodies for flow cytometry studies were from Molecular Probes. siRNA sequences to silence HMGB1 (5'-rCrUrGrArUrArArArArGrGrUrUrUrUrGrUrCrArArArCrATT-3' — 5'-rArArUrGrUrUrUrGrArCrArArArArCrCrUrUrUrUrArUrCrArGrCrC-3') or STING were obtained from Integrated DNA Technologies (IDT). Two independent siRNA sequences targeting PRKN (J-003603-05, PARK2) were obtained from Dharmacon and characterized in previous studies (1). Two independent sequences targeting HMGB1 (siHMGB1_1: 5'-GCAAAAUGUCAUCAUAUGCAUUUTT -3' (Duplex) _CDS exon 2 targeting siHMGB1_2: 5'-CCAUUGGUGAUGUUGCGAAGAAACT -3' (Duplex)_ CDS exon 4 targeting) were obtained from IDT.

Mouse strains. C57BL/6-Tg(TRAMP)8247Ng/J (TRAMP); B6.129S4-*Prkn*^{tm1Shn}/J (PRKN$^{-/-}$), Nu/Nu (*Foxn1*^{nu}) and C57BL/6J (WT) mice were purchased from Jackson Laboratory. Female TRAMP mice were crossed with male PRKN knockout (KO) mice and the male progeny carrying both TRAMP transgene and PRKN deletion (TRAMP-PRKN KO) was further

expanded with analysis of tumor formation. B6(Cg)-Ifnar1tm1.2Ees/J (IFNAR$^{-/-}$) mice breeders were purchased from Jackson Laboratory and colonies were maintained and genotyped as recommended by the supplier.

Cells and cell lines. Human prostate cancer PC3 and DU145, human breast adenocarcinoma MDA231, human pancreatic ductal adenocarcinoma PANC-1, normal human mammary epithelial MCF10, murine prostate cancer TRAMP-C2 and murine breast adenocarcinoma AT3 cells were purchased from the American Type Culture Collection (ATCC) and maintained in culture at 37°C in a humidified incubator in the presence of 5% CO_2, according to the supplier's specifications. Murine prostate cancer MP3098 and MPTEN1 cell lines were described previously (2). Briefly, prostate tumors were minced, digested in DMEM media containing 3 mg/ml dispase II (Gibco) and 1 mg/ml collagenase IV (C5138; Sigma) for 1 h at 37°C, and plated on 10-cm culture dishes coated with 100 μg/ml collagen (PureCol; 5005; Advanced Biomatrix). Primary cultures were passaged at least three times to remove fibroblast contamination. All prostate cancer cell lines were maintained in a humidified incubator at 37°C with 5% CO_2 and grown in DMEM supplemented with 10% FBS and 100 IU/ml penicillin/streptomycin. For all cell types, cell passaging was limited to <40 passages from receipt and cell lines were authenticated by STR profiling with AmpFlSTR Identifiler PCR Amplification Kit (Life Technologies) at the Wistar Institute's Genomics Shared Resource. Mycoplasma-free cultures were confirmed at the beginning of the studies and every 2 months afterwards by direct polymerase chain reaction (PCR) of cultures using Bioo Scientific Mycoplasma Primer Sets (#375501) and Hot Start polymerase (QIAGEN).

In some experiments, the various tumor cell lines were treated with the pyrimidine nucleoside analog and DNA hypomethylating agent, decitabine (5-10 μM, Selleckchem) for 24-72 h or the

mitochondrial-directed superoxide dismutase inhibitor, MitoTempo and analyzed by Western blotting or RT-qPCR. PC3 cells stably transfected with doxycycline (Doxy)-inducible TetON PRKN cDNA have been described (3). To obtain TRAMP-C2 cells with conditional expression of PRKN in response to Doxy, cultures were infected with Dual Tet vector system viral particles (Vector Builder) using a co-transduction approach. Upon reaching 50% confluency, the various cultures were infected with lentiviral particles encoding PRKN (pLV[Exp]-Puro-TRE>mPrkn[NM_016694.4]-Vector ID:VB220330-1318vfp) and tTA (pLV[Exp]-CMV>tTS/rtTA/Hygro-Vector ID:VB010000-9369xhm) at multiplicity of infection (m.o.i.) of 10 in growth medium supplemented with tetracycline-free FBS (TetFree FBS, Gibco) and 5 µg/ml polybrene. After 24 h, transduced cells were washed and maintained in growth medium with TetFree FBS containing 150 µg/ml hygromycin (Millipore Sigma) and 2 µg/ml puromycin (Millipore Sigma). Conditional PRKN expression was examined in the presence of increasing concentrations (1-1000 ng/ml) of Doxy (Millipore Sigma) after 72 h by Western blotting. Subsequent experiments were performed using 100 ng/ml Doxy to conditionally induce PRKN expression in TRAMP-C2 cells. After 10 passages in the presence of selection antibiotics, TRAMP-C2 cells stably expressed the PRKN TetON system and were subsequently cultured in the absence of antibiotics.

Transfections. Plasmid cDNA encoding PRKN or E3 ligase-defective PRKN Cys431Ser (C431S) or Ser65Ala (S65A) mutants have been described (3). For transient transfection, the various cell lines were seeded on a 6-well plate in 3 ml of growth medium for 16 h at 37°C. Cultures were incubated with 2 µg of plasmid cDNA plus 4 µl of XtremeGENE™ 9 DNA - Transfection Reagent (Millipore Sigma) in 100 µl Opti-MEM™ (Gibco). Recombinant protein expression was assessed after 72 h by Western blotting. In some experiments, PRKN TetON PC3

cells were transfected with NFκB-Luc NanoLuc® Reporter Vector with NFκB Response Element (Promega, cat. n. #1111), IFNβ-Luc promoter construct (Addgene, cat. n. #102697), WT IFIT1-Luc promoter construct (-565/+3) or mutant IFIT1-Luc promoter construct carrying a double mutation in the ISRE sites (-117/-105; -104/-92) (4) and analyzed for luciferase reporter activity in the presence of vehicle or Doxy.

NFκB Taqman Gene Array. For Human NFκB TaqMan™ Array (Applied Biosystems), RNA was isolated from control or PRKN-expressing PC3 cells, and 10 ng of cDNA was used per reaction. cDNA was pre-combined with TaqMan™ Fast Advanced Master Mix (Applied Biosystems). Twenty µl of mix was added to each well of the assay plate, sealed with MicroAmp Optical Adhesive Film, and centrifuged briefly to bring the contents to the bottom of the wells. qPCR reactions were performed on an ABI quant Studio 5 (Applied Biosystems) using the following reaction settings: UNG incubation, 2 min at 50ºC; enzyme activation, 20 sec at 95ºC; 40 cycles of denaturing and annealing; 3 sec at 95ºC followed by 30 sec at 60ºC. ΔΔct calculations were performed in Excel and imported to Prism 10 for statistical analysis and graphical presentation. A similar amplification protocol was used for analysis of an IFN RT-qPCR array in control or PRKN-expressing PC3 cells.

Cytokine detection in mouse plasma. Aliquots of plasma collected from C57BL/6, TRAMP or TRAMP-PRKN KO mice were stored at -80°C and circulating levels of IL6 or IFNα were determined using Quantikine ELISA kit (R&D system) following the manufacturer's specifications. A Promega™ GloMax® Plate Reader (Promega) was used for signal quantification.

DAMP quantification. PRKN TetON PC3 cells on a 96-well plate ($2x10^3$ cells/well) were treated with Doxy (100 ng/ml) for 72 h at 37°C. For quantification of extracellular ATP, a 75 µl aliquot of CM from each well was transferred to white bottom 96 well-plates, mixed with 25 µl 4X RealTime-Glo™ extracellular ATP assay reagent (GA5010, Promega) and changes in luminescence were measured using a GloMax plate reader (Promega). Alternatively, aliquots of TetON PRKN PC3 cells with or without Doxy were processed for isolation of genomic DNA (K0721, Thermo Scientific) or cytosolic fractions by incubation in permeabilization buffer (150 mM NaCl, 50 mM HEPES, pH 7.4, 200 µg/ml digitonin, and 1M hexylene glycol) for 10 min at 4°C followed by centrifugation at 2000 g for 10 min at 4°C. Samples were treated with proteinase K and RNase A and cytosolic DNA was collected by phenol-chloroform-isoamyl alcohol extraction. Each pellet was dissolved in equal volume of ddH$_2$O, and amplification of mitochondrial DNA (mtDNA)-encoded ND1 and ND4 genes was quantified in cytosol by RT-qPCR. The following primers were used for mtND1 (Forw. AACCTCAACCTAGGCCTCCT; Rev. GAGTTTGATGCTCACCCTGA) and mtND4 (Forw. CGGCGCAGTCATTCTCATAA, Rev. GACTGTGAGTGCGTTCGTAG). Primers for β2M and TERT were used as control for genomic DNA. In other experiments, TetON PRKN PC3 cells ($2x10^5$ cells per well in a 6-well plate) were cultured for 48 h in the presence of Doxy, lysed directly in the plate using M-PER mammalian protein extraction reagent (Thermo Scientific #778503) and concentrated using Millipore Microcon-10 centrifugal filters with Ultracel 10 regenerated cellulose 10,000 NMWL membranes. The concentrated samples were then used for quantification of 2'3' cGAMP by ELISA, according to the manufacturer's specifications (Cayman Chemicals No. 501700).

Methylation-specific qPCR. Genomic DNA was extracted with the GeneJet Genomic DNA purification kit (Thermo Fisher Scientific) according to the manufacturer's protocol. The EZ

DNA Methylation-Gold Kit (Zymo Research) was used to bisulfite convert 1 µg of DNA following the manufacturer's protocol and eluted in 10 µl of elution buffer. A nested PCR amplification step was performed on the bisulfite-treated DNA to amplify the PRKN promoter target CpG and a housekeeping target in the ACTB gene loci devoid of CpG sites. The following primers sets were used: PRKN Forward: 5'-AAC AAT TTA TAA ACC TAA TTA AAC CCA and Reverse: 5'-GAT AAT ATG GTT GTT GAA TTA GGA GA and ACTB Forward: 5'-TAT ATA GGT TGG GGA AGT TTG and Reverse: 5' -TAT AAA AAC ATA AAA CCT ATA ACC. The PCR amplification reaction was performed using EpiMark Hot Start Taq DNA Polymerase (New England Biolabs) 1.25 U/50 µl reaction volume, 200 µM dNTPs, 0.2 µM primers sequences, 1x EpiMark Hot Start Taq Reaction buffer, and 1 µg of bisulfite-treated DNA. PCR cycles included an initial denaturation step at 95°C for 30 sec, followed by 10 cycles at 95°C for 30 sec, 55°C for 30 sec, and 68°C for 45 sec, concluding with a final extension at 68°C for 5 min. A qPCR amplification step was performed using methylation-specific primer sequences overlapping with CpG sites within the *PARK2* promoter. For each reaction 2 µl of the 20 µl nested PCR reaction was added. qPCR reactions were performed using a QuantStudio 5 Real-Time PCR system (Thermo Fisher Scientific) using 1x Maxima SYBR Green/ROX qPCR Master Mix (Thermo Fisher Scientific) and 500 nM forward and reverse primers. The following primers were used: PRKN Forward: 5'-GGT CCC CAA CTT AAA AAT AAT AAT AGC G and Reverse: 5'-CTG TAG AAT AGA TTT GTA AAT AAA TTT GAG T. The PRKN-specific primers target two sequential CpG sites located at chr6:162728136 on reference genome hg38 and are approximately 200 bp upstream of the PRKN transcriptional start site.

Immunohistochemistry. Tissue samples harvested from the various animal groups were fixed in 10% neutral buffered formalin solution (ThermoFisher Scientific) for 16 h at 22°C, transferred to

70% ethanol and embedded in paraffin. Five μm tissue sections were deparaffinized by sequential passages in xylene/ethanol solutions, hydrated and incubated with primary antibodies to designated target antigens. Slides were stained with hematoxylin-eosin (H&E) (Leica, Surgipath Hematoxylin 560) using an automated stainer (Dakewe Medical Equipment). For detection of intratumoral CD8 T cells, tissue sections were subject to antigen retrieval in a pressure cooker for 20 min at 95°C in the presence of DAKO EDTA. Slides were incubated with rabbit anti-mouse CD8α (D4W2Z) XP® antibody (1:200) for 16 h at 4°C and DAB was maintained for 90 sec. For intratumoral detection of Doxy-induced conditionally expressed PRKN, tissue slides of AT3 tumors were processed for antigen retrieval in a steamer for 20 min in the presence of citrate buffer and incubated with rabbit anti-PRKN antibody (1:400) at 4°C. DAB was added for 2 min. For detection of intratumoral HMGB1 after systemic administration of decitabine, tissue slides of TRAMP-C2 tumors were incubated with an antibody to HMGB1 (1:200, ProteinTech) for 16 h at 4°C and DAB addition for 4 min. Secondary HRP horse anti-rabbit IgG polymer reagent (1:200) was incubated for 30 min at 22°C. Slide acquisition images were taken with a 80i Nikon microscope or NanoZoomerS60 digital scanner (Hamamatsu). Immunohistochemical staining was quantified using QuPath software.

SUPPLEMENTAL FIGURES

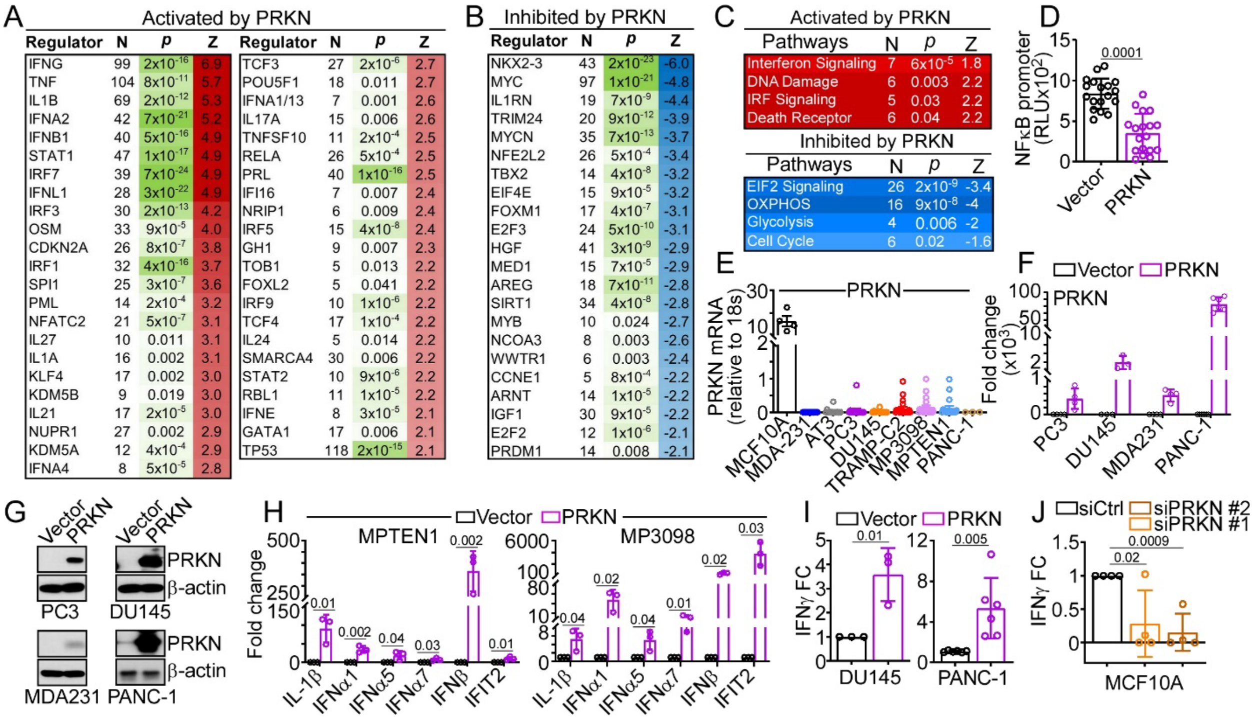

Fig. S1. PRKN IFN gene expression in cancer. (A and B) PC3 cells expressing PRKN were analyzed by RNA-Seq and genes upregulated (A) or downregulated (B) in the presence of PRKN compared to vector are indicated. (C) The RNA-Seq dataset of PRKN-expressing PC3 cells in (A) was examined by Ingenuity Pathway Analysis with identification of cellular networks activated (*top*) or inhibited (*bottom*) in the presence of PRKN. For panels A-C, a p value and Z score for each gene product are indicated. (D) PC3 cells were transfected with vector or PRKN and analyzed for NFκB promoter reporter luciferase activity. RLU, relative luciferase units. Mean±SD (n=3). (E) The indicated human (PC3, DU145, MDA231, PANC-1, MCF10A) or murine (AT3, TRAMP-C2, MP3098, MPTEN1) cell lines were analyzed for endogenous PRKN expression by RT-qPCR. Mean±SD (n=3). (F and G) The indicated tumor cell types were transfected with vector or PRKN and analyzed by RT-qPCR (F) or Western blotting (G). Mean±SD (n=3). (H) Murine prostate cancer MPTEN1 or MP3098 cells expressing vector or

PRKN were analyzed for IFN gene expression by RT-qPCR. Mean±SD (n=3). (I) The indicated tumor cell lines expressing vector or PRKN were analyzed for IFNγ mRNA expression by RT-qPCR. Mean±SD (n=3). (J) Normal mammary epithelial MCF10A cells expressing endogenous PRKN were transfected with control, non-targeting siRNA or two independent PRKN-directed siRNA sequences (siPRKN #1 and siPRKN #2) and analyzed for IFNγ mRNA expression by RT-qPCR. Mean±SD (n=3). Numbers represent p values by two-tailed unpaired t test.

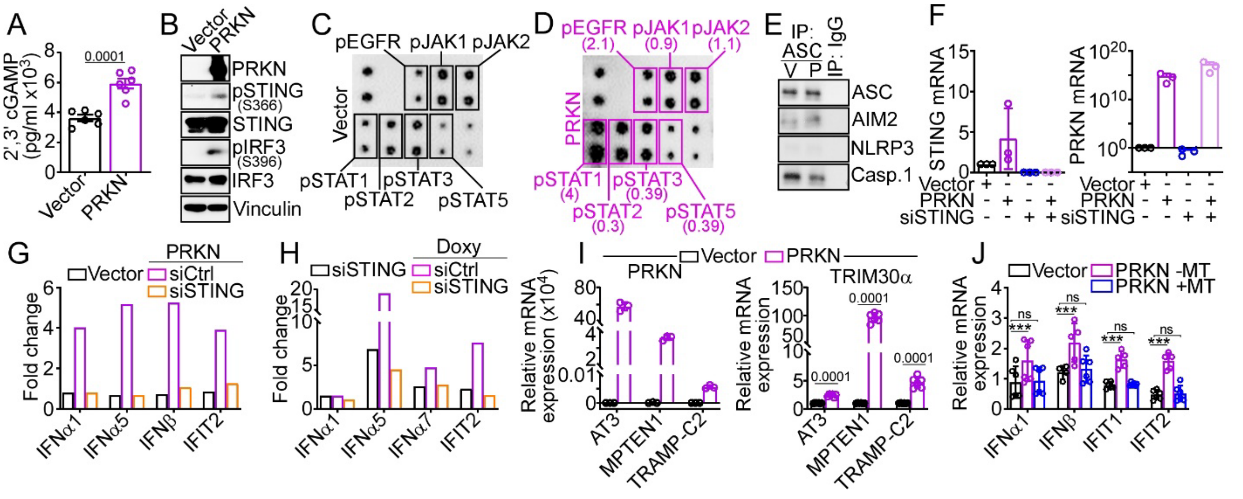

Fig. S2. Requirements of PRKN IFN gene expression. (A) PC3 cells expressing vector or PRKN were analyzed for 2',3' cGAMP content by ELISA. Mean±SD (n=3). A p value by two-tailed unpaired t test is indicated. (B) PC3 cells as in (A) were analyzed by Western blotting. p, phosphorylated. (C and D) The conditions are as in (A) and cellular extracts from PC3 cells expressing vector (C) or PRKN (D) were analyzed in a JAK/STAT array by Western blotting. Numbers indicate normalized changes in protein expression quantified by densitometry. Representative experiment. (E) PC3 cells expressing vector (V) or Parkin (P) were immunoprecipitated (IP) with an antibody to ASC or non-binding IgG and immune complexes were analyzed for the indicated associated proteins, by Western blotting. Representative experiment. (F and G) PC3 cells expressing vector or PRKN were transfected with control, non-targeting siRNA (siCtrl) or STING-directed siRNA (siSTING) and analyzed for changes in STING (*left*) or PRKN (*right*) mRNA levels (F) or modulation of IFN gene expression (G) by RT-qPCR. Representative experiment out of 4 independent determinations. (H) PC3 cells with conditional expression of PRKN (TetON system) induced by doxycycline (Doxy) were transfected with siCtrl or siSTING and analyzed for IFN gene expression. Representative experiment. (I) The indicated murine tumor cell lines expressing vector or PRKN were analyzed

for modulation of PRKN (*left*) or TRIM30α (*right*) mRNA expression by RT-qPCR. Mean±SD (n=3) (J) PC3 expressing vector or PRKN were analyzed for IFN gene expression in the presence or absence of the mitochondrial superoxide scavenger MitoTempo (MT), by RT-qPCR. Mean±SD (n=4). A p value was obtained by 2-way ANOVA. ***, $p<0.0001$.

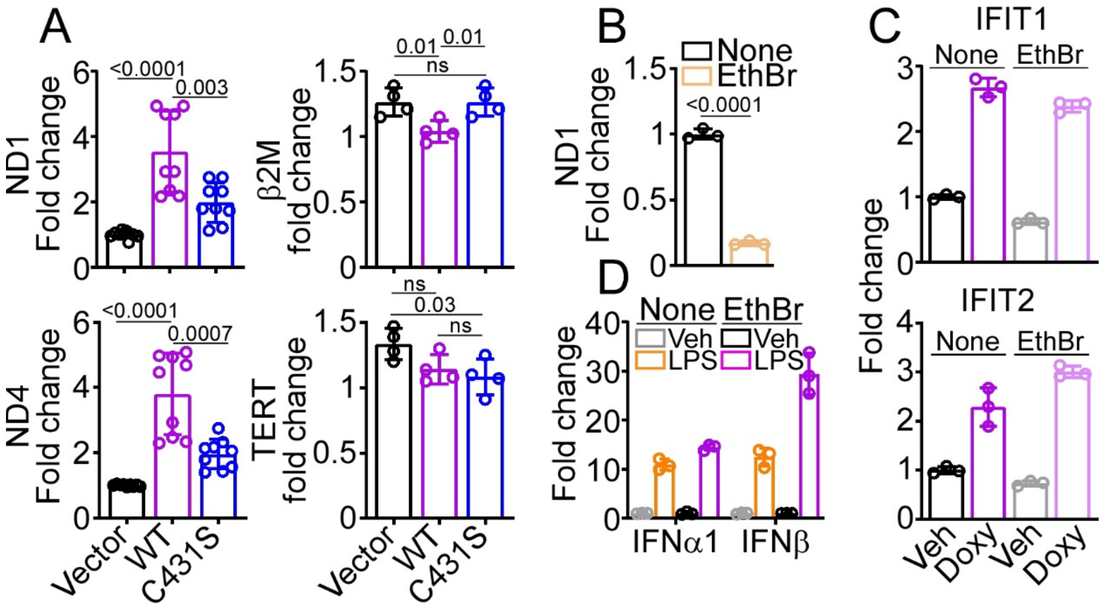

Fig. S3. Mechanisms of PRKN IFN signaling. (A) Cytosolic extracts from PC3 cells expressing vector, wild type (WT) PRKN or E3 ligase loss-of-function C431S PRKN mutant were analyzed for mtDNA-encoded ND1 (*top*) or ND4 (*bottom*) genes by RT-qPCR (*left*). Amplification of nuclear-encoded β2M (*top*) or TERT (*bottom*) gene products was used as control (*right*). Mean±SD (n=4). (B and C) PC3 cells conditionally expressing PRKN in response to Doxy were grown in the presence of ethidium bromide (EthBr) for 8 d and analyzed for modulation of ND1 (B), IFIT1 (C, *top*) or IFIT2 (C, *bottom*) gene expression, by RT-qPCR. Mean±SD (n=4). (D) Parental or EthBr-treated PC3 cells as in (B and C) were stimulated with vehicle (Veh) or LPS and analyzed for IFN gene expression, by RT-qPCR. Mean±SD (n=4). Numbers represent p values by two-tailed unpaired t test. ns, not significant.

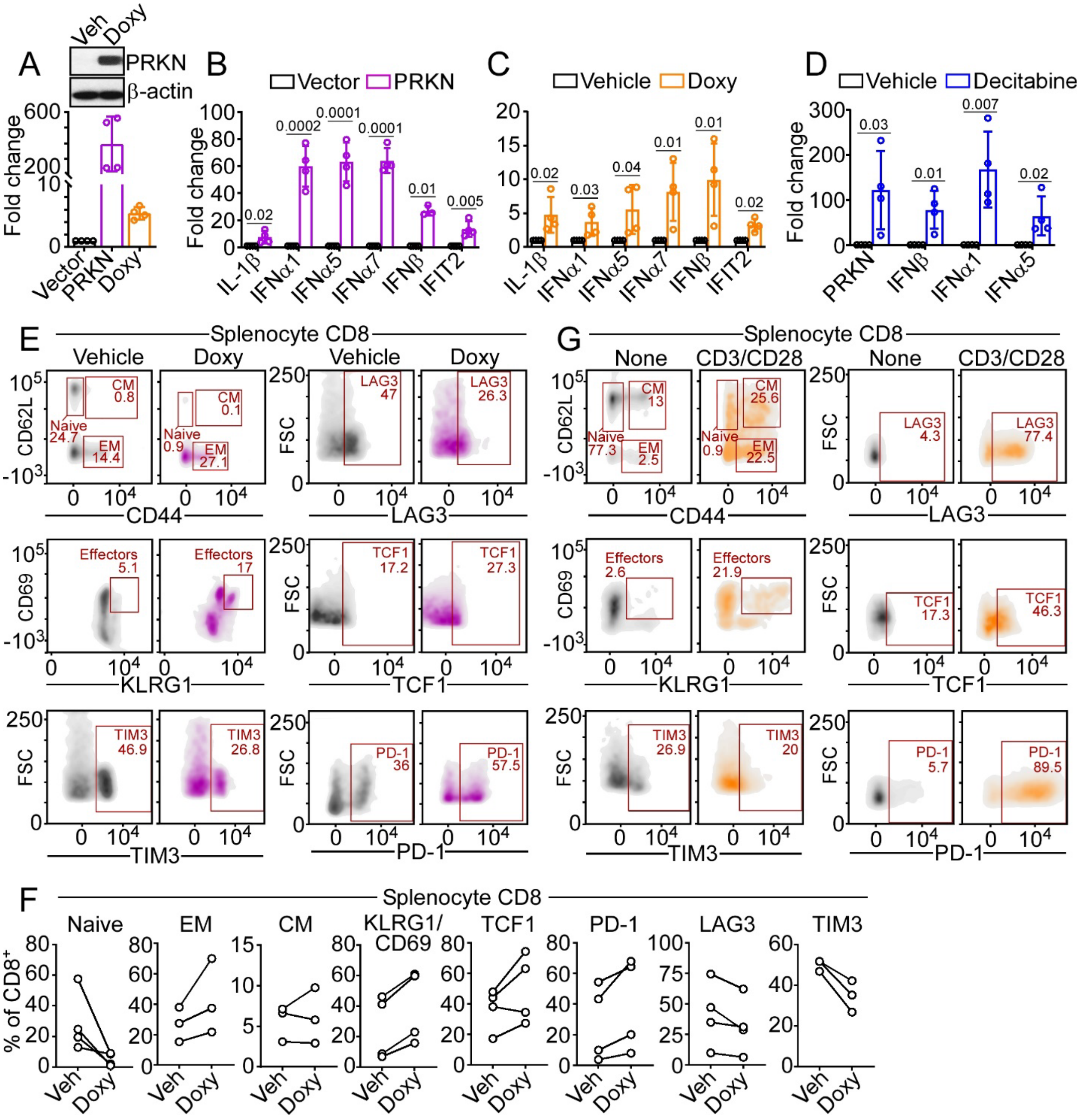

Fig. S4. PRKN paracrine activation of CD8 T cells. (A) Murine prostate cancer TRAMP-C2 cells transiently transfected with PRKN cDNA (PRKN) or conditionally expressing PRKN in response to Doxy (TetON system) were analyzed by RT-qPCR or Western blotting (*inset*). Mean±SD (n=4). (B and C) TRAMP-C2 cells transiently transfected with vector or PRKN (B) or conditionally expressing PRKN in response to Doxy (C) were analyzed for IFN gene expression

by RT-qPCR. Mean±SD (n=4). (D) Parental TRAMP-C2 cells were treated with vehicle or decitabine and analyzed for IFN gene expression by RT-qPCR. Mean±SD (n=4). (E) Splenocytes from C57BL/6 mice were incubated with aliquots of conditioned medium (CM) from PRKN TetON TRAMP-C2 cells treated with vehicle or Doxy and analyzed for modulation of the indicated CD8 T cell markers after 72 h by multiparametric flow cytometry. The percentage of cells in each quadrant is indicated. Representative density plots are shown. CM, central memory; EM, effector memory. (F) The conditions are as in (E) and modulation of the indicated CD8 T cell markers in splenocytes after co-culture with vehicle (Veh) or Doxy-induced PRKN CM was quantified by flow cytometry in 3-4 independent experiments. Each symbol corresponds to an independent mouse sample. (G) Splenocytes from C57BL/6 mice were incubated with plate-immobilized antibodies to CD3 and CD28 and modulation of the indicated CD8 T cell markers was quantified by flow cytometry. Representative density plots are shown. The percentage of cells in each quadrant is indicated. Numbers represent p value by two-tailed unpaired t test.

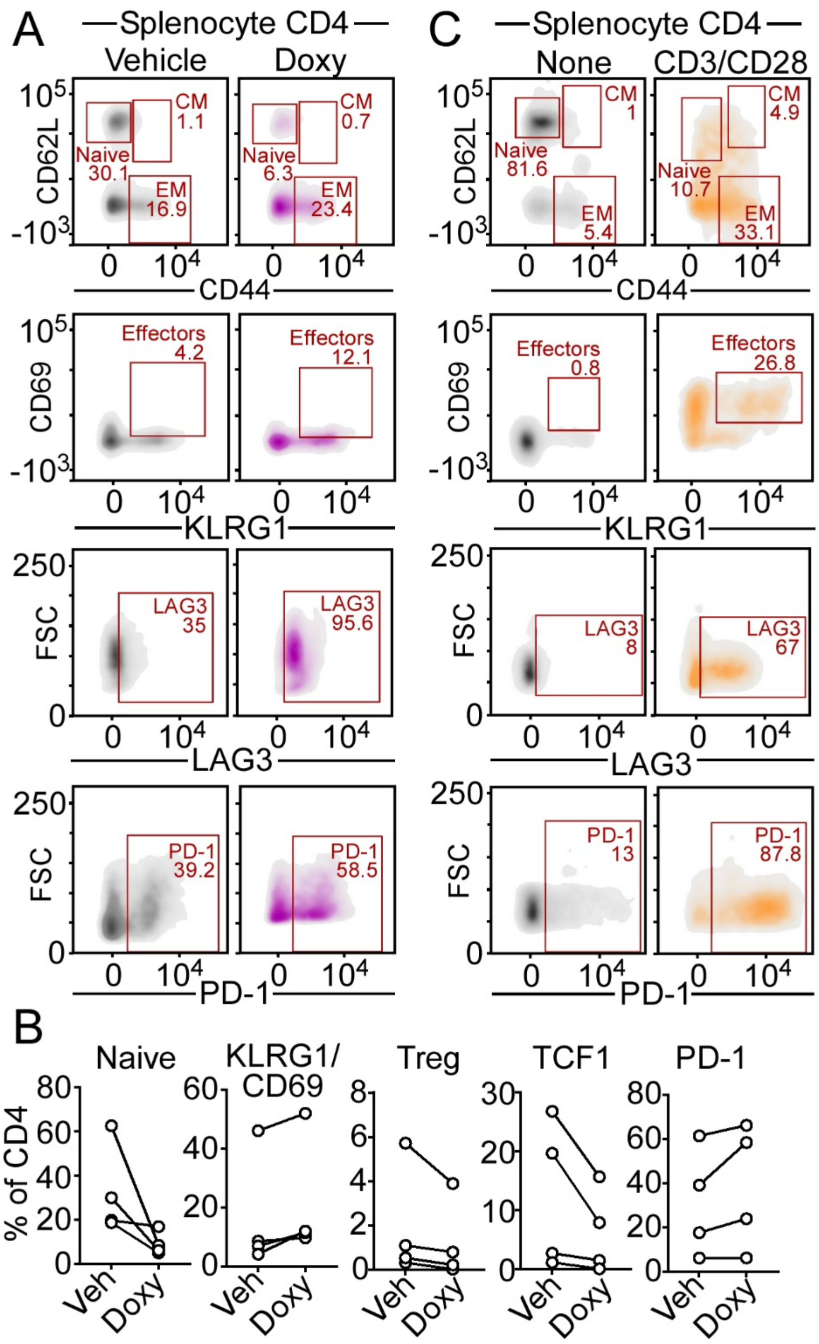

Fig. S5. PRKN modulation of CD4 T cells. (A) Splenocytes from C57BL/6 mice were incubated with CM from PRKN TetON TRAMP-C2 cells treated with vehicle (Veh) or Doxy and analyzed for modulation of the indicated CD4 T cell markers after 72 h by multiparametric flow cytometry. The percentage of cells in each quadrant is indicated. Representative density plots are

shown. CM, central memory; EM, effector memory. (B) The conditions are as in (A) and modulation of the indicated CD4 T cell markers by PRKN CM in the presence of vehicle (Veh) or Doxy was quantified in four independent experiments. (C) Splenocytes from C57BL/6 mice were incubated with plate-immobilized antibodies to CD3 and CD28 and modulation of the indicated CD4 T cell markers was quantified by flow cytometry. Representative density plots are shown. The percentage of cells in each quadrant is indicated.

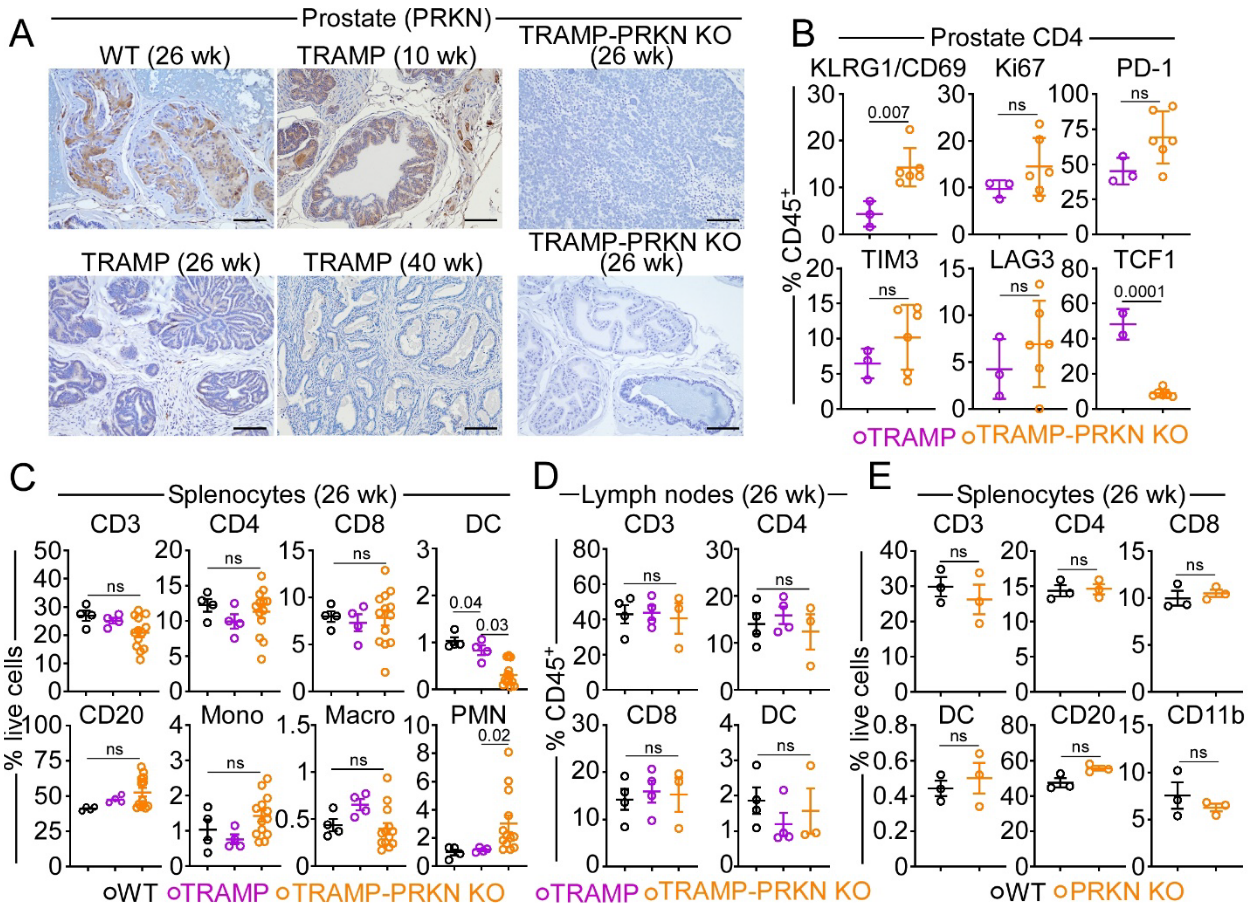

Fig. S6. PRKN immune modulation of TRAMP tumorigenesis. (A) Prostate tissues harvested from C57BL/6 (WT), TRAMP or TRAMP-PRKN KO mice were analyzed for expression of PRKN at the indicated weeks (wk) of age by immunohistochemistry (IHC). Representative images are shown. Scale bar, 50 µm. (B) Prostate tissues harvested from TRAMP or TRAMP-PRKN KO mice at 26 wk were analyzed for the indicated CD4 T cell markers by flow cytometry. (C and D) Spleens (C) or loco-regional pelvic lymph nodes (D) harvested from TRAMP or TRAMP-PRKN KO mice at 26 wk were analyzed for modulation of the indicated immune cell subsets by flow cytometry. DC, dendritic cells; Mono, monocytes; Macro, macrophages; PMN, polymorphonuclear leukocytes. (E) Splenocytes harvested from C57BL/6 (WT) or PRKN KO mice at 26 wk were analyzed for modulation of the indicated immune cell

subsets by flow cytometry. For all panels, mean±SD. Numbers represent p values by two-tailed unpaired t test. ns, not significant.

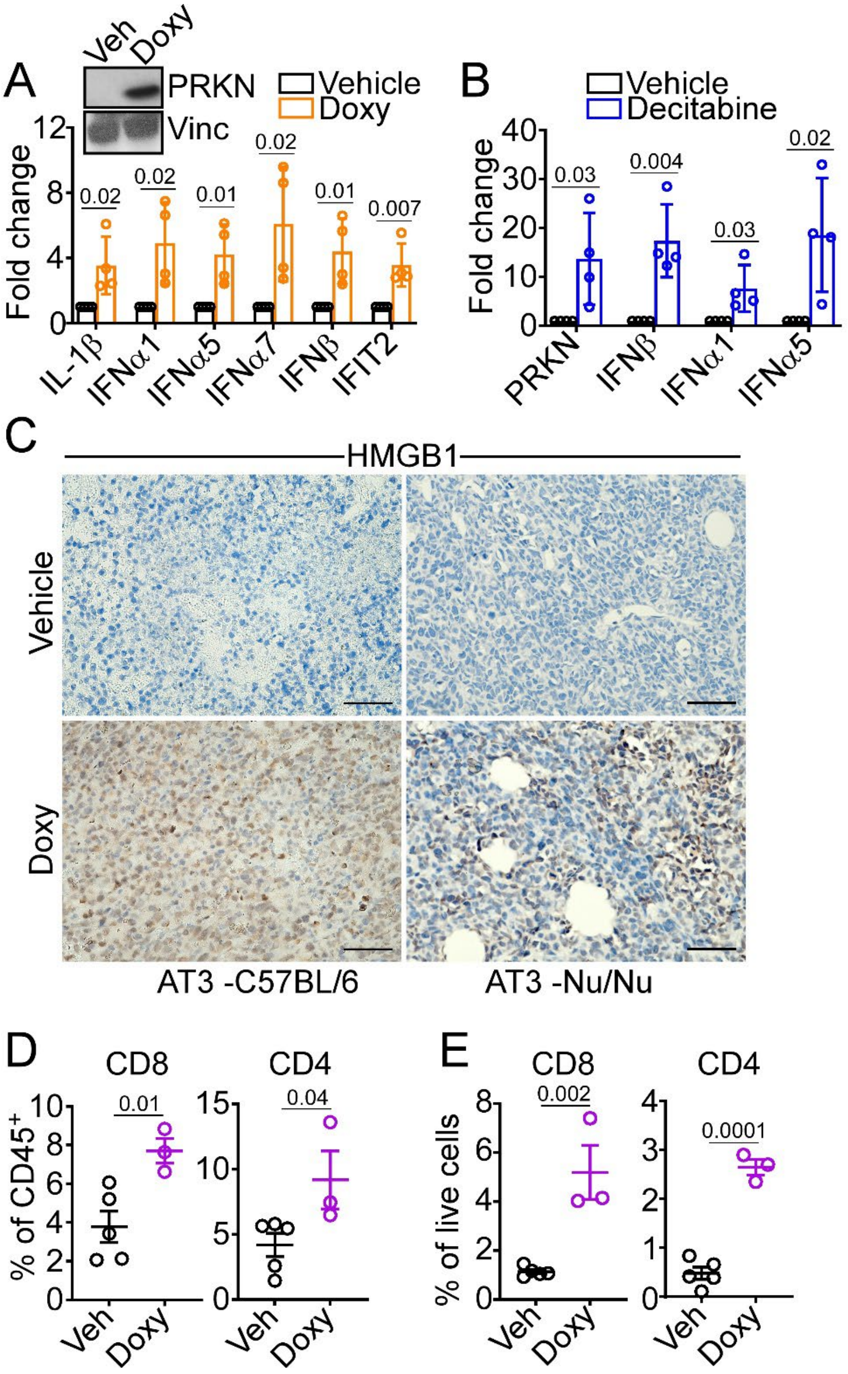

Fig. S7. PRKN regulation of AT3 mammary gland tumorigenesis. (A) Murine mammary adenocarcinoma AT3 cells engineered to conditionally express PRKN in the presence of Doxy (TetON system) were analyzed by Western blotting (*inset*) or IFN gene expression by RT-qPCR. Mean±SD (n=4). (B) Parental AT3 cells were treated with vehicle or decitabine and analyzed for

IFN gene expression by RT-qPCR. Mean±SD (n=4). (C) Prostate tissues harvested from AT3 syngeneic mammary gland tumors grown in the presence of vehicle (Veh) or Doxy in C57BL/6 (*left*) or immunocompromised nude (Nu/Nu) (*right*) mice were analyzed for expression of HMGB1 by IHC. Representative images are shown. Scale bar, 50 μm. (D and E) AT3 syngeneic mammary gland tumors grown in the presence of vehicle (Veh) or Doxy in C57BL/6 mice were harvested at the end of the experiment and analyzed for differential accumulation of CD8 or CD4 T cell subsets by $CD45^{+}$ gating (D) or live cell fluorescence (E) by flow cytometry. Mean±SD (n=3-5). Numbers represent p values by two-tailed unpaired t test.

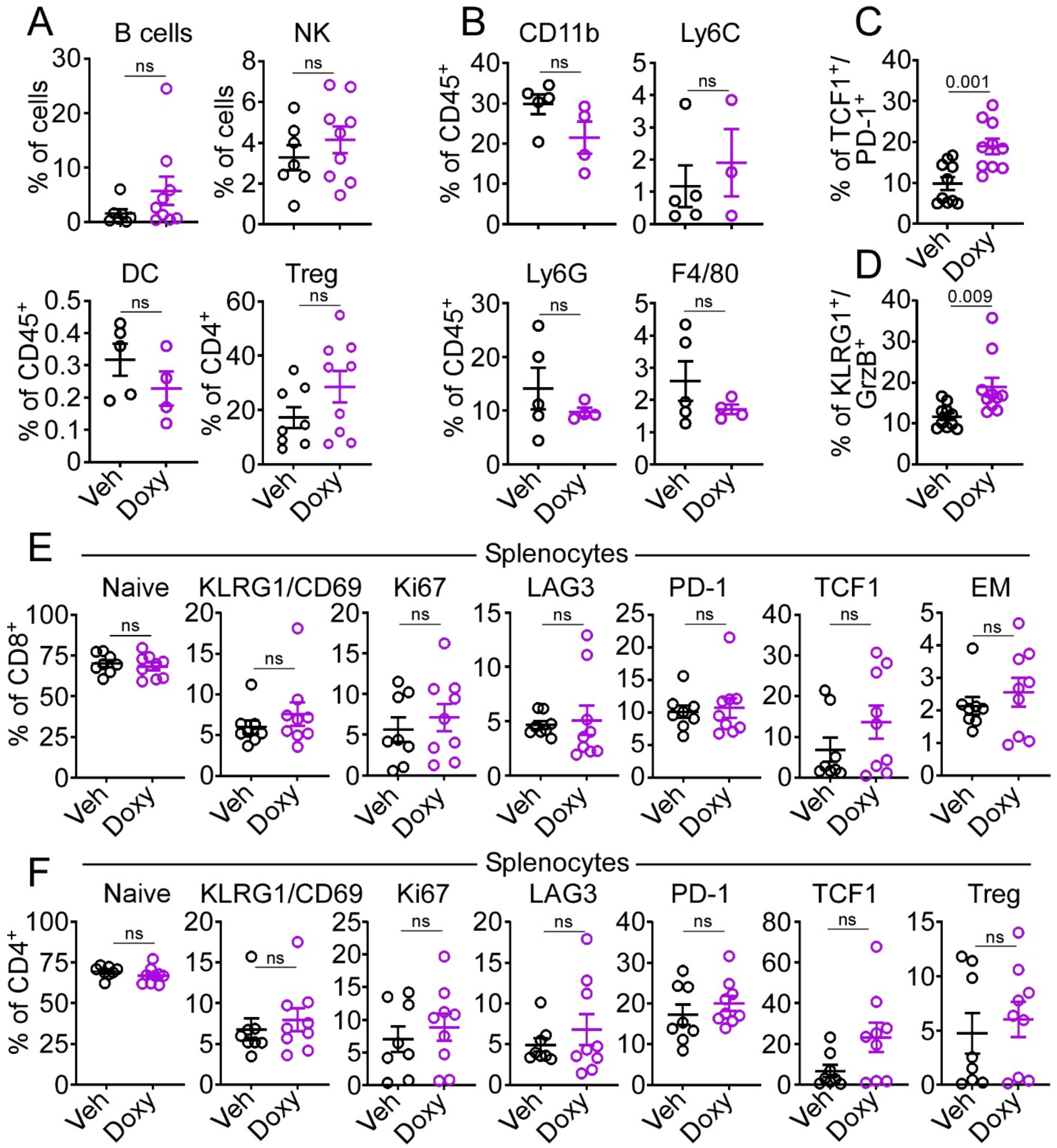

Fig. S8. PRKN immune modulation in syngeneic AT3 mammary gland tumors. (A and B) PRKN TetON AT3 mammary gland tumors grown in C58BL/6 mice in the presence of vehicle (Veh) or Doxy were harvested at the end of the experiment and analyzed for changes in the indicated B (CD20), NK, DC or Treg subsets (A) or myeloid cell subsets (B) by flow cytometry. (C and D) The conditions are as in (A and B) and intratumoral CD8 T cells from C57BL/6 mice treated

with vehicle or Doxy were analyzed for double positive TCF1^{+}/PD-1^{+} (C) or KLRG1^{+}/GrzB^{+} (D) subsets, by flow cytometry. (E and F) Splenocytes from C57BL/6 mice harboring PRKN TetON AT3 syngeneic mammary gland tumors treated with vehicle or Doxy were harvested at the end of the experiment and analyzed for the indicated CD8 (E) or CD4 (F) T cell markers by flow cytometry. For all panels, each point corresponds to an individual mouse determination. Mean±SD. Numbers represent p values by two-tailed unpaired t test. ns, not significant.

SUPPLEMENTAL REFERENCES

1. Agarwal E, Goldman AR, Tang HY, Kossenkov AV, Ghosh JC, Languino LR, et al. A cancer ubiquitome landscape identifies metabolic reprogramming as target of Parkin tumor suppression. Sci Adv. 2021;7(35):eabg7287.
2. Leibold J, Ruscetti M, Cao Z, Ho YJ, Baslan T, Zou M, et al. Somatic Tissue Engineering in Mouse Models Reveals an Actionable Role for WNT Pathway Alterations in Prostate Cancer Metastasis. Cancer Discov. 2020;10(7):1038-57.
3. Yeon M, Bertolini I, Agarwal E, Ghosh JC, Tang HY, Speicher DW, et al. Parkin ubiquitination of Kindlin-2 enables mitochondria-associated metastasis suppression. J Biol Chem. 2023;299(6):104774.
4. Grandvaux N, Servant MJ, tenOever B, Sen GC, Balachandran S, Barber GN, et al. Transcriptional profiling of interferon regulatory factor 3 target genes: direct involvement in the regulation of interferon-stimulated genes. J Virol. 2002;76(11):5532-9.

SUPPLEMENTAL TABLES

Table S1. Primer sequences used for RT-qPCR amplification experiments in this study.

Target gene	Forward	Reverse
Mouse-PRKN	AAGAAGACCACCAAGCCTTGTC	CAAACCAGTGATCTCCCATGC
Mouse-IL1α	ACGGCTGAGTTTCAGTGAGACC	CACTCTGGTAGGTGTAAGGTGC
Mouse IL1β	TGGACCTTCCAGGATGAGGACA	GTTCATCTCGGAGCCTGTAGTG
Mouse IL6	TACCACTTCACAAGTCGGAGGC	CTGCAAGTGCATCATCGTTGTTC
Mouse IFIT2	CGAACTACCGTCTGGATGACTG	CTTCAACCAGCGCCATTGCTTG
Mouse IFNα1	CCAGTGGAAGCAAAGGATTGCC	GCACCTCATGTCCTTCTCAAGC
Mouse IFNα5	GGATGTGACCTTCCTCAGACTC	CACCTTCTCCTGTGGGAATCCA
Mouse IFNα7	TCCTGCCTGAAGGACAGAAAGG	TCCTGCCTGAAGGACAGAAAGG
Mouse IFNβ	GCCTTTGCCATCCAAGAGATGC	ACACTGTCTGCTGGTGGAGTTC
Mouse 18S	CGGAAAATAGCCTTCGCCATCA	ATCACTCGCTCCACCTCATCCT
Human PRKN	CCAGAGGAAAGTCACCTGCGAA	CTGAGGCTTCAAATACGGCACTG
Human IL1α	TGTATGTGACTGCCCAAGATGAAG	AGAGGAGGTTGGTCTCACTACC
Human IL1β	CCACAGACCTTCCAGGAGAATG	GTGCAGTTCAGTGATCGTACAGG
Human IL-6	GACTGTGCACTTGCTGGTGGAT	ACTTCCTCACCAAGAGCACAGC
Human IFIT2	GGAGCAGATTCTGAGGCTTTGC	GGATGAGGCTTCCAGACTCCAA
Human IFNα1	AGAAGGCTCCAGCCATCTCTGT	TGCTGGTAGAGTTCGGTGCAGA
Human IFNα5	GCCTGAGTAACAGGAGGACTTTG	TGAGCCTTCTGGAACTGGTTGC
Human IFNα7	GAAGACTCAAGCCATCTCTGTCC	TAGGAGGCTCTGTTCCCAAGCA
Human IFNβ	CTTGGATTCCTACAAAGAAGCAGC	TCCTCCTTCTGGAACTGCTGCA
#1 Human TRIM30α	AGCCTGCATCACACTGAATTA	TCCCTTGAGCCTCTCTACTATG
#2 Human TRIM30α	GCCGAGTTCCTTACCCATTT	TCCTCCTCTGGAATGGACTT
Human 18S	ACCCGTTGAACCCCATTCGTGA	GCCTCACTAAACCATCCAATCGG

Table S2. Antibodies used for the characterization of PRKN immune modulation.

Antibody ID	Location	Clone	Fluorochrome	Company	Cat #
CD3	Surface	17A2	BUV395	BD Biosciences	740268
CD4	Surface	GK1.5	APC	BioLegend	100411
CD4	Surface	RM4	BV510	BD Biosciences	563106
CD8	Surface	53-6.7	BUV805	BD Biosciences	612898
CD8	Surface	53-6.7	BV421	BD Biosciences	563898
CD11b	Surface	M1/70	BV421	BioLegend	101235
CD11c	Surface	N418	APC	BioLegend	117309
CD19	Surface	1D3	BV750	BD Biosciences	747332
CD25	Surface	PC61	BB515	BD Biosciences	564424
CD44	Surface	IM7	BUV496	BD Biosciences	741057
CD45	Surface	I3/2.3	AF-700	BioLegend	147716
CD45.2	Surface	104	BUV737	BD Biosciences	612728
CD62L	Surface	Mel14	BV650	BD Biosciences	564108
CD69	Surface	H1.2F3	BV785	BioLegend	104543
CX3CR1	Surface	Z8-50	BUV605	BD Biosciences	563106
KLRG1	Surface	2f1	APC- Cy7	BioLegend	138425
CLASS II	Surface	AF6-120.1	PE-Cy7	BioLegend	116417
Ly6C	Surface	AL-21	PE-Cy7	BD Biosciences	560693
Ly6G	Surface	1A8	FICT	BD Biosciences	551460
LAG3 (CD223)	Surface	C9B7W	PE	BioLegend	125207
PD-1 (CD279)	Surface	29F.1A12	PE-Cy7	BioLegend	135215
F4/80	Surface	T45-2342	PE	BD Biosciences	565420
Tim-3 (CD366)	Surface	5D12-TIM-3	BB700	BD Biosciences	747319
NK1.1	Surface	S17016D	PE-Cy5	BioLegend	156423
FOXP3	Intracellular	FJK-16s	EF450	ThermoFisher	5016374
Ki67	Intracellular	B56	PE-CF594	BD Biosciences	567120
TCF-7	Intracellular	S33-966	R718	BD Biosciences	567587
GRANZYME B	Intracellular	NGZB	APC	ThermoFisher	17889880
