# Supplementary material for: Parkin activates innate immunity and promotes antitumor immune responses
Source: J Clin Invest. 2024 Aug 30;134(22):e180983. doi: 10.1172/JCI180983 (PMC11563675; doi:10.1172/JCI180983)

Figure 1F

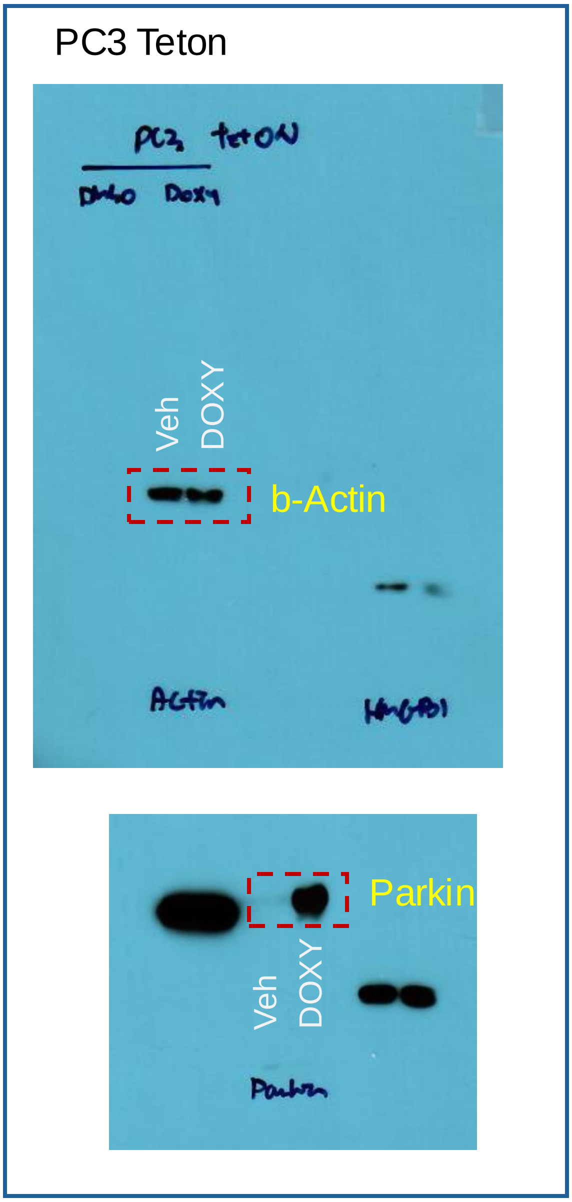

Figure 1J

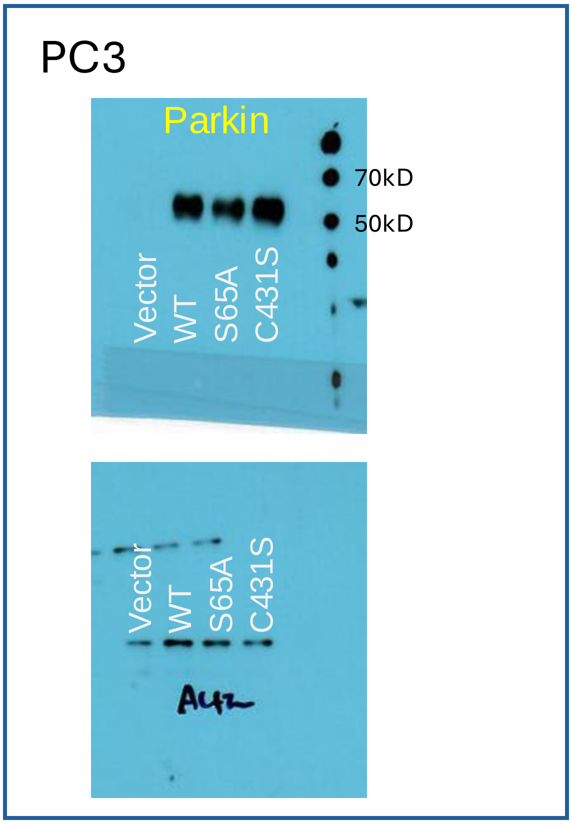
PC3
Parkin
70kD
50kD
Vector
WT
S65A
C431S
Vector
WT
S65A
C431S

Figure 2E

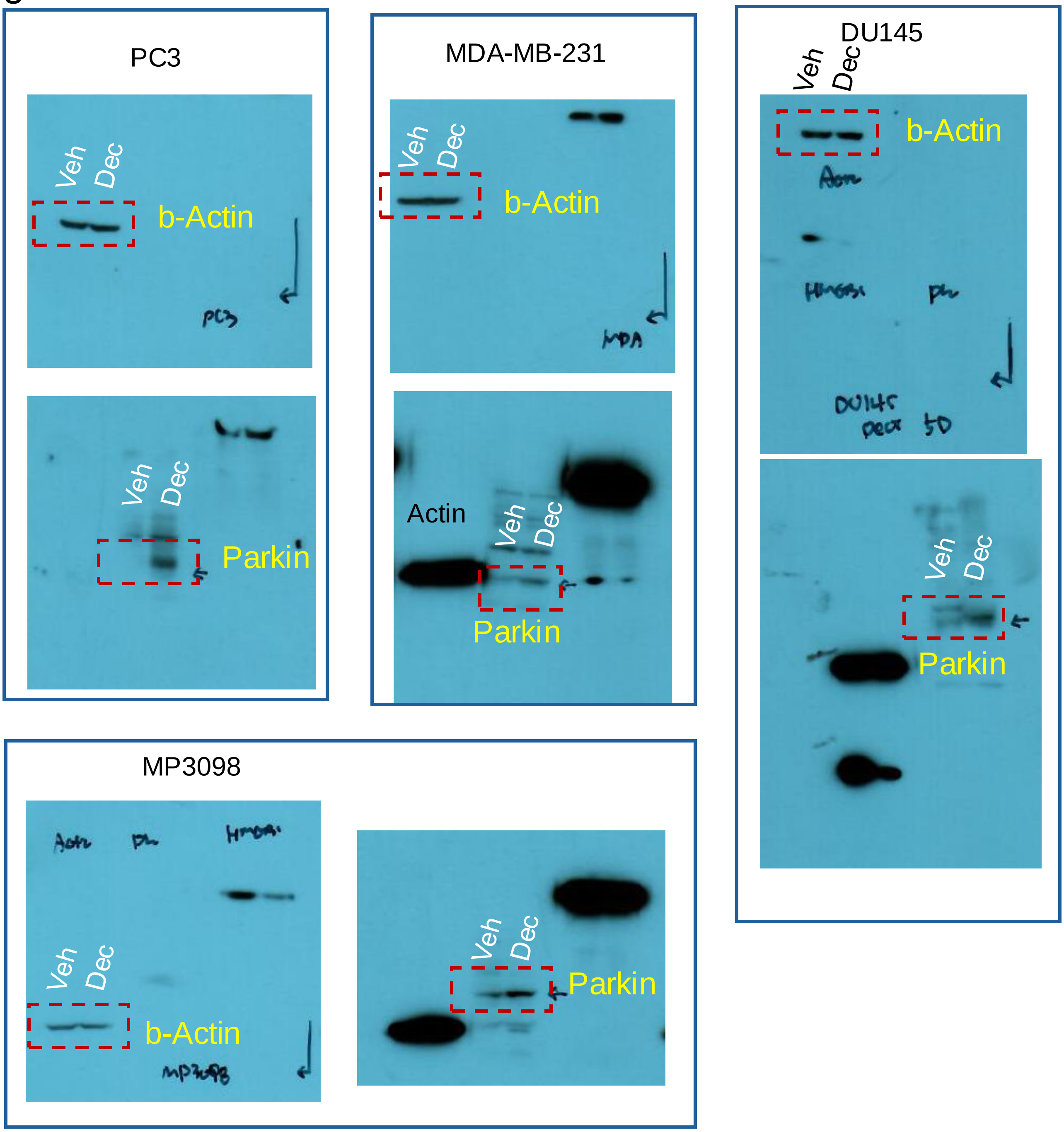
PC3
Veh
Dec
b-Actin
Parkin
MDA-MB-231
b-Actin
Actin
Parkin
DU145
b-Actin
Parkin
MP3098
b-Actin
Parkin

Figure 3A

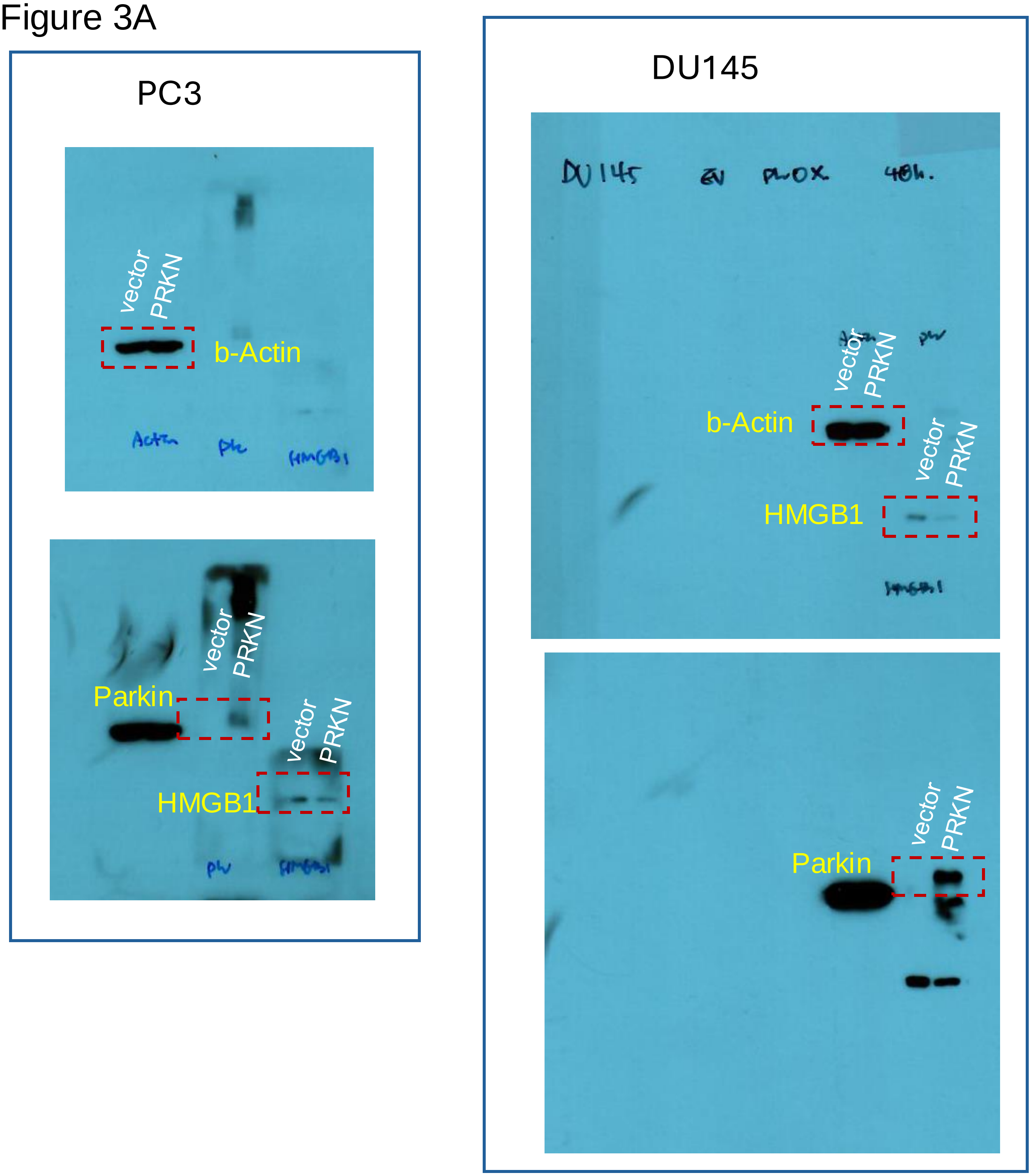
PC3
vector
PRKN
b-Actin
Parkin
HMGB1
DU145
b-Actin
HMGB1
Parkin

Figure 3A

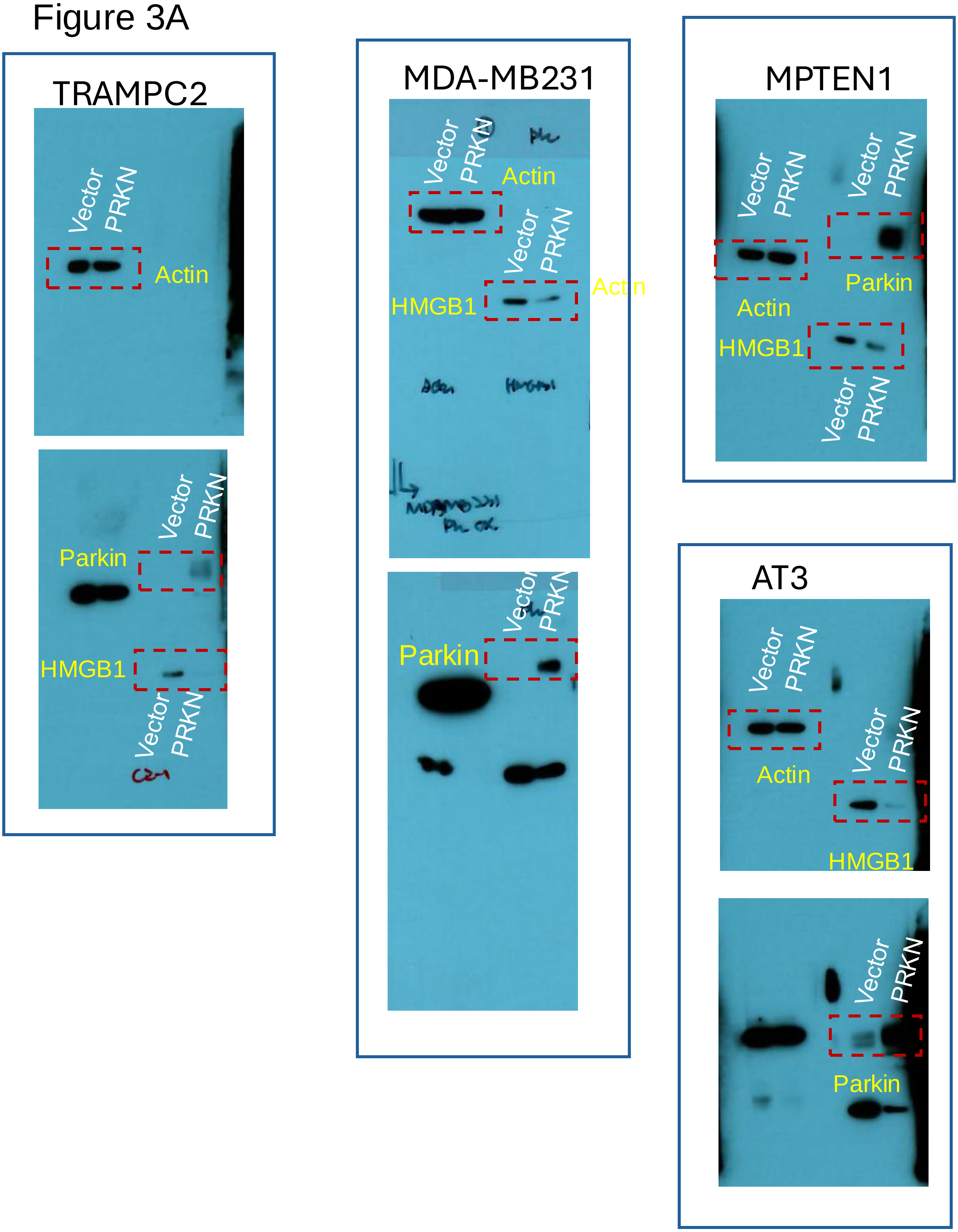
TRAMPC2
Vector
PRKN
Actin
Parkin
HMGB1
MDA-MB231
MPTEN1
AT3

Figure 3B

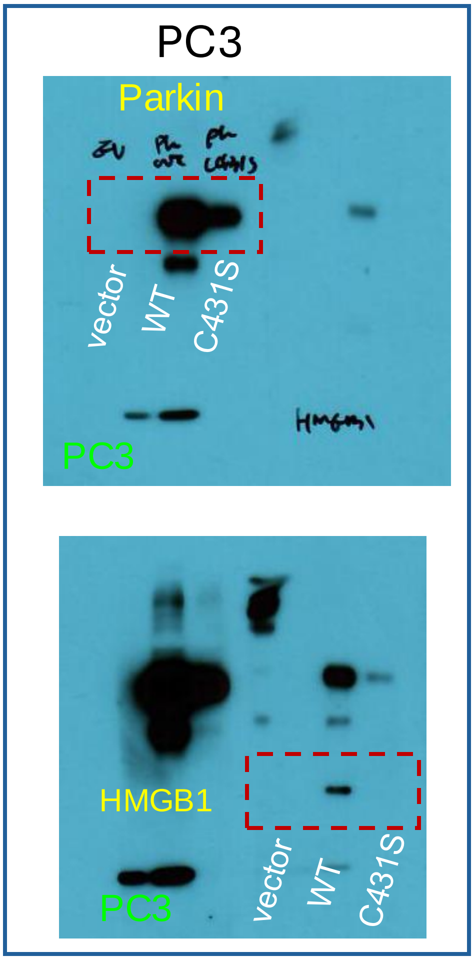

MP3098

Figure 3D

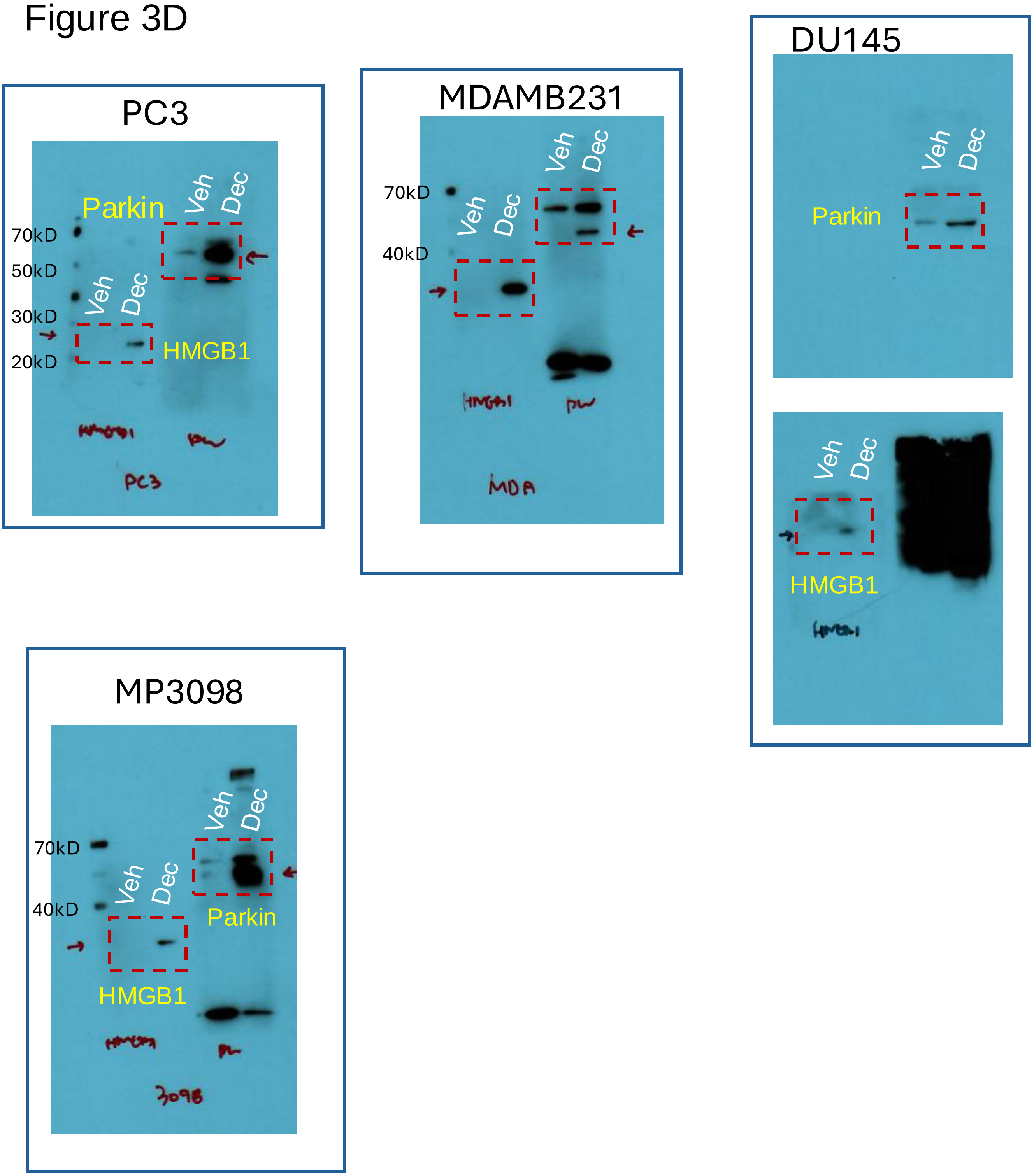
PC3
Parkin
Veh
Dec
70kD
50kD
30kD
20kD
HMGB1
MDAMB231
70kD
40kD
DU145
MP3098

Figure 3F

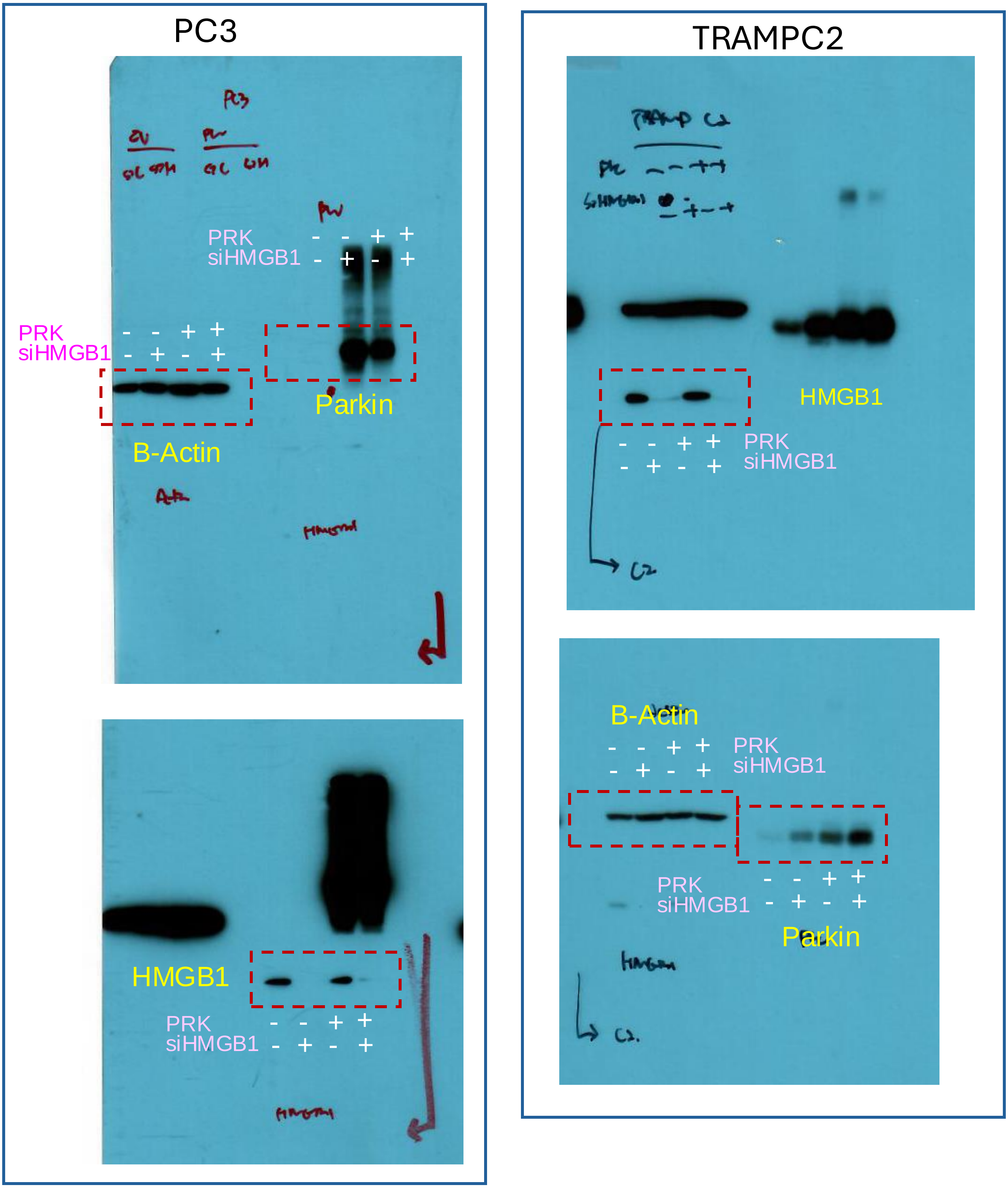

PC3
PRK - - + +
siHMGB1 - + - +
Parkin
B-Actin
HMGB1
TRAMPC2
HMGB1
B-Actin
Parkin

Figure 3H

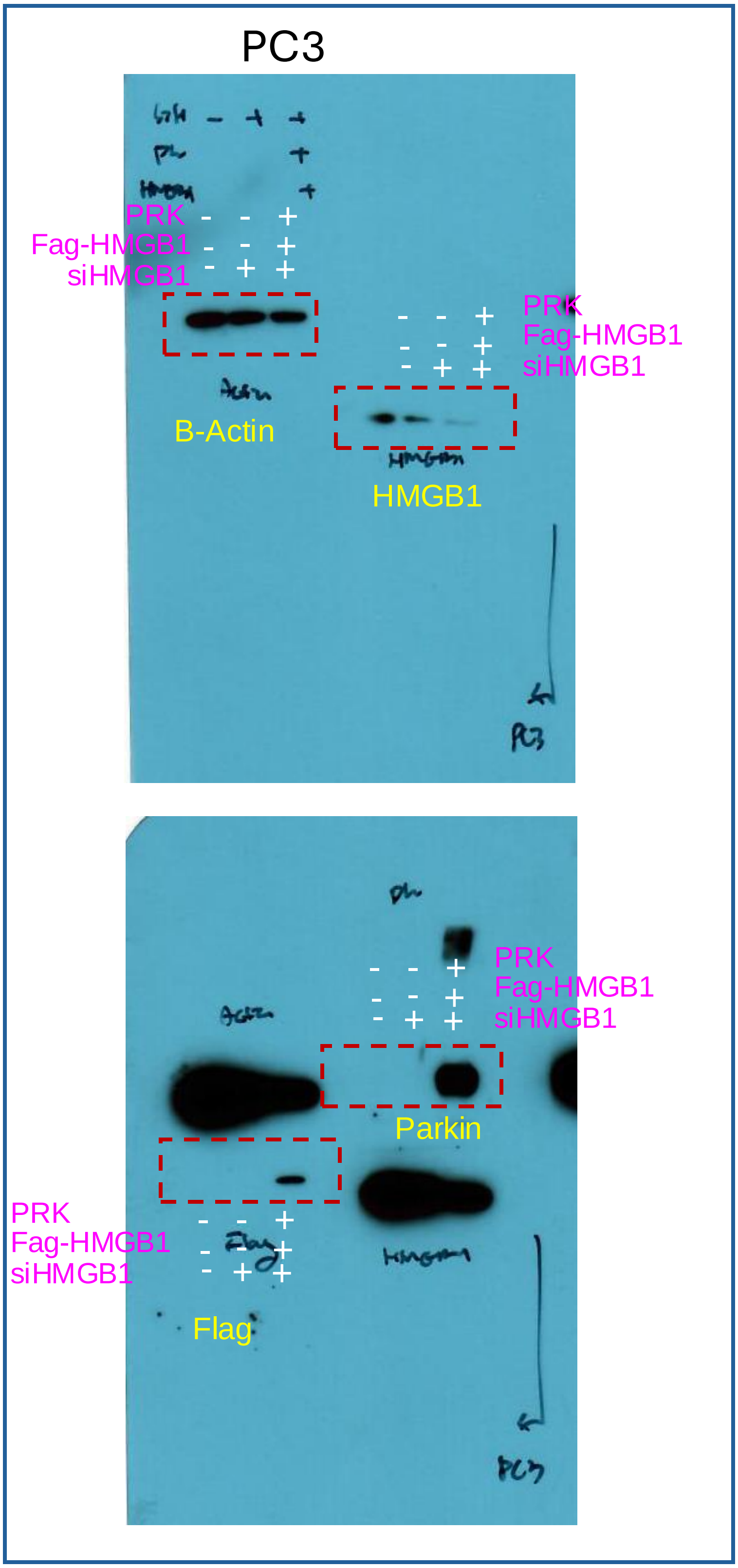

Figure S1G

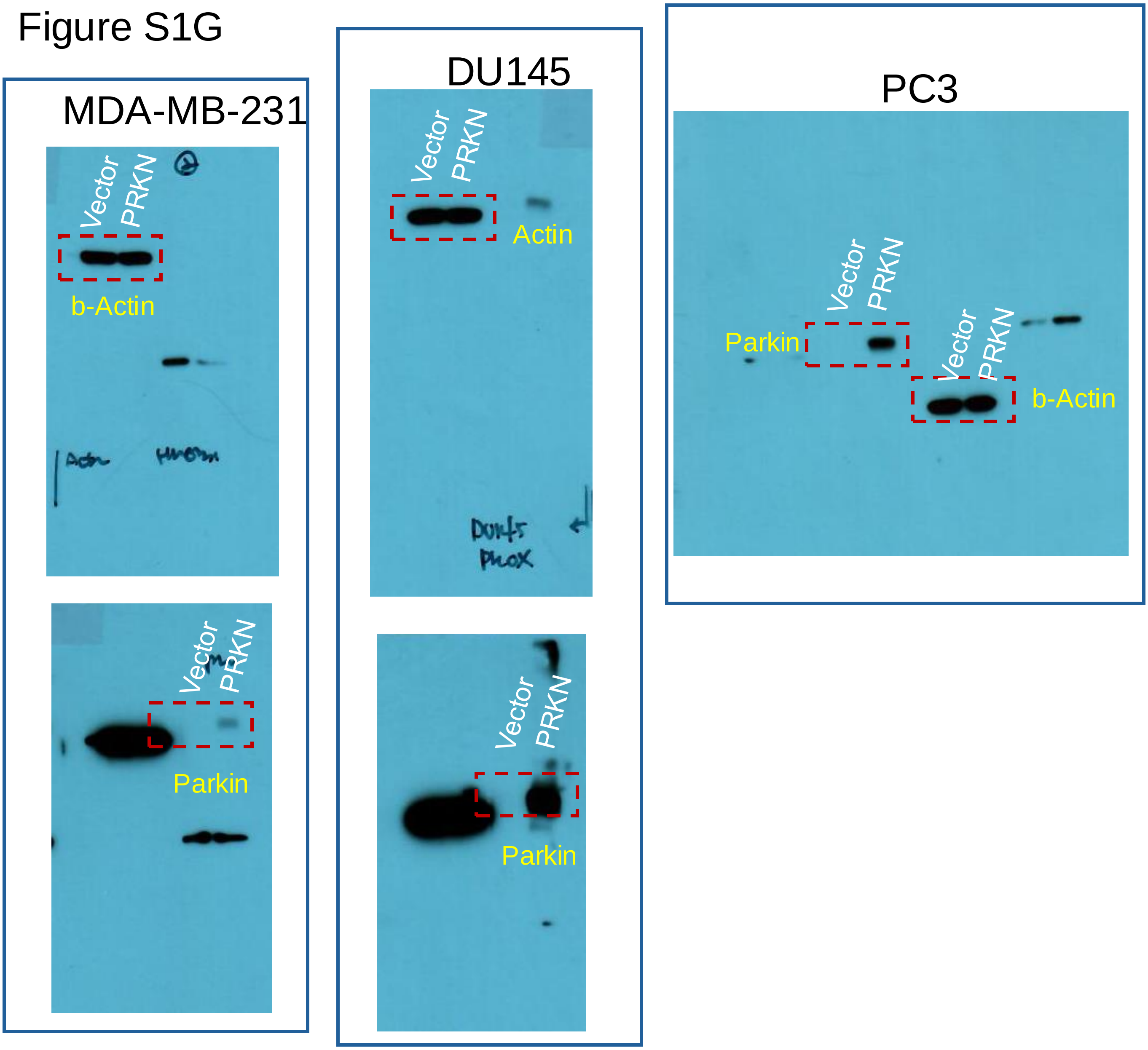

MDA-MB-231
Vector
PRKN
b-Actin
Vector
PRKN
Parkin
DU145
Vector
PRKN
Actin
Vector
PRKN
Parkin
PC3
Vector
PRKN
Parkin
Vector
PRKN
b-Actin

Figure S1G

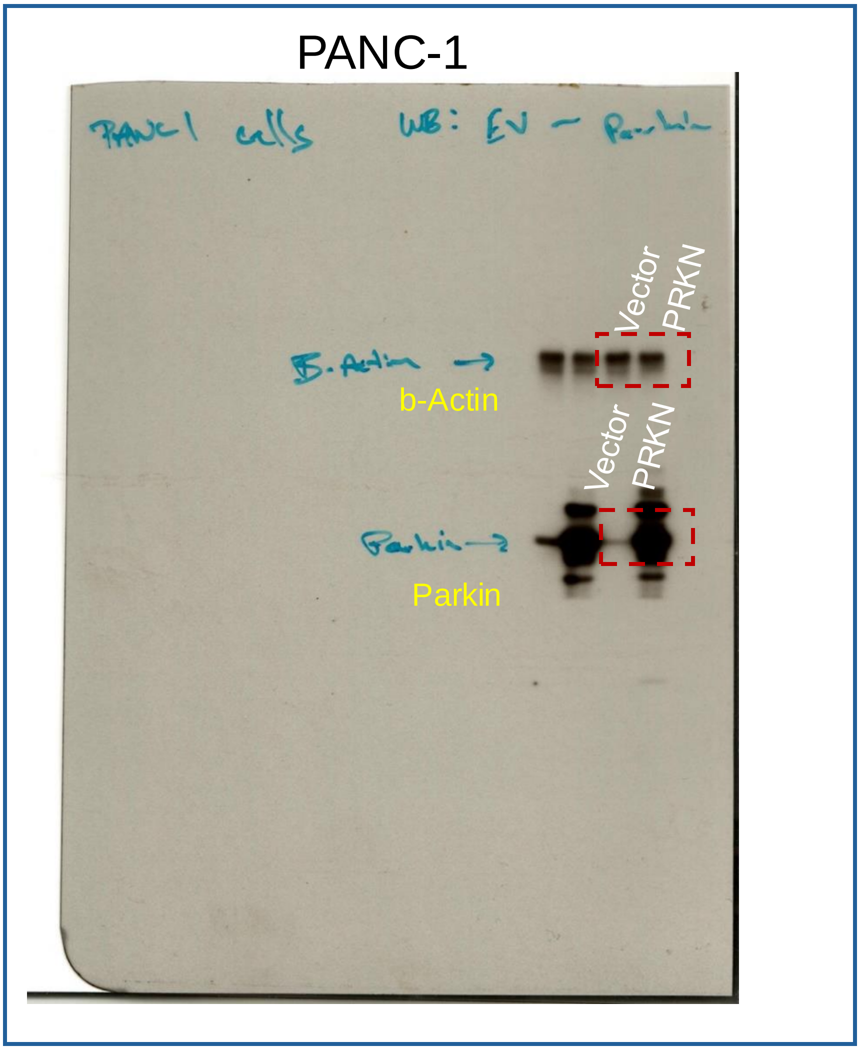
PANC-1
PANC1 cells
WB: EV – Parkin
Vector
PRKN
β-Actin →
b-Actin
Vector
PRKN
Parkin →
Parkin

Figure S2B

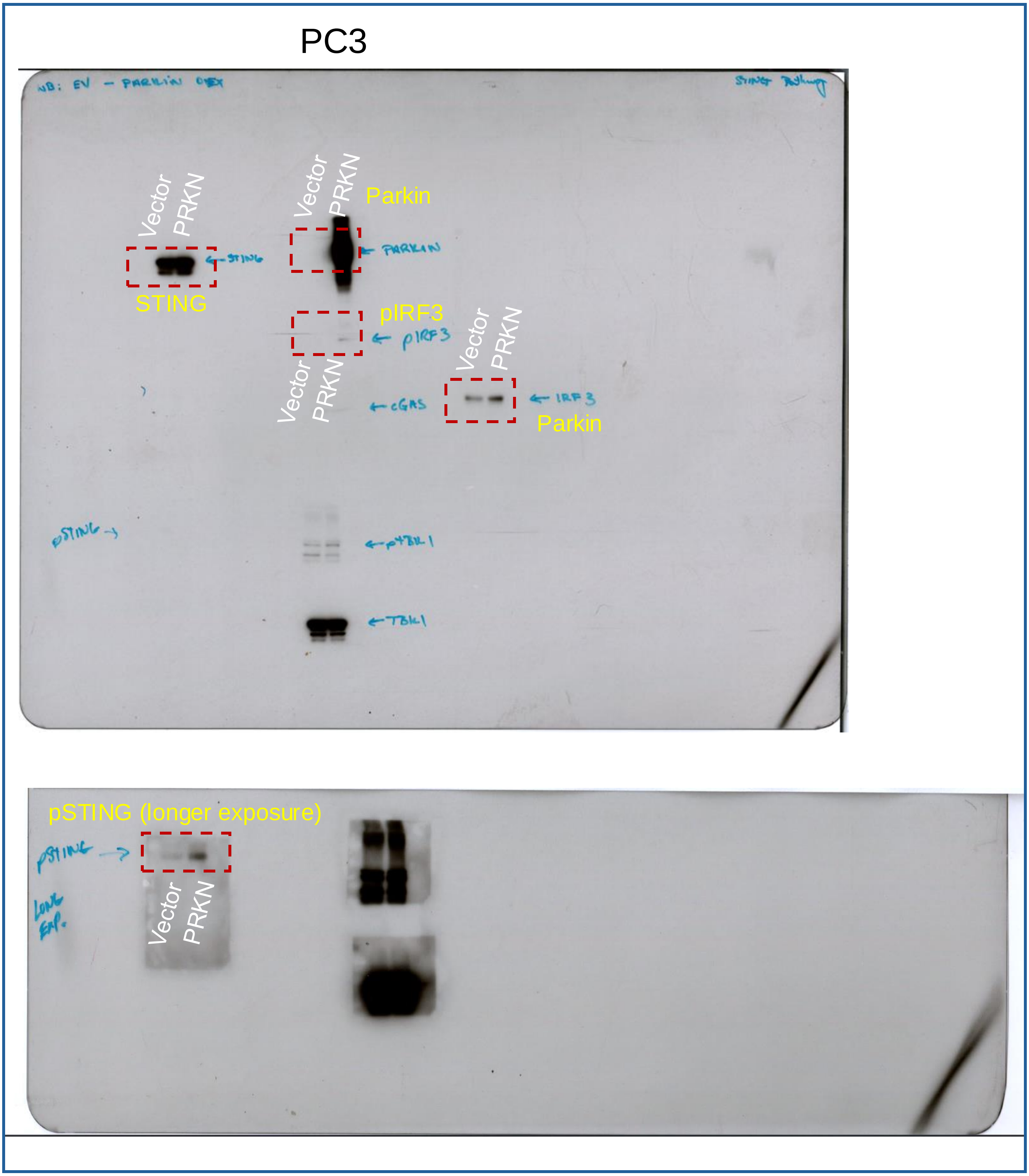
PC3
WB: EV – PARKIN OEX
STING Pathway
Vector
PRKN
STING
Parkin
PARKIN
pIRF3
pIRF3
IRF3
Parkin
cGAS
pSTING
pTBK1
TBK1
pSTING (longer exposure)
pSTING
LONG EXP.

Figure S2C-D

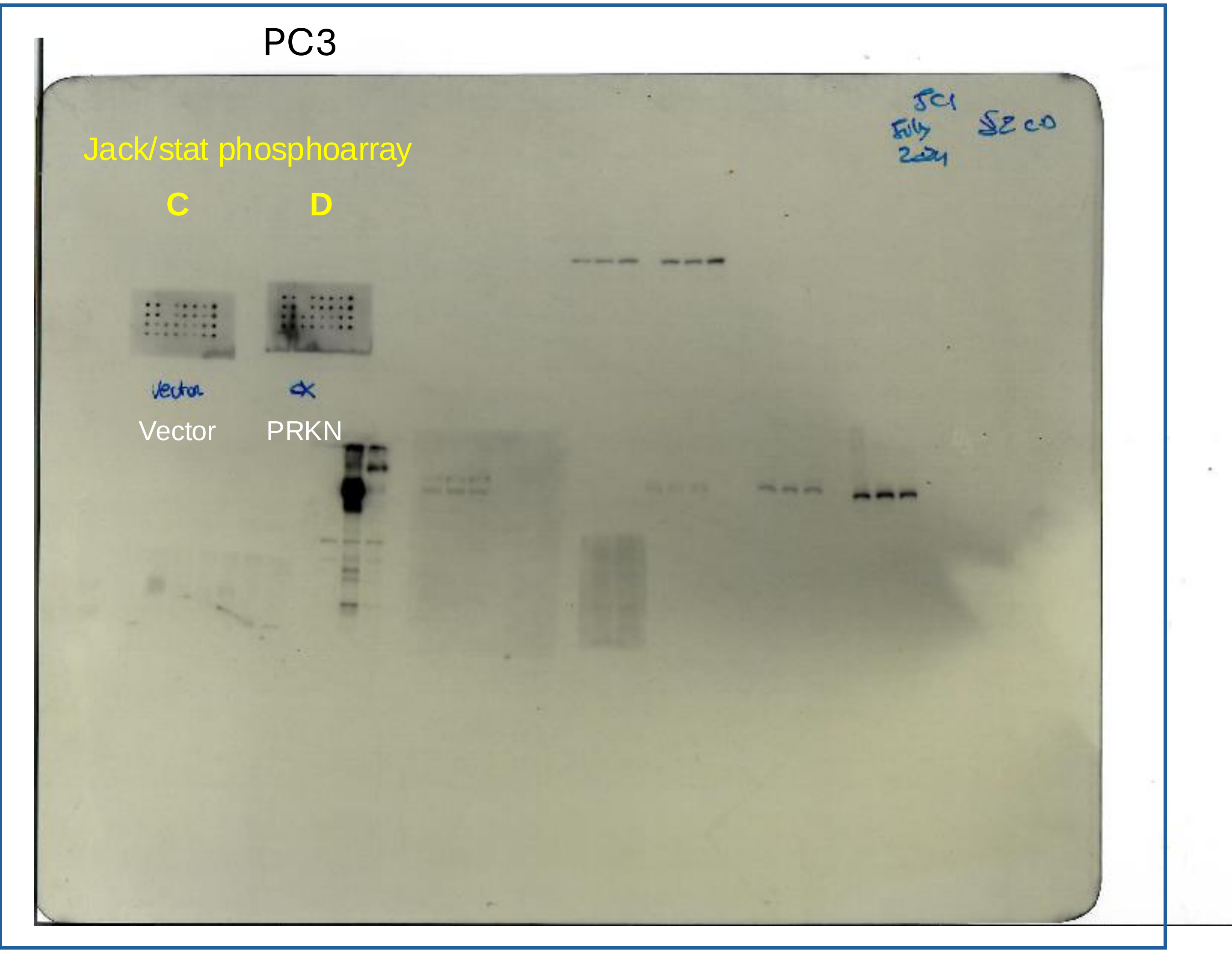

Figure S7A

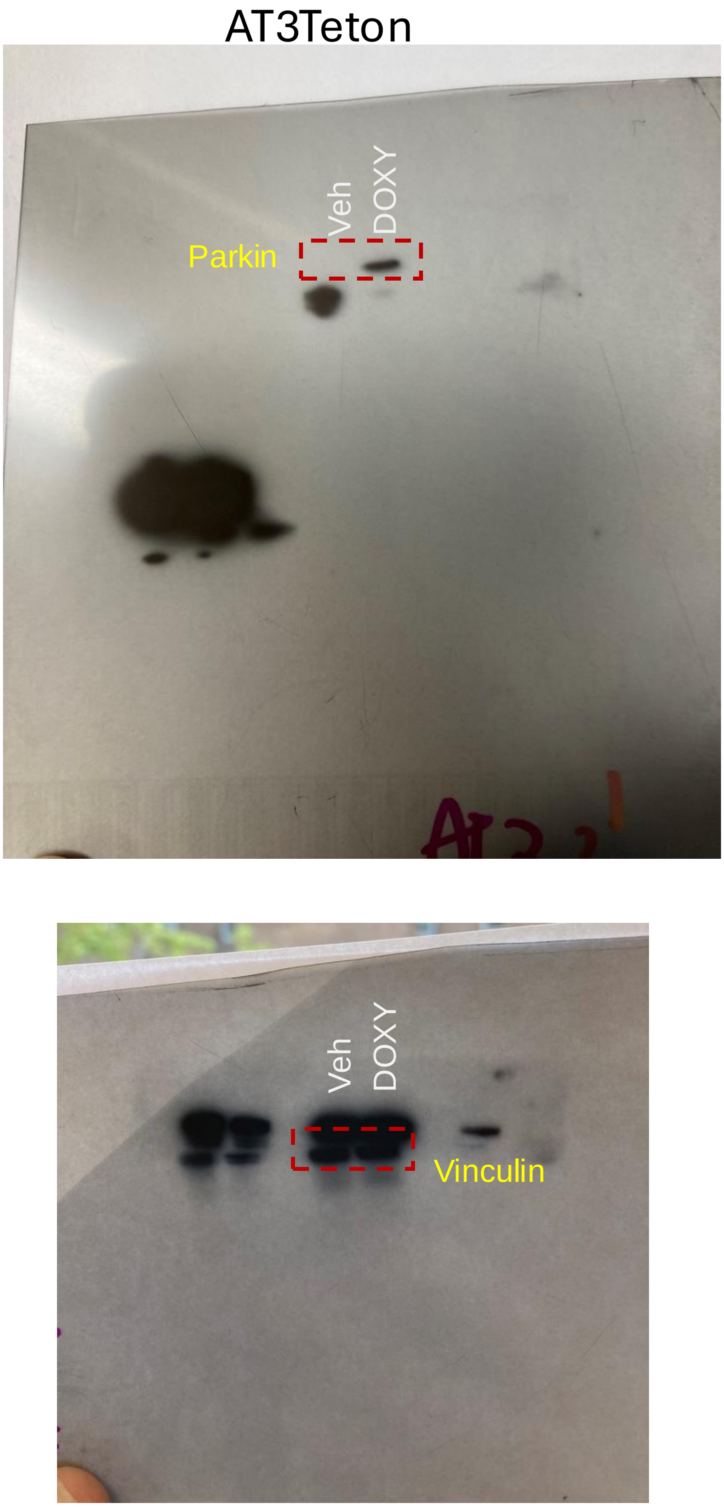

Supplement: Unedited blot and gel images [file jci-134-180983-s008.pdf]
