# Supplementary material for: Parkin activates innate immunity and promotes antitumor immune responses
Source: J Clin Invest. 2025 Jan 16;135(2):e190291. doi: 10.1172/JCI190291 (PMC11735089; doi:10.1172/JCI190291)

# Figure 1F

PC3 Teton

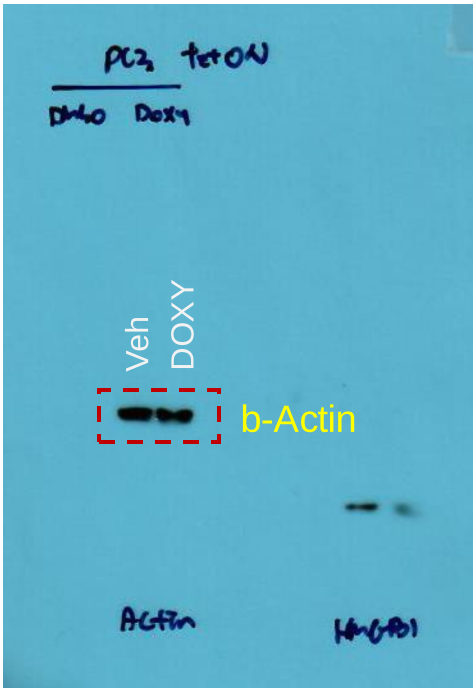


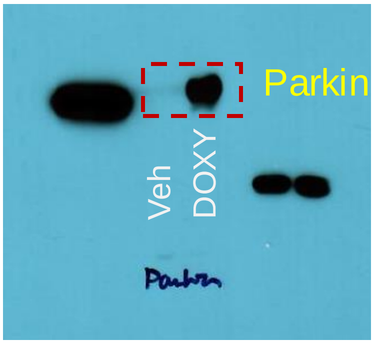

# Figure 1J

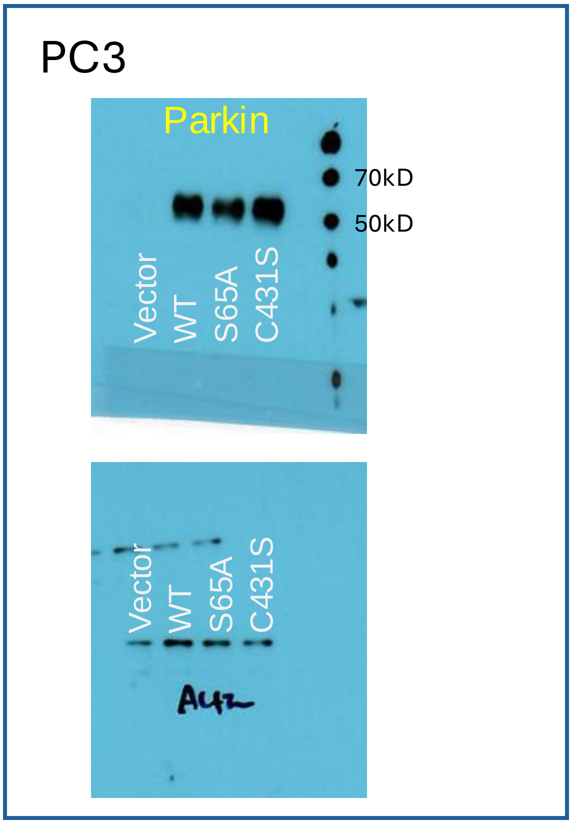
PC3
Parkin
70kD
50kD
Vector
WT
S65A
C431S
Vector
WT
S65A
C431S

# Figure 2E

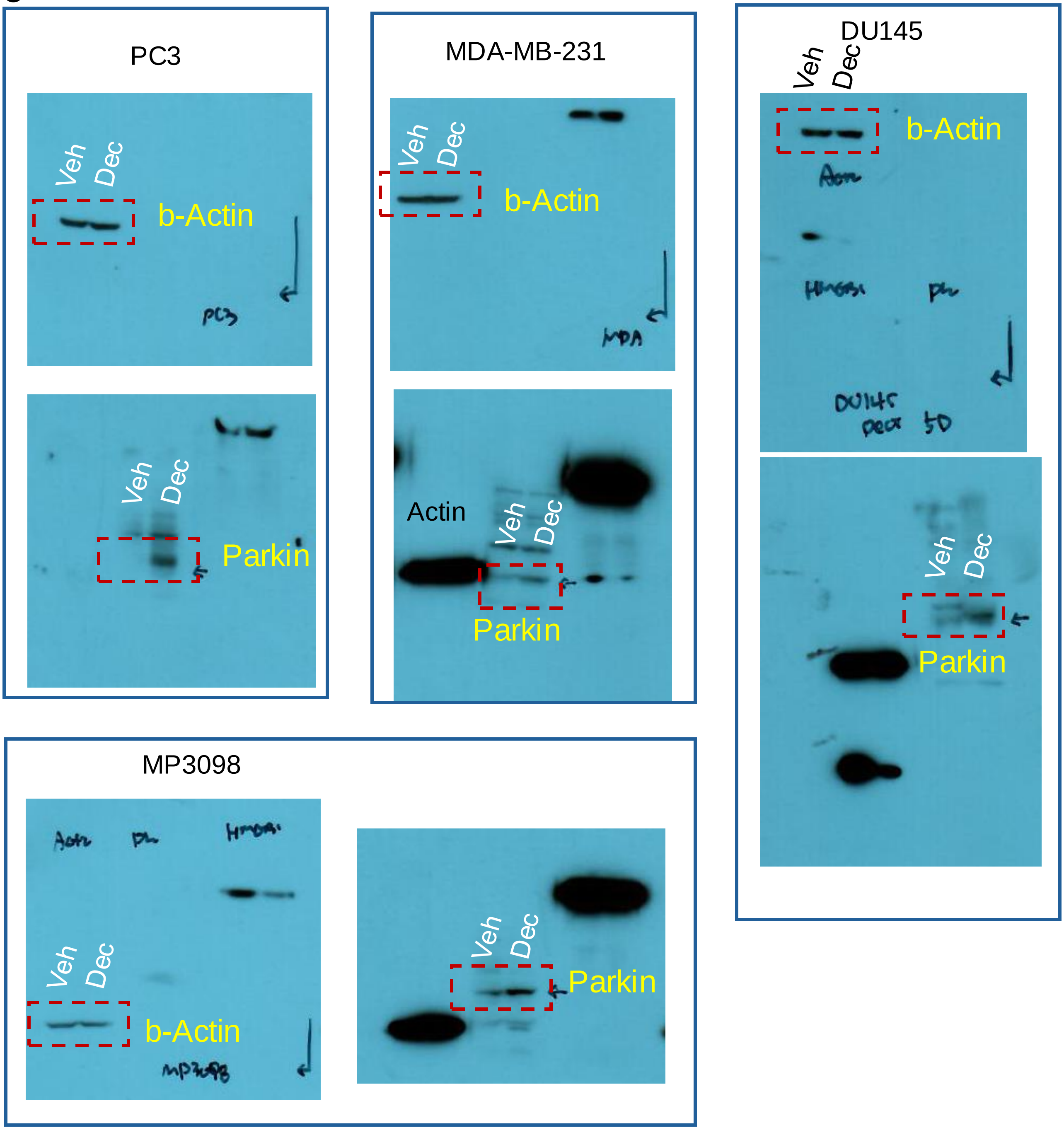

PC3
Veh
Dec
b-Actin
Parkin
MDA-MB-231
Veh
Dec
b-Actin
Actin
Veh
Dec
Parkin
DU145
Veh
Dec
b-Actin
Veh
Dec
Parkin
MP3098
Veh
Dec
b-Actin
Veh
Dec
Parkin

Figure 3A

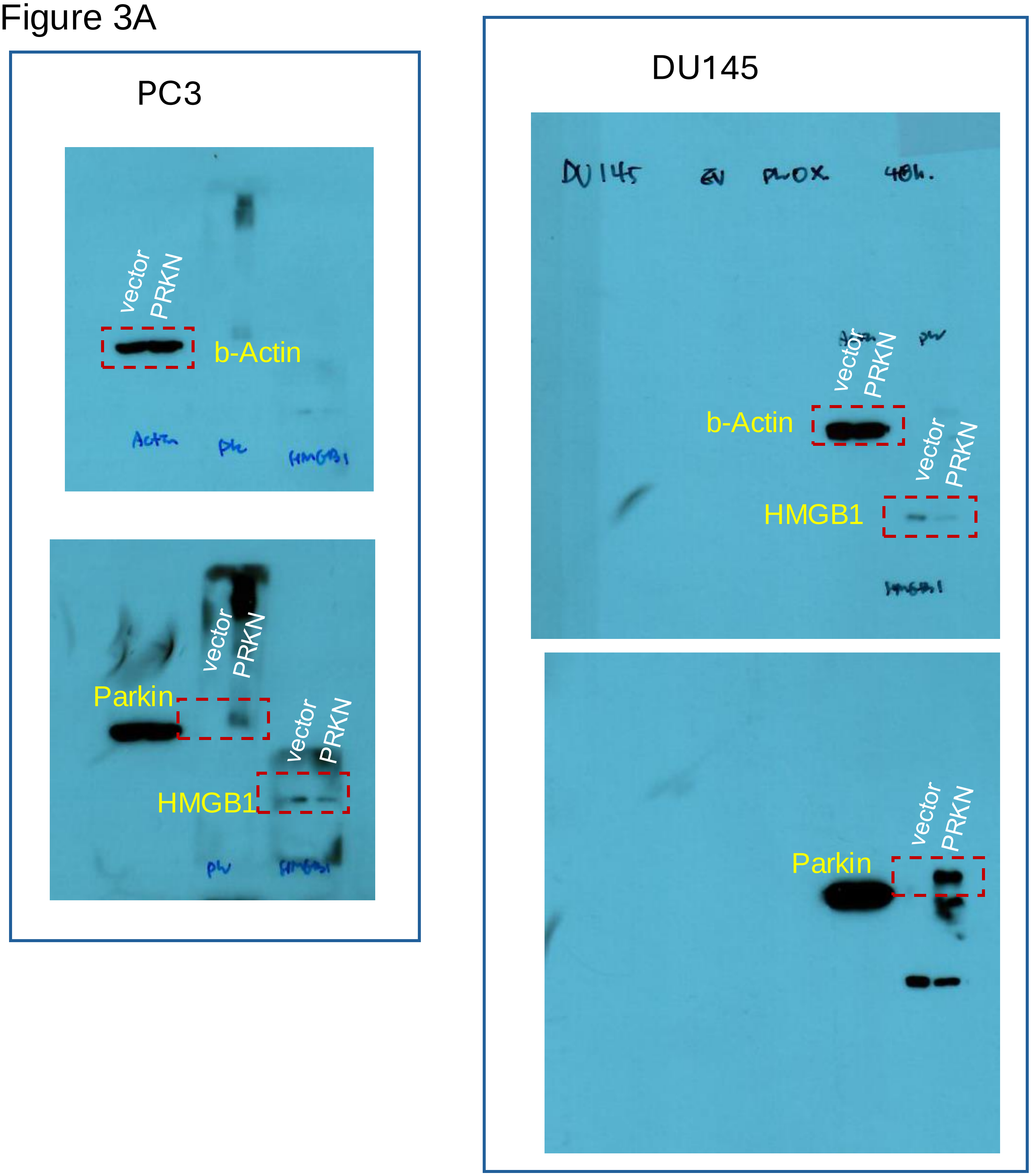
PC3
vector
PRKN
b-Actin
Parkin
HMGB1
DU145
DU145 EV PLOX 48h.
b-Actin
HMGB1
Parkin

Figure 3A

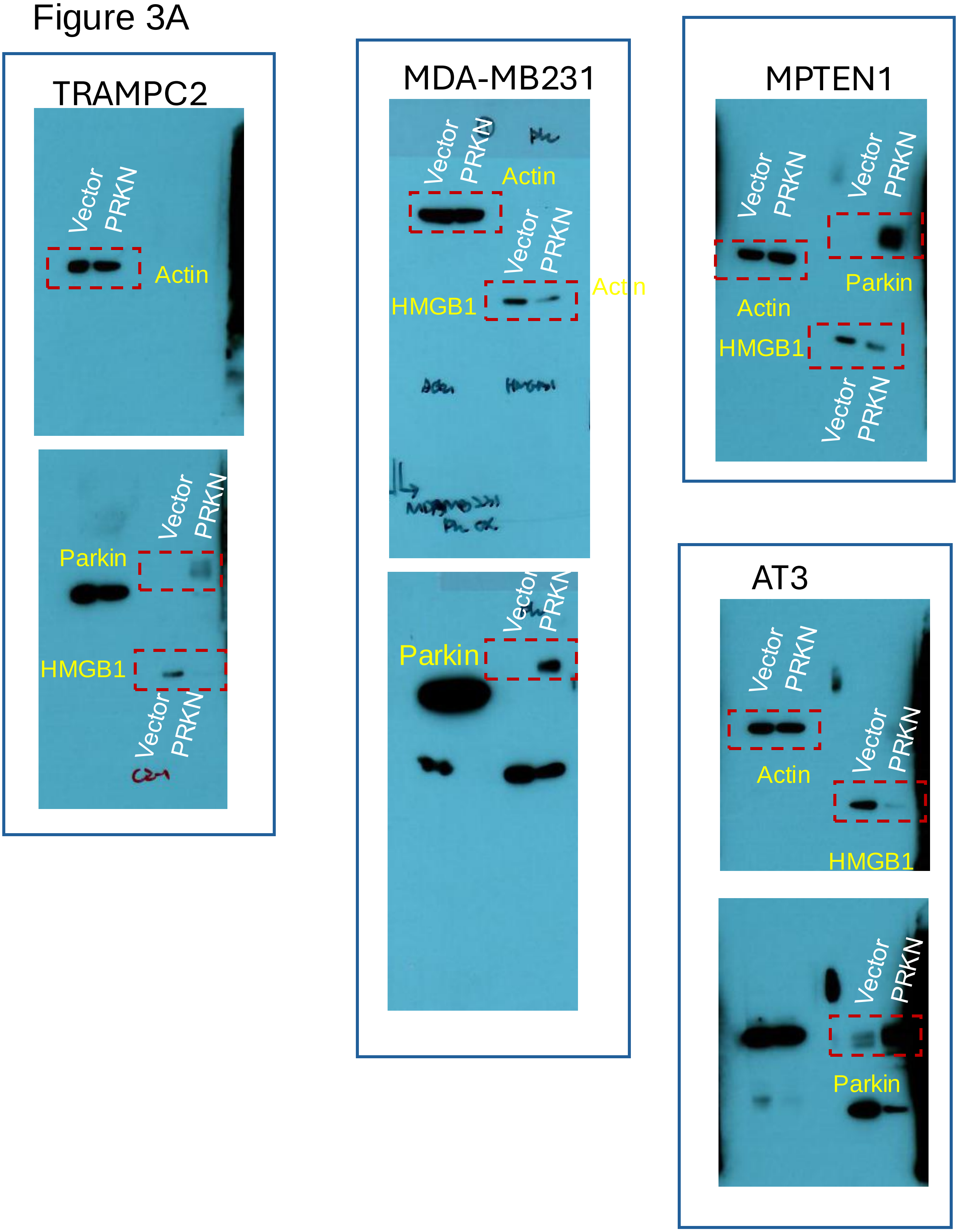

TRAMPC2
Vector
PRKN
Actin
Parkin
HMGB1
MDA-MB231
Actin
HMGB1
Parkin
MPTEN1
Actin
Parkin
HMGB1
AT3
Actin
HMGB1
Parkin

# Figure 3B

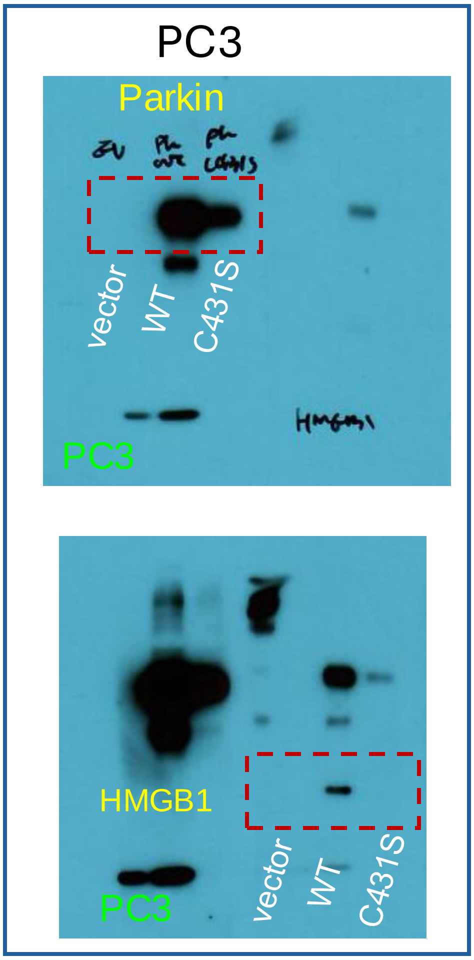


MP3098

Figure 3D

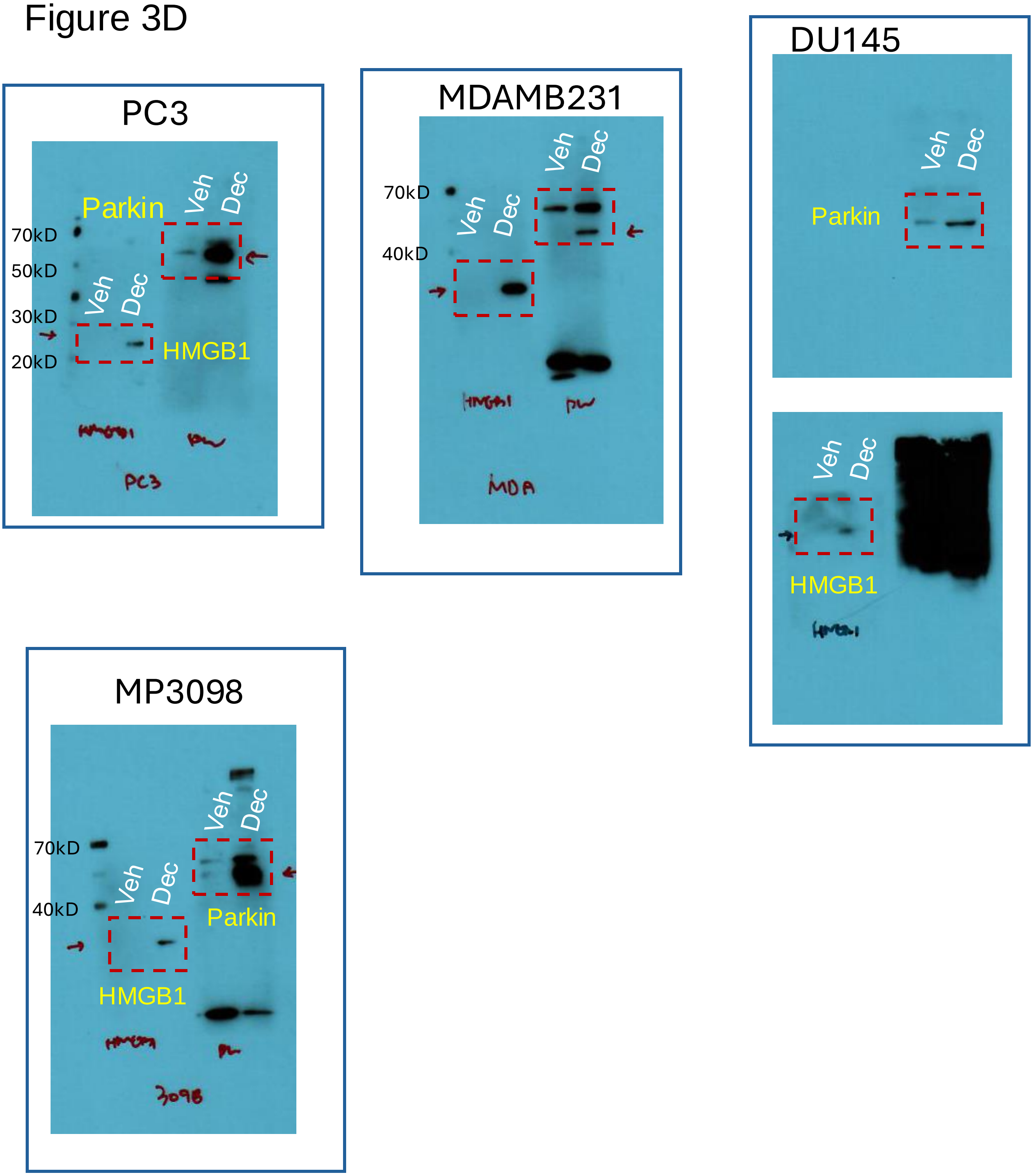
PC3
Parkin
Veh
Dec
70kD
50kD
30kD
20kD
Veh
Dec
HMGB1
MDAMB231
Veh
Dec
70kD
40kD
Veh
Dec
DU145
Veh
Dec
Parkin
Veh
Dec
HMGB1
MP3098
Veh
Dec
70kD
Veh
Dec
40kD
Parkin
HMGB1

# Figure 3F

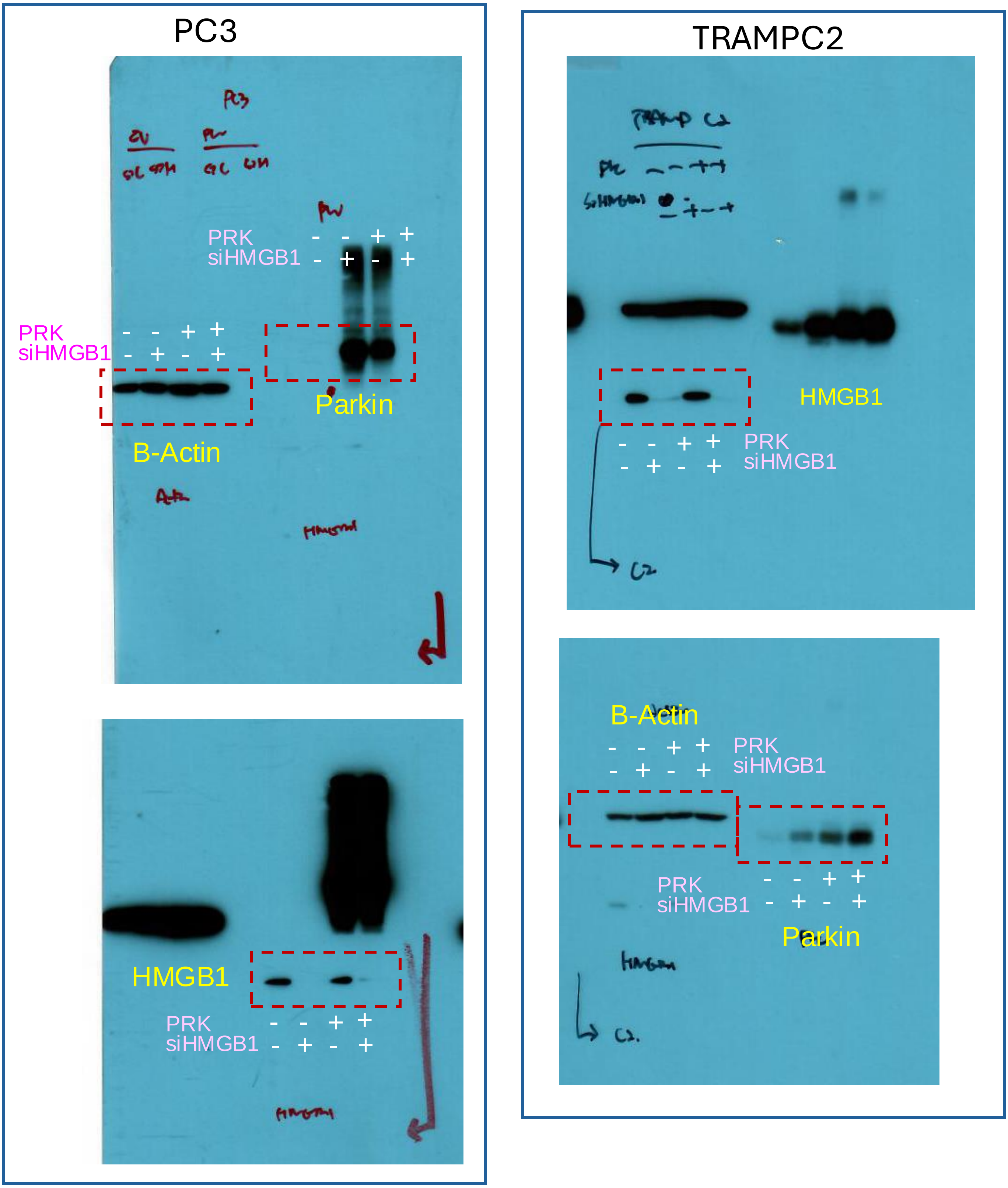

Figure 3H

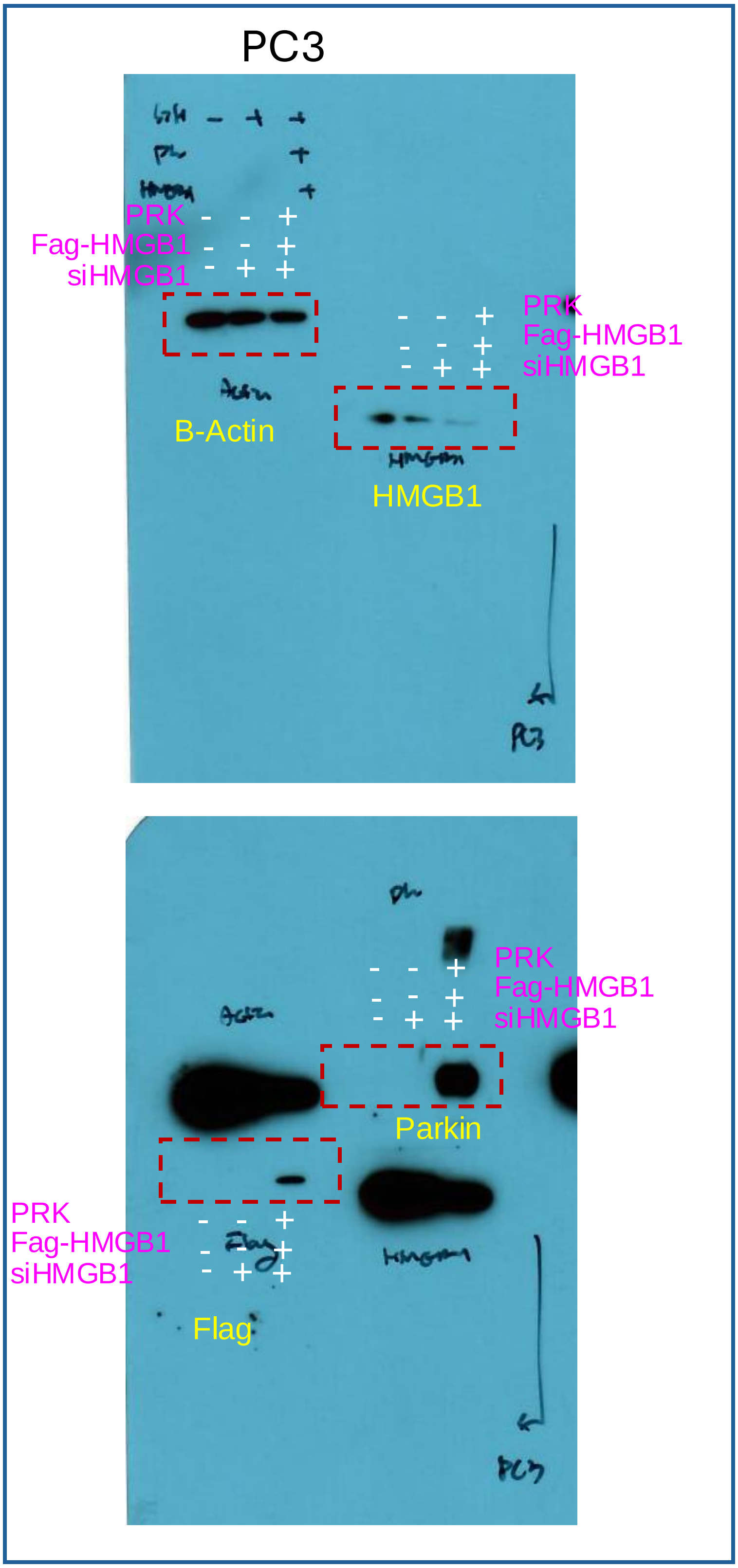

PC3
PRK - - +
Fag-HMGB1 - - +
siHMGB1 - + +
B-Actin
PRK - - +
Fag-HMGB1 - - +
siHMGB1 - + +
HMGB1
PRK - - +
Fag-HMGB1 - - +
siHMGB1 - + +
Parkin
PRK - - +
Fag-HMGB1 - - +
siHMGB1 - + +
Flag

Figure S1G

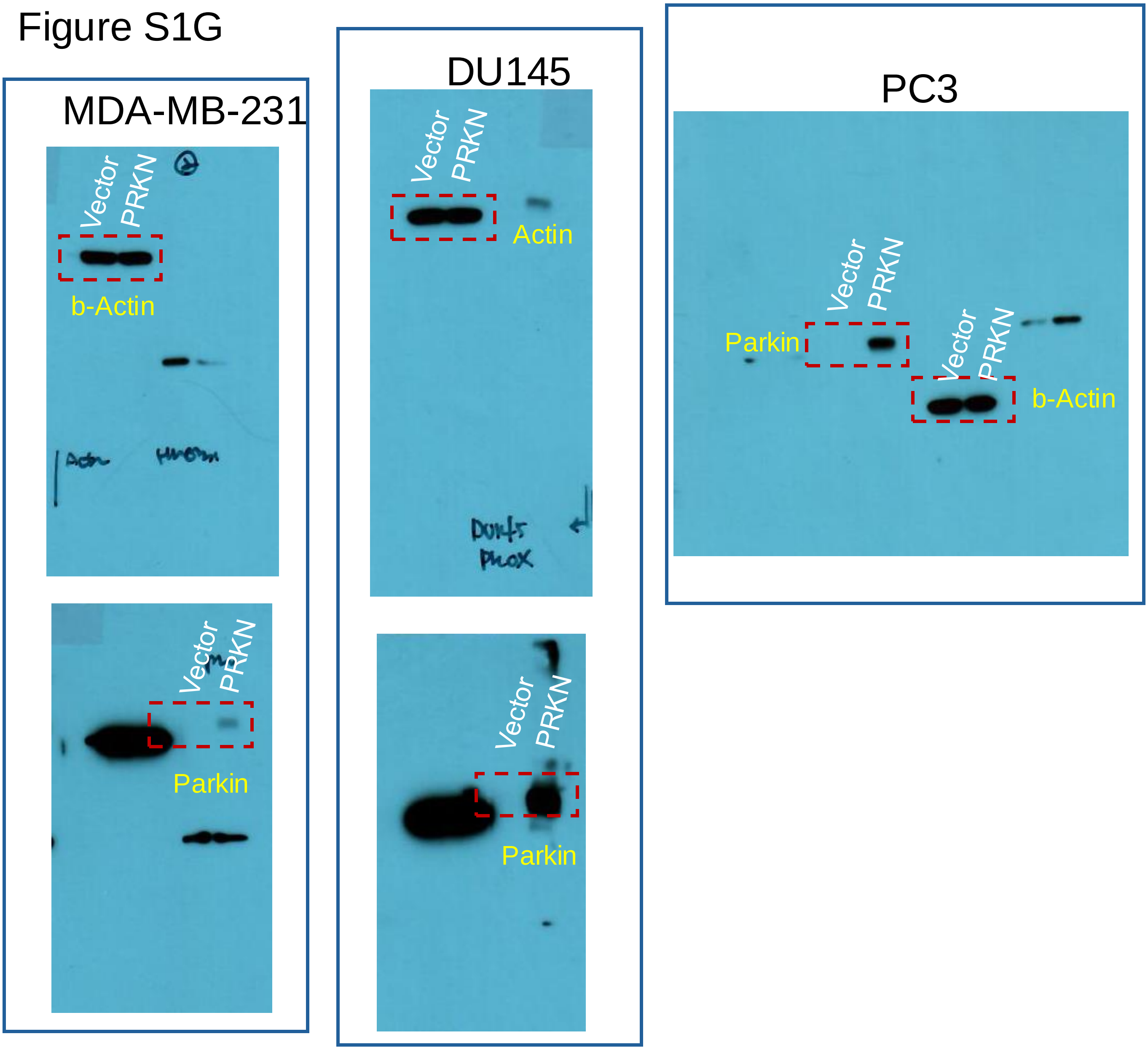
MDA-MB-231
Vector
PRKN
b-Actin
Vector
PRKN
Parkin
DU145
Vector
PRKN
Actin
Vector
PRKN
Parkin
PC3
Vector
PRKN
Parkin
Vector
PRKN
b-Actin

Figure S1G

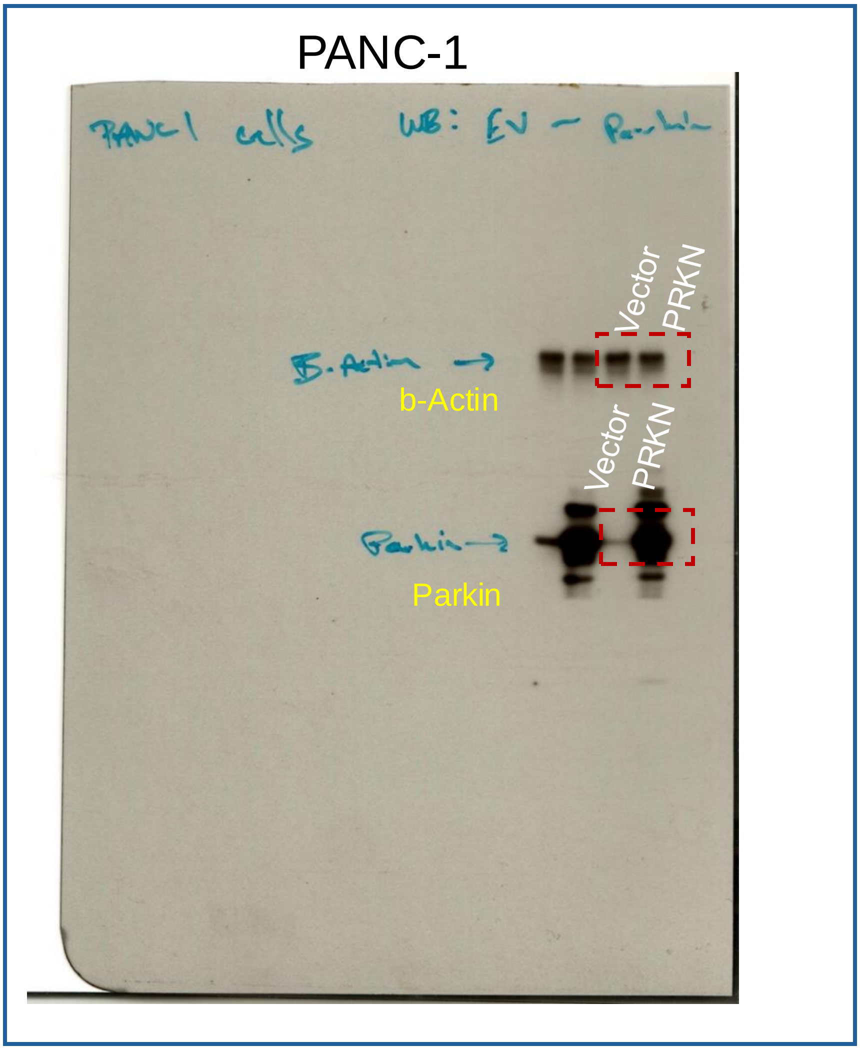
PANC-1
PANC1 cells WB: EV — Parkin
Vector
PRKN
B-Actin →
b-Actin
Vector
PRKN
Parkin →
Parkin

Figure S2B

PC3

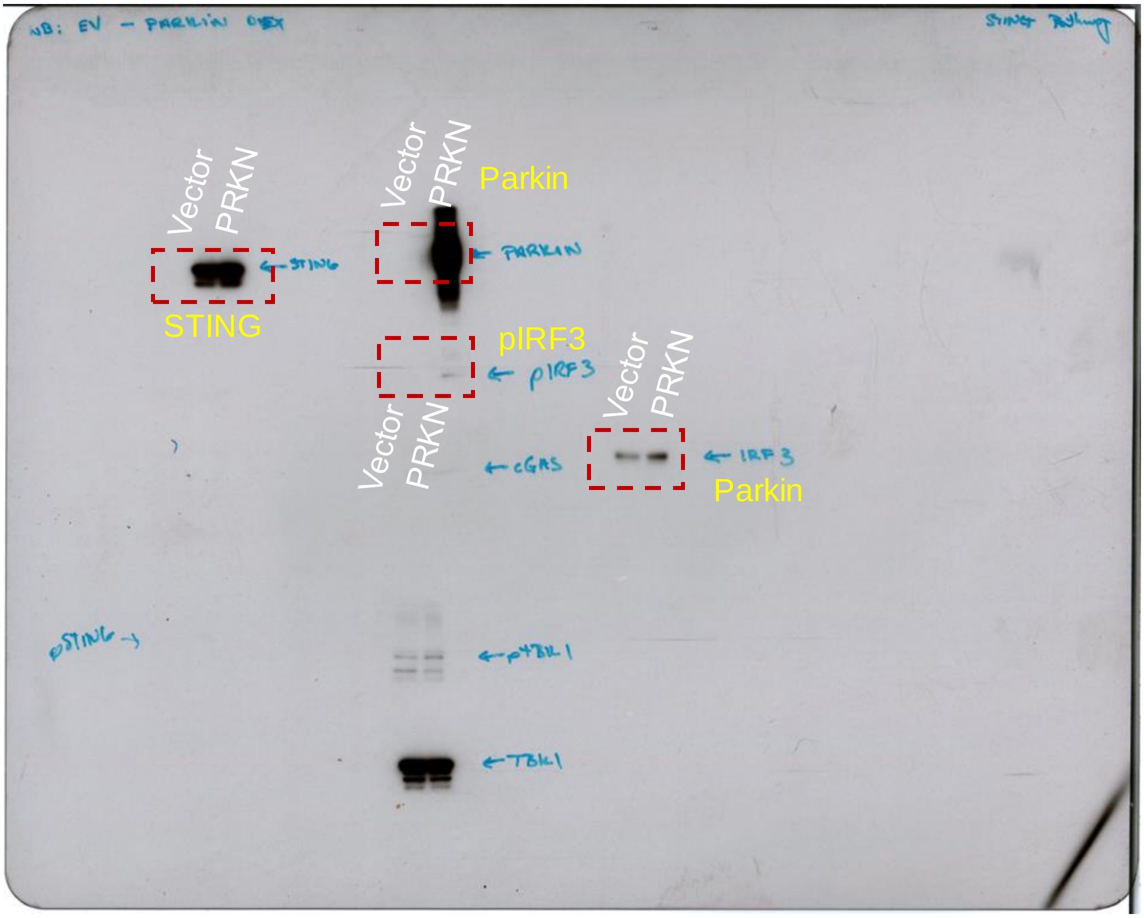


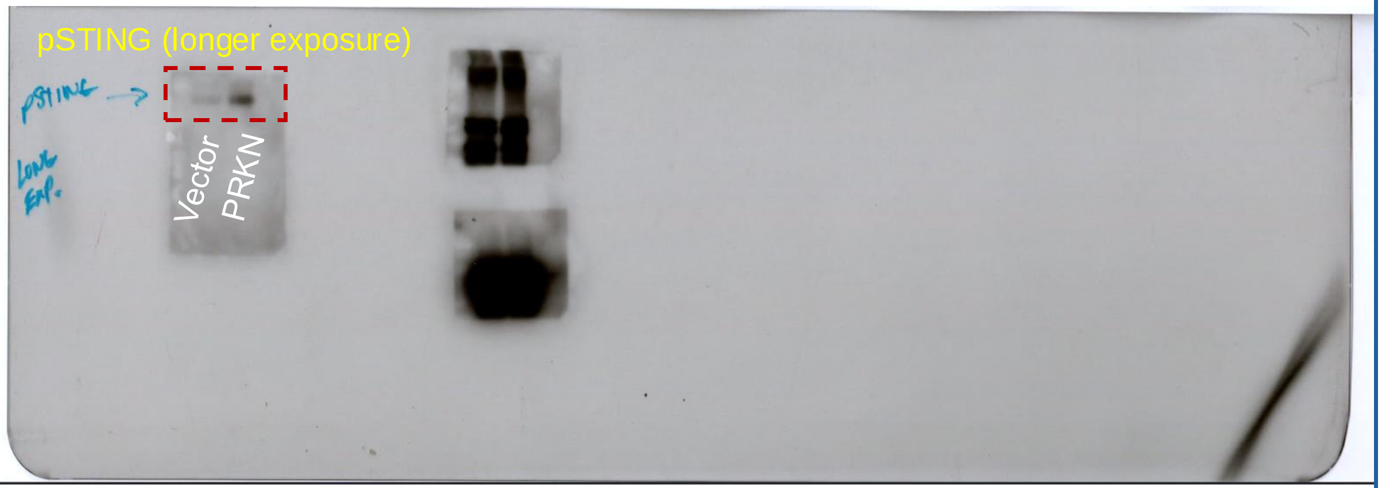

# Figure S2C-D

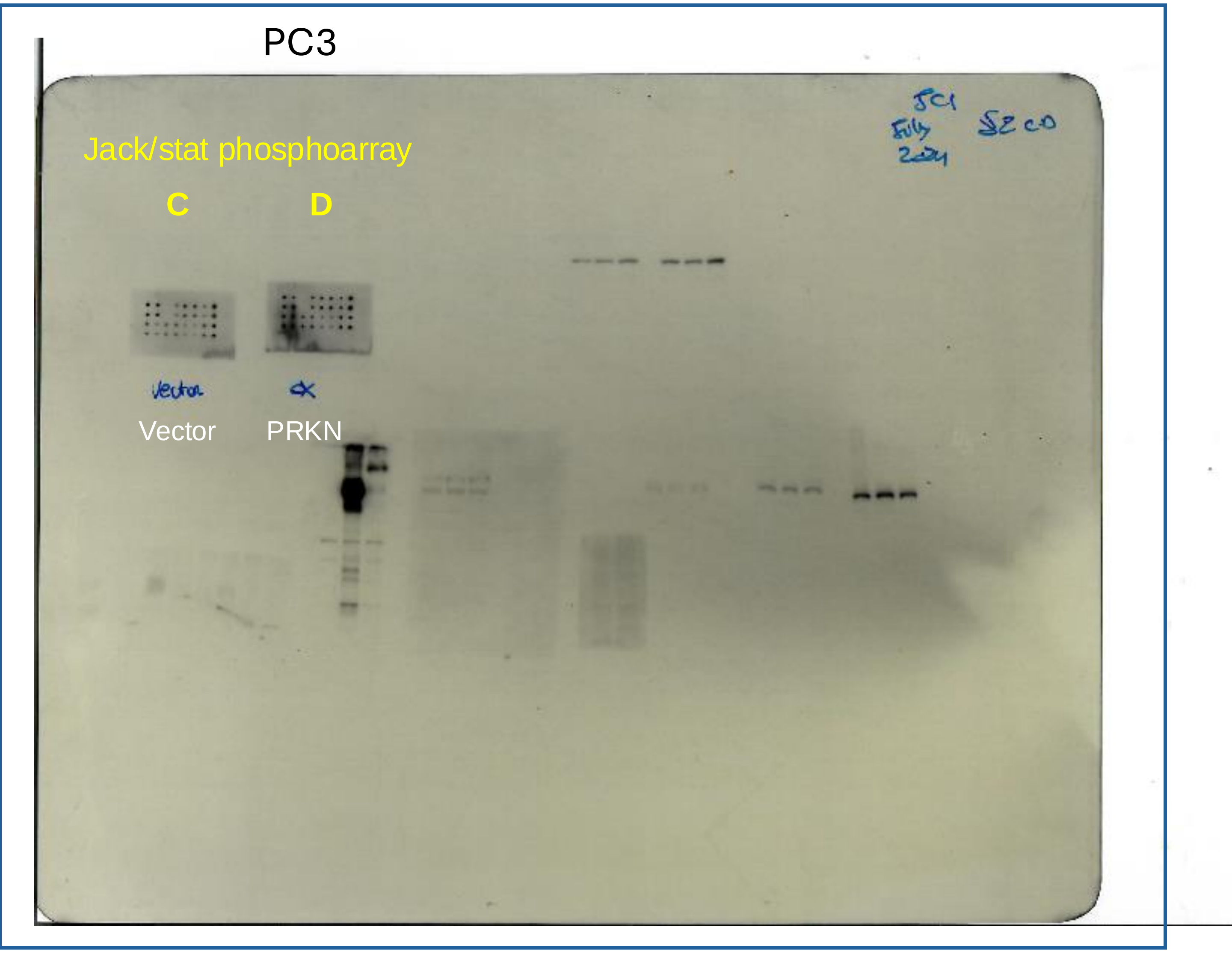

Figure S7A

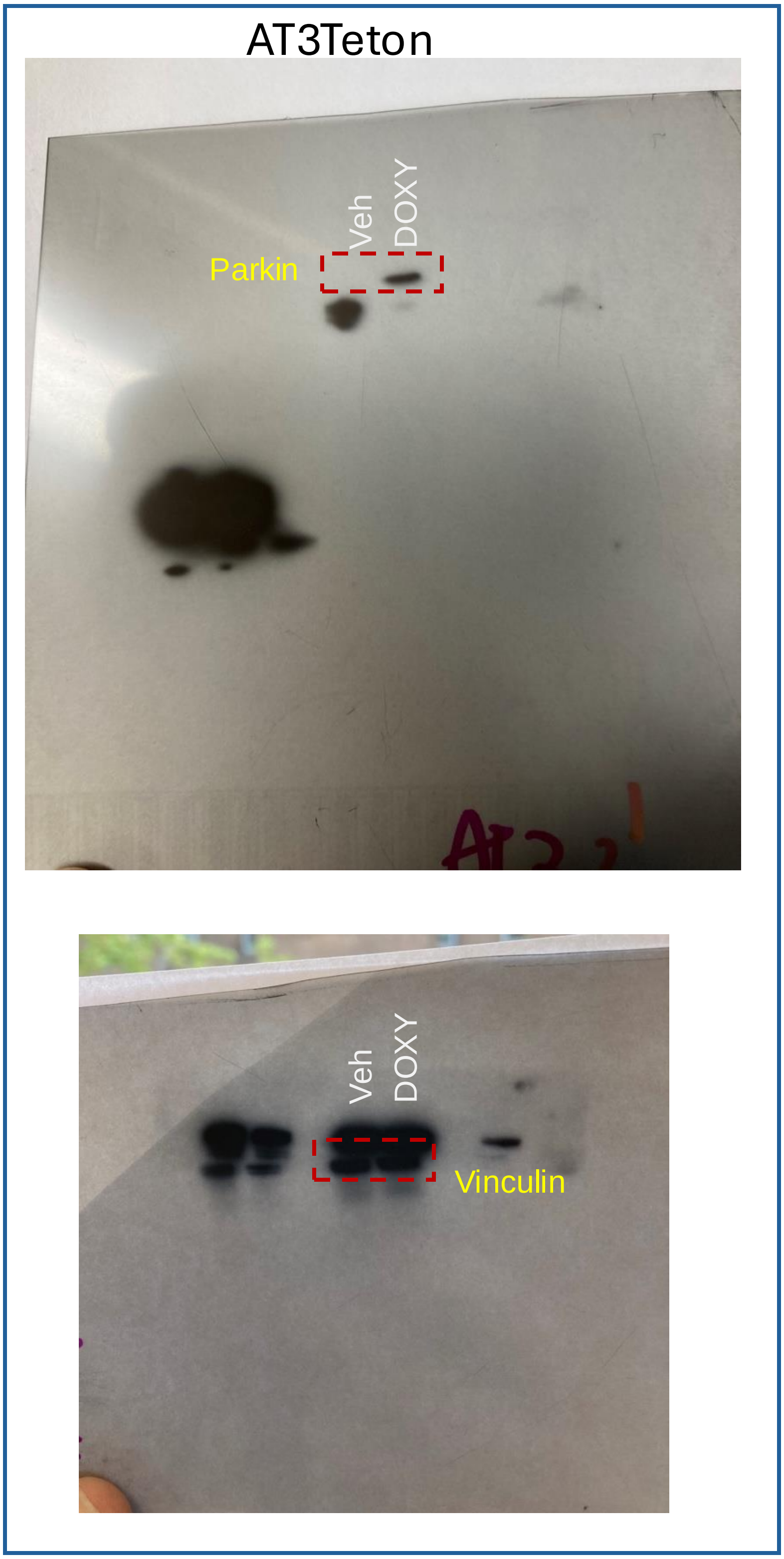

Supplement: Unedited blot and gel images [file jci-135-190291-s002.pdf]
